# Microwave-assisted synthesis and functionalization of 2-arylimidazo[1,2-*a*]pyrimidin-5(8*H*)-ones†

**DOI:** 10.1039/d4ra03948c

**Published:** 2024-07-15

**Authors:** Delascar Camargo, Carlos Cifuentes, Juan-Carlos Castillo, Jaime Portilla

**Affiliations:** a Bioorganic Compounds Research Group, Department of Chemistry, Universidad de Los Andes Carrera 1 No. 18A-10 Bogotá 111711 Colombia jportill@uniandes.edu.co; b Escuela de Ciencias Químicas, Universidad Pedagógica y Tecnológica de Colombia Avenida Central del Norte 39-115 Tunja Colombia

## Abstract

Despite the limited applications and scarcity of commercial examples of imidazo[1,2-*a*]pyrimidines, their exceptional properties hold great potential, representing a significant challenge in discovering more critical applications. Herein, we present a microwave-assisted approach for preparing 2-arylimidazo[1,2-*a*]pyrimidin-5(8*H*)-ones and their alkylation and bromination products using easily accessible and inexpensive reagents, thus offering a promising avenue for further search. Notably, the photophysical properties of an *N*-alkyl derivative were investigated, and the results highlight the high potential of these compounds as modular fluorophores. All the products were obtained with high yields using highly efficient protocols, and the regioselectivity of the reactions was determined on the basis of NMR measurements and X-ray diffraction analysis.

## Abstract 2

MW-assisted synthesis of 2-arylimidazo[1,2-*a*]pyrimidin-5(8*H*)-ones and their *N*-alkylation and bromination products using easily accessible and cheap reagents. Obtaining a bioactive *N*-alkylated analog and photophysical study of a fluorescent product.

## Graphical abstract

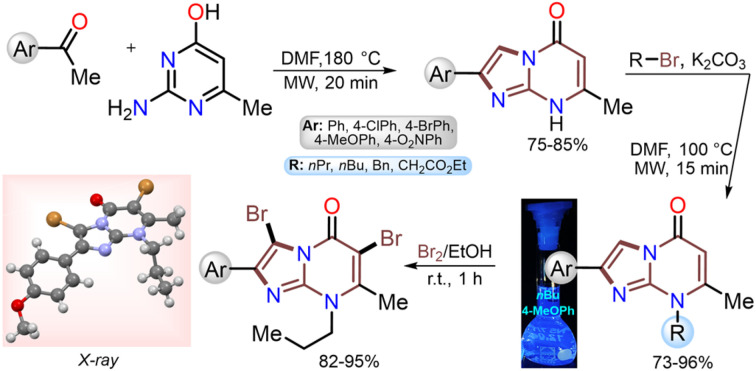

## Introduction

**Fig. 1:**
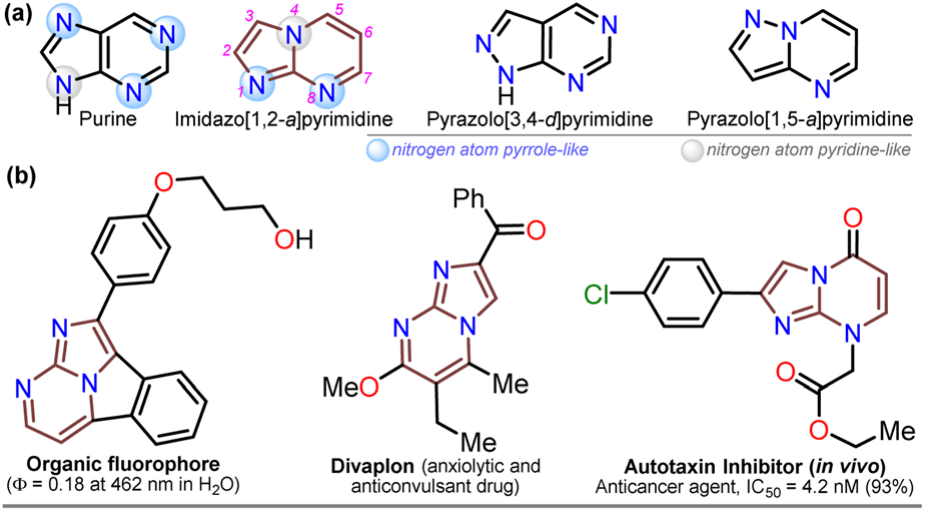
Molecular structures of (a) some diazolopyrimidine derivatives and (b) relevant imidazo[1,2-*a*]pyrimidines-based compounds.

For decades, the chemistry of N-heterocyclic compounds has been studied owing to their crucial role in the medicinal field, electronic properties conferred by heteroatoms, and their notable synthetic flexibility.^1–5^ In this context, diazolopyrimidines, which are structural analogs of purines typically found in biologically active compounds and, more recently, in organic fluorophores, have gained attention. They have two types of nitrogen atoms, pyrrole-like (–NR–) and (2 or 3) pyridine-like (=N–) with π-excessive and π-deficient characteristics, respectively, justifying their unique stereoelectronic properties (Fig. 1a).^6–10^ Although there are many works on imidazo[1,2-*a*]pyrimidine-ring-based compounds, this scaffold is part of diazolopyrimidines with minor applicability with few commercial compounds containing it. Thus, studying this type of compound is valuable since it has high and proven photophysical^9–11^ and biological potential^12–21^ (Fig. 1b).

**Scheme 1:**
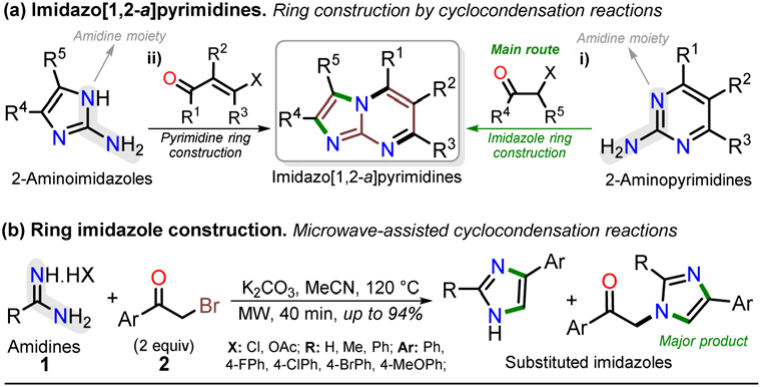
Synthesis of (a) imidazo[1,2-*a*]pyrimidines and (b) imidazoles from amidines.

Notably, the imidazo[1,2-*a*]pyrimidine (IP) motif has been found in molecules with antileishmanial,^12^ anticancer,^13,14,17^ antibacterial,^15^ and antiinflammatory^16^ activities, and various ways of synthesizing imidazo[1,2-*a*]pyrimidines (IPs) have been developed. However, in addition to articles^9–17,22–31^ and patents,^32,33^ only few specialized reviews^18–21^ on IPs are available. These compounds generally allow structural changes at positions 2, 3, or 5–8 *via* ring building or functionalization with aromatic substituents, redox, or metal-mediated C–C coupling reactions. IP ring construction is mostly achieved by cyclocondensation reactions of (i) 2-aminopyrimidines (amidine-type reagent) with 1,2-bis-electrophilic compounds (*e.g.*, α-haloketones and α-alkoxy ketones) or (ii) 2-aminoimidazoles with 1,3-bis-electrophiles (β-diketones, β-alkoxyenones, *etc.*), of which the former route is the principal method (Scheme 1a).^9–33^

Most approaches for the synthesis of imidazo[1,2-*a*]pyrimidines have the typical operational difficulties (*i.e.*, multistep synthetic procedures with low overall yields, poor availability or solubility of starting materials, tedious processes, long reaction times, and reactions mediated by expensive catalysts or additives).^9–33^ Therefore, developing efficient and alternative synthetic methods to obtain them from economic reagents using low energy is highly valuable. In this context, microwave (MW)-assisted synthesis is being intensively studied as these methods seem ideal and usually result in yields superior to conventional heating protocols.^7,34–38^ Indeed, many similar compounds are synthesized by our research group under microwave irradiation; for example, we obtained excellent results in the preparation of substituted imidazoles using amidines (1) (poorly soluble in organic solvents) and α-bromoacetophenones (2) (Scheme 1b).^36–38^

**Scheme 2:**
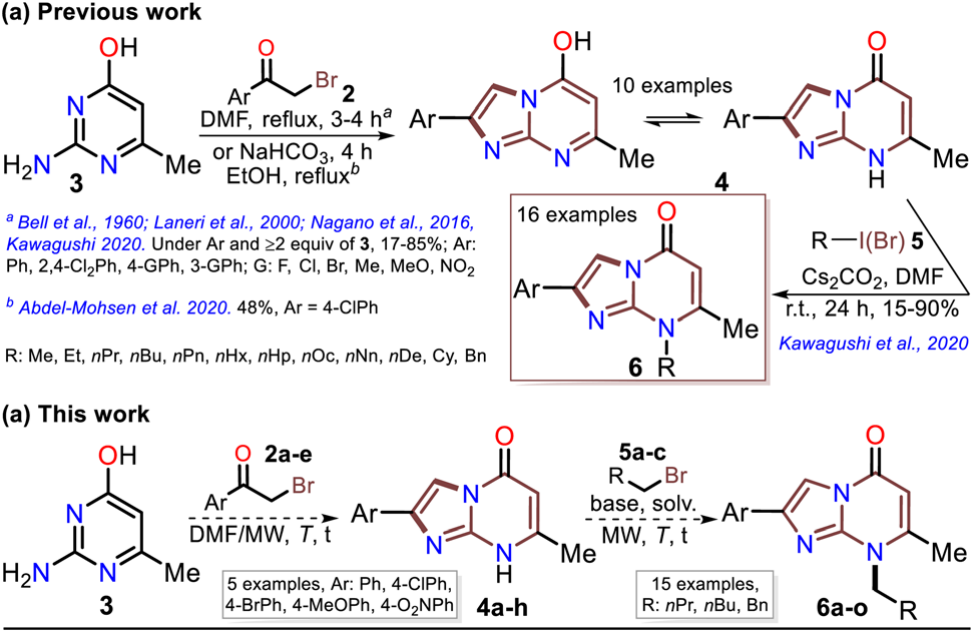
(a) Reported and (b) proposed synthesis of the *N*-alkylated IPs 4.

Although various commercial 2-aminopyrimidines are cheap, their poor solubility and high melting points limit their use in synthesis.^39–41^ For example, we could find only five reports on IP synthesis using 2-amino-6-methylpyrimidin-4(1*H*)-one (3, 6-methylisocytosine);^14,15,42–44^ particularly, 2-arylimidazo[1,2-*a*]pyrimidin-5(8*H*)-ones (4) were obtained with poor to good yields *via* the reaction of 3 (≥2 equiv.) with α-bromoketones 2 under reflux in DMF^14,42–44^ or ethanol (with NaHCO_3_ and 1 equiv. of 3).^15^ Remarkably, among the three recent works reporting crucial biological results,^14,15,44^ the best yield and more molecular diversity were reported in 2020 by Kawaguchi's group.^14^ This group also obtained the biologically active *N*-alkylated products (6) by reacting 4 (mainly from Ar = 4-ClPh) with alkyl halides (5) (2 equiv.) and an excess (4 equiv.) of cesium carbonate (Scheme 2a).^14^ In 1966, Pyl and Baufeld reported a method for obtaining an *N*-methyl-2-phenyl derivative (74% yield) using dimethyl sulfate (5 equiv.) and sodium hydroxide.^29^ Considering these findings and our interest in improving and standardizing synthetic methods under MW conditions,^36–38,45^ in this work, we used this technique to obtain 2-arylimidazo[1,2-*a*]pyrimidin-5(8*H*)-ones 4a–e and a new family of their *N*-alkylation products 6a–o (Scheme 2b). The 8-alkyl-2-arylimidazo[1,2-*a*]pyrimidinones 6a–o exhibited good solubility in organic solvents, meeting the desired expectations and incurring low cost as this approach involved cheap and easily accessible intermediates and reagents.

## Results and discussion

### Synthesis of 2-arylimidazo[1,2-*a*]pyrimidinones 4a–e

**Scheme 3:**
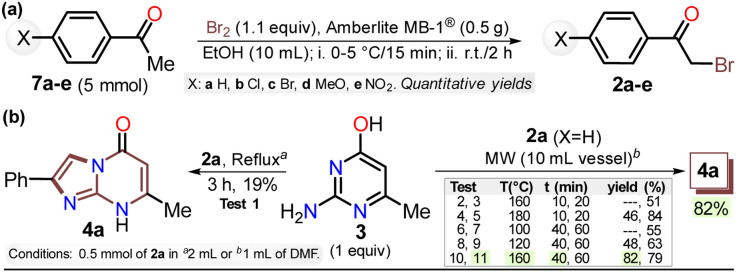
Synthesis of (a) α-bromoketones 2a–e and (b) imidazo[1,2-*a*]pyrimidine 4a.

First, α-bromoacetophenones 2a–e were prepared by known protocols using acetophenones 7a–e and bromine in polar solvents;^37,38^ Amberlite MB-1® (0.1 g mmol^−1^ of 7) was used as a solid catalyst support to obtain the best feasible results (Scheme 3a). Meshram *et al.*^46^ used NBS and Amberlyst-15® (like-reticular resin for acidic catalysis) under similar reactions, but we used bromine (Br_2_) as it is cheaper than NBS. Both are ion-exchange resins of polystyrene–divinylbenzene; however, gel-like Amberlite MB-1® has amphoteric properties and is usually used in chromatography and synthesis. Products were obtained with quantitative yields because perhaps bromine and its residues were easily encapsulated in this resin, favoring the reaction and product isolation.^47^ Then, by a standard MW-assisted route, we carried out the reaction of 2a–e with 3 (1 equiv.) to obtain 2-arylimidazo[1,2-*a*]pyrimidinones 4a–e (Schemes 3b). In this way, we reproduced the synthesis of 4a (Ar = Ph) by Laneri *et al.* (they obtained an 80% yield using 2.5 equiv. of 3)^43^ but with poor yield using 1 equiv. of 3 (Scheme 3b, test 1). However, we heated the reaction mixture under MW at 160 °C, and product formation was observed after 20 minutes with moderate yield (50%, test 3 *vs.* 2). Then, the reaction mixture was heated at 180 °C, and the reagents were consumed after 20 minutes, forming 4a with a 84% yield (test 5 *vs.* 4). With a focus on lowering the temperature, the reaction mixture was heated from 100 to 160 °C for a longer time (40 and 60 min), and the reagents were consumed after 60 minutes at 100 °C and 40 min at 160 °C, producing 4a with a 82% yield (test 6 to 11). Thus, 160 °C and 30 minutes under MW conditions were considered the optimal reaction conditions (test 11). Finally, although 4a can be obtained by refluxing in DMF, this method was revised due to poor yield and the need for an excess of 3, which is a highly insoluble reagent that decomposes under these conditions.

**Scheme 4:**
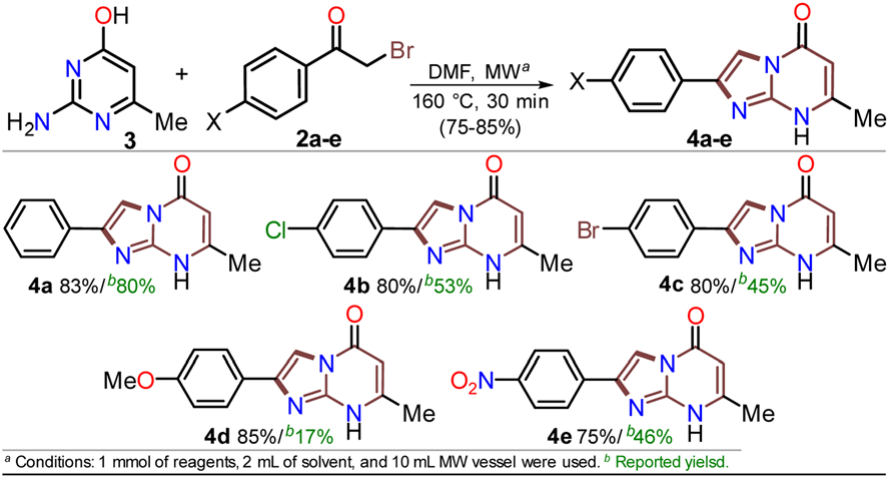
MW-assisted synthesis of the 2-arylimidazo[1,2-*a*]pyrimidin-5(8*H*)-ones 4a–e.

The reaction scope was examined using an equimolar mixture of 6-methylisocytosine (3) and α-bromoacetophenones 2a–e (1 mmol) under MW heating at 160 °C for 20 minutes. This reaction yielded 2-arylimidazo[1,2-*a*]pyrimidin-5(8*H*)-ones 4a–e with high yields as white-yellow solids that had high melting points (Scheme 4).

Notably, pure products were obtained when the reaction mixture was treated with water (3 mL), the deposited solid was collected by simple filtration, washed with cold ethanol (2 × 2 mL), and placed under a high vacuum for one hour at 60 °C. In addition, almost no loss of reaction efficacy was observed with the α-bromoketones tested, evidencing that the electronic nature of the substituent in the phenyl ring had little effect on the reactivity of 2a–e. However, the lowest yield was observed for 4e as the reaction mixture turned dark when the nitro-substituted substrate 2e was used, which may be due to its high polarity. These results suggest the feasibility of improving the synthetic method of 4a–e using sustainable protocols (*i.e.*, ecological, social, and economic scopes) compared with other reported synthetic methods.

### Functionalization of the 2-arylimidazo[1,2-*a*]pyrimidinones 4a–e

Although the solubility of the 2-arylimidazo[1,2-*a*]pyrimidin-5(8*H*)-ones 4a–e in organic solvents was better than that of 6-methylisocytosine (3), it was still too low to favor its reactivity in further synthesis. Thus, with 4a–e in hand, we envisaged that their *N*-alkylation reaction with alkyl bromines 5a–c (1 equiv.) under microwave irradiation (15 min) could be used to prepare 8-alkyl-2-arylimidazo[1,2-*a*]pyrimidinones 6a–o. We optimized this reaction by synthesizing the *N*-propyl derivative 6a using 4a and *n*-propyl bromide (5a) as the model reagents (Table 1). The effect of cesium in promoting MW-assisted reactions^48^ and its value in preparing products that are highly soluble in organic solvents, such as 6a–o, are well-known;^14^ however, we wanted to implement an efficient protocol by involving a base cheaper than Cs_2_CO_3_ (*i.e.*, NaH, *t*BuOK, Na_2_CO_3_, or K_2_CO_3_) in lower quantity (1 equiv.) and using the minimum amount of aprotic solvent (*i.e.*, MeCN, DMSO, or DMF). In this optimization experiment, the best results were obtained when the reaction was carried out in dry DMF at 150 °C using potassium carbonate as the base (entry 7 *vs.* 1 to 6).

**Table 1:** Optimization of the synthesis of the 2-phenyl-8-propyl derivative 6a^a^

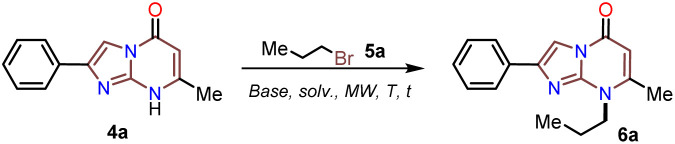					
Entry	Solvent	Base	*T* (°C)	*t* (min)	Yield (%)
1	—	NaH	150	15	NR
2		*t*BuOK			NR
3		Na_2_CO_3_			Traces
4		K_2_CO_3_			16
5	MeCN				33
6	DMSO				Traces
7	DMF				55
8^b^		K_2_CO_3_^b^			70
9^b^			130		75
10^b^			100		83
11^b^			60		43
12^b^				60	77
13^c^		K_2_CO_3_^c^	100	15	85
14^c^				60	80
15^c^	MeCN				67

a Reactions conditions: 4a (0.25 mmol) and 5a/base (1 equiv.). Experiments performed in 10 mL sealed tubes under MW in 0.5 mL of the solvent. NR = no reaction.

b 5a/base (2 equiv.).

c 5a/base (1.5 equiv.).

**Scheme 5:**
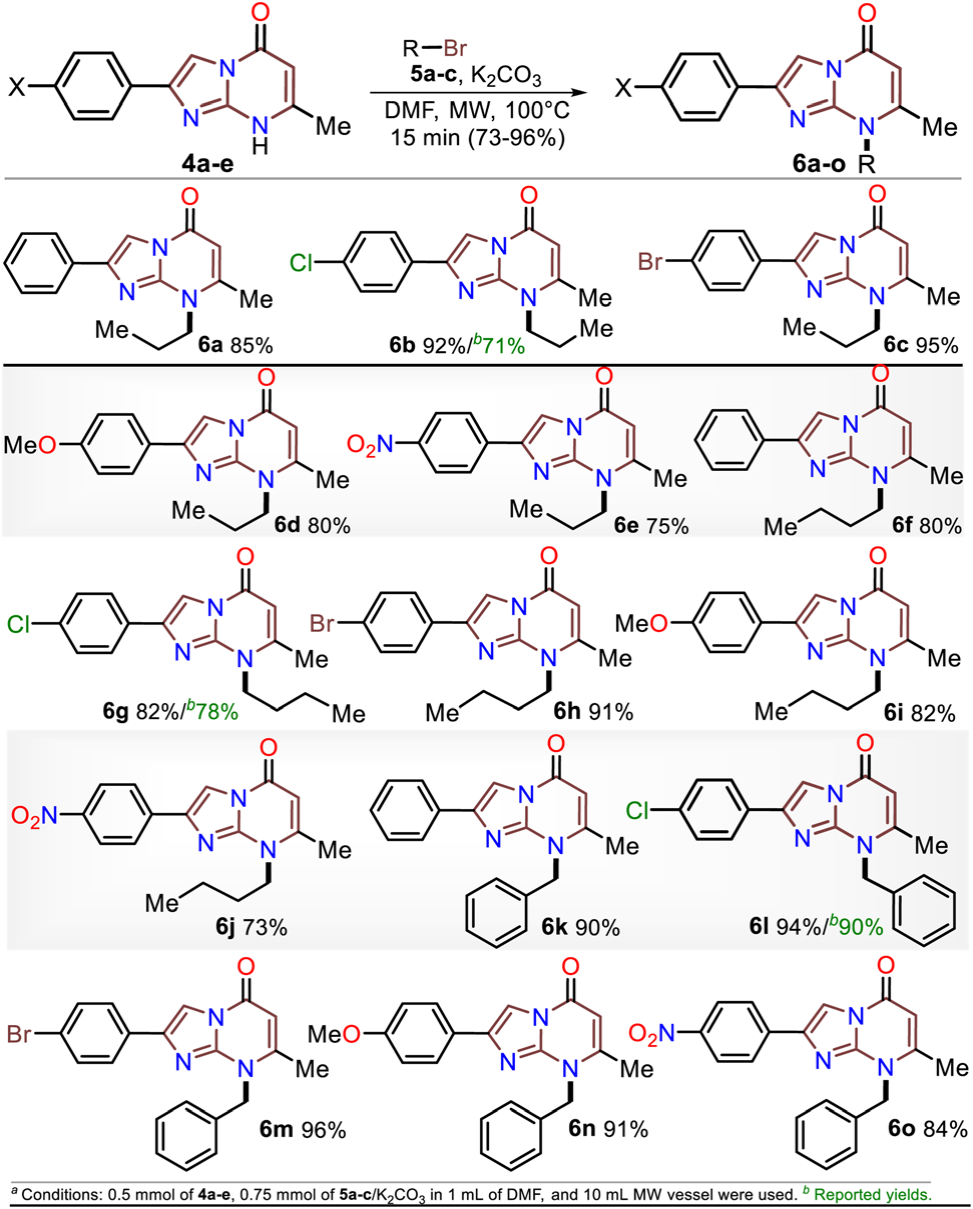
MW-assisted synthesis of the 8-alkyl-2-arylimidazo[1,2-*a*]pyrimidinones 6a–o.

Subsequently, the substrate 5a and the potassium carbonate equivalents were doubled, the temperature was reduced, and the reaction time was increased (entries 8 to 12, Table 1); in these experiments, the best results were obtained when the reaction was carried at 100 °C for 15 minutes (entry 10). Ultimately, the equivalent amounts of the substrate and base were reduced to 1.5, which afforded the optimal results at 100 °C for 15 minutes in DMF as the solvent (entry 13). However, good results were obtained when the reaction was carried out at 60 °C for 1 hour and even in acetonitrile as the solvent at 100 °C (entries 12 and 15). Therefore, with the optimal conditions in hand to form 6a, we investigated the scope of the *N*-alkylation reaction for 4a–e using alkyl bromides 5a–c (Scheme 5). In general, the reaction showed excellent tolerance of reagents, resulting in the desired products 4a–o as colorless solids with outstanding yields (>73%). In addition, the conditions used for obtaining the precursors and products are better than those for reported IPs (*i.e.*, 4a–e and 6b, 6g, and 6l).

**Scheme 6:**
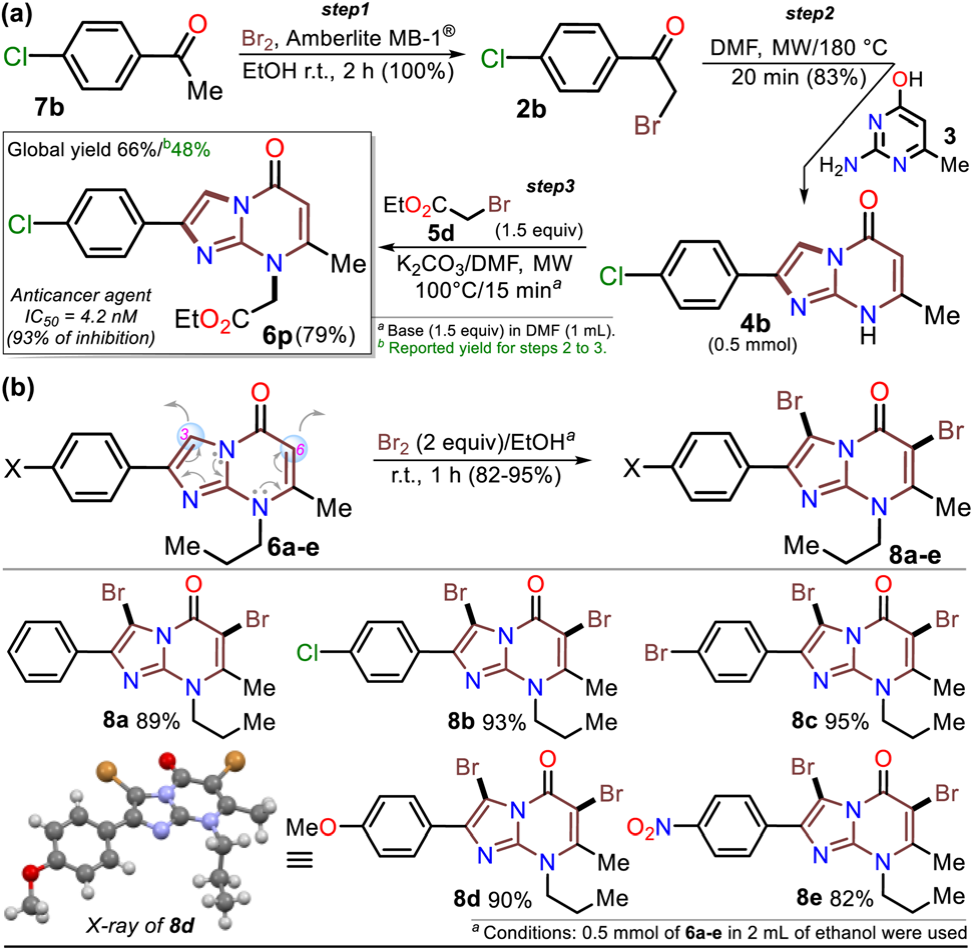
Synthesis of (a) inhibitor 6p and (b) the 3,6-dibromo derivatives 8a–e.

Once the IPs 6a–o were obtained, the practical utility of this approach in medical and synthetic chemistry settings was explored. Specifically, the preparation of the anticancer agent ethyl 2-(4-chlorophenyl)-7-methyl-5-oxoimidazo[1,2-*a*]pyrimidine-8-acetate (6p) and the reactivity of the rings in 6a–e in the bromination reaction were evaluated (Scheme 6). Notably, Kawaguchi *et al.*^14^ have identified ester 6p (Hit A) as a potent and selective autotaxin (ATX) inhibitor that rescues the ATX-induced cardia bifida phenotype in zebrafish embryos. They obtained 6b and other *N*-alkyl derivatives by an approach similar to that used for 6a–o (see Scheme 2a); however, our standard MW-assisted approach proved more efficient in various aspects (Schemes 4–6a).

Furthermore, the reactivity of 6a–e was successfully evaluated by a simple bromination reaction. The imidazo[1,5-*a*]pyrimidine ring can react with electrophiles at two places, but the π-excessive nature of imidazole favors position 3 over 6 in the pyrimidine core. Most electrophilic aromatic substitution (EAS) reactions lead to 3-substituted products,^18–21,43^ but the alkyl group in 6a–e played a crucial role in their reactivity. Indeed, 3,6-dibrominated IPs 8a–e were easily obtained, while directed regioselectivity could not be achieved despite strict control of the reaction conditions. For example, in the NMR and HRMS analyses of the reaction crude of 6b with 1 equiv. of bromine at 0 °C for 10 min, a mixture of compounds was observed (*i.e.*, 8b > 6-Br6b > 3-Br6b > 6b), evidencing the high reactivity of 6b and its preference to form the dibrominated product 8b. As a result, by using 2 equiv. of bromine for 1 hour at room temperature, compounds 8a–e were efficiently obtained (Scheme 6b). This type of compound would be useful in Pd-catalyzed C–C cross-coupling reactions,^18–21^ and the reaction approach may spearhead future syntheses based on EAS reactions of the imidazo[1,2-*a*]pyrimidine ring.

The structures of the compounds obtained were elucidated by HRMS analysis and ^1^H and ^13^C NMR spectroscopy, including some two-dimensional methods (see experimental processes, characterization data, and NMR spectra in ESI†). Gratifyingly, recrystallization of the 3,6-dibromoimidazo[1,5-*a*]pyrimidine 8b from a chloroform–methanol mixture (1 : 1 v/v) afforded crystals of suitable size and quality for single-crystal X-ray diffraction analysis (Scheme 6b).^49^

**Fig. 2:**
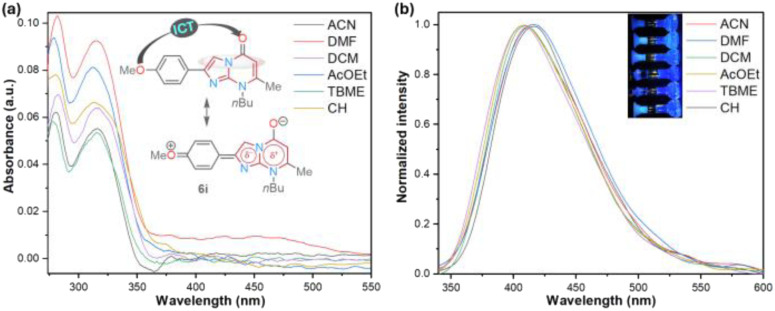
(a) UV-vis absorption and (b) fluorescence emission (normalized, *λ*_ex_ = 315 nm) spectra of 6i in different solvents (6.6 μM) at 20 °C. The inset shows the photograph of 200 μM solutions.

On the other hand, we performed a preliminary photophysical study of the 2-(4-methoxyphenyl) derivative 6i to establish the scope of the imidazo[1,5-*a*]pyrimidine heterocyclic core as an organic fluorophore due to its limited exploration^9–11^ and our wide interest in this field (Fig. 2 and Table 2).^7,8^ This study was conducted to classify compounds 6a–o as strategic intermediates of novel functional fluorophores due to their high synthetic viability. In particular, 6i was chosen for the research since its aryl group (*i.e.*, 4-MeOPh) has revealed exceptional photophysical results in a similar 5 : 6 heterocyclic core, namely the pyrazolo[1,5-*a*]pyrimidine ring, by intramolecular charge transfer (ICT) photophysical phenomena.^9–11^

**Table 2:** Photophysical data of the 8-butylimidazo[1,2-*a*]pyrimidinone 6i^a^

Solvent	*λ*_abs_ (nm)	*ε* (M^−1^ cm^−1^)	*λ*_em_ (nm)	SS (cm^−1^)	*ϕ*_F_	*B*(*ε* × *ϕ*_F_)
ACN	281, 315	8540	412	7474	0.113	966
DMF	282, 317	14 094	420	7736	0.070	993
DCM	283, 314	10 054	404	7095	0.085	854
AcOEt	279, 314	12 578	414	7693	0.182	2286
TBME	279, 314	8170	406	7217	0.087	713
CH	281, 308	10 505	420	8658	0.040	421

a Quantum yield (*ϕ*_F_) values were determined using Prodan as the standard. Absorbance (ab), fluorescence emission (em), molar absorption coefficient (*ε*), Stokes shift (SS), and calculated brightness (*B*) data are shown.

The UV-vis and fluorescence emission spectra of 6i were achieved in six solvents with different polarities, including cyclohexene (CH), *t*-butyl methyl ether (TBME), dichloromethane (DCM), ethyl acetate (AcOEt), *N*,*N*-dimethylformamide (DMF), and acetonitrile (MeCN); the results are shown in Fig. 2 and Table 2. Cyclohexene was used as the lowest polarity solvent since 6i is highly insoluble in alkanes (*e.g.*, cyclohexane). The absorption spectra of 6i displayed two typical bands, one at 280 nm attributed to the π → π* transitions and the other at around 315 nm due to the attenuation So → ICT transitions (*i.e.*, MeO → C=O), which are attenuated by the dipolar nature of the fused ring of 6i. As a result, the polarity of the microenvironment moderately influenced the photophysical properties of 6i (Fig. 2a). Fluorophore 6i displayed large Stokes shifts (7474–8658 cm^−1^) without a specific solvatofluorochromic shift in the six evaluated solvents; however, 6i exhibited appreciable fluorescence intensity (*ϕ*_F_ of up to 0.18), and the highest fluorescence quantum yield (*ϕ*_F_) and brightness (*B* = *ε* × *ϕ*_F_ = 2286) values were achieved in a medium-polarity solvent AcOEt (Fig. 2b and Table 2). From these results, we can establish that the heterocyclic core of imidazo[1,2-*a*]pyrimidine is a promising functional fluorophore for application in detection chemistry, diagnostic bioimaging, and photosensitizers.^2,9–11^

## Conclusions

In summary, 2-arylimidazo[1,2-*a*]pyrimidin-5(8*H*)-ones 4a–e and their *N*-alkylation 6a–o and 3,6-debromination 6a–e products were successfully synthesized with high yields through microwave-assisted reactions using easily accessible and inexpensive reagents. All the obtained compounds were characterized by HRMS spectroscopy and NMR analysis, and the structure of the final product (8d) was confirmed by single-crystal X-ray diffraction analysis. The synthetic utility of the alkylation reaction was further proven by synthesizing the anticancer drug 6p (Hit A). Notably, 6a–o were more soluble in organic solvents than their precursors 4a–e, thus expanding the applicability of this type of heterocyclic compound. Indeed, this was verified by the straightforward synthesis of 8a–e; however, it is worth studying the reactivity of the N-heterocyclic ring further to achieve more regioselectivity of the substituents on its periphery. In addition to the synthetic and biological applications of 6a–p, the remarkable photophysical properties exhibited by 6i highlight the high potential of these compounds as modular fluorophores.

## Data availability

This paper is original work that has not been previously submitted for publication to another journal and is not currently considered elsewhere. Additionally, we have no conflicts of interest to report with this submission, and all authors have seen, revised, and approved the submitted manuscript. This manuscript has experimental data with suitable ESI† available for web publication (NMR and HRSM spectra and crystallographic details CCDC: 8d2358854).

## Conflicts of interest

The authors declare no competing financial interest.

## Author contributions

The individuals listed as authors have contributed to developing this manuscript, and no other person was involved. The authors' contributions included: D. C., C. C., and J.-C. C., carried out experiments and literature review, and J. P. the composition of the original draft, supervision, and sources. All authors have read and agreed to the published version of this manuscript.

## Supplementary Material

RA-014-D4RA03948C-s001

RA-014-D4RA03948C-s002
