# Supplementary material for: Microwave-assisted synthesis and functionalization of 2-arylimidazo[1,2-a]pyrimidin-5(8H)-ones
Source: RSC Adv. 2024 Jul 15;14(31):22368–73. doi: 10.1039/d4ra03948c (PMC11247617; doi:10.1039/d4ra03948c)
Supplement: RA-014-D4RA03948C-s001 [file RA-014-D4RA03948C-s001.pdf]

Electronic Supplementary Material (ESI) for RSC Advances.
This journal is © The Royal Society of Chemistry 2024

Electronic supplementary information (ESI)

Microwave-assisted synthesis and functionalization of 2-arylimidazo[1,2-*a*]pyrimidin-5(8*H*)-ones

Delascar Camargo,[a] Carlos Cifuentes,[a] Juan-Carlos Castillo,[a,b] and Jaime Portilla*[a]

[a] Bioorganic Compounds Research Group, Department of Chemistry, Universidad de Los Andes, Carrera 1 No. 18A-10, Bogotá 111711, Colombia

[b] Escuela de Ciencias Químicas, Universidad Pedagógica y Tecnológica de Colombia, Avenida Central del Norte 39-115, Tunja, Colombia

*E-mail: jportill@uniandes.edu.co; Tel: +57 1 3394949. Ext. 2080

Contents

1. **Overview of substrates and products numbering.. S2**
2. **Experimental details... S3**
3. **Copies of NMR spectra...S12**
4. **Photophysical and crystallographic details...S41**
5. **References...S41**

1. Overview of substrates and products numbering

(a) Substrates/reagents. 6-methylisocytosine (**3**), acetophenones **7a-e**, alkyl bromides **5a-c**, bromine, and α-bromoacetophenones **2a-e**.

Compounds purchased in our laboratory/price*

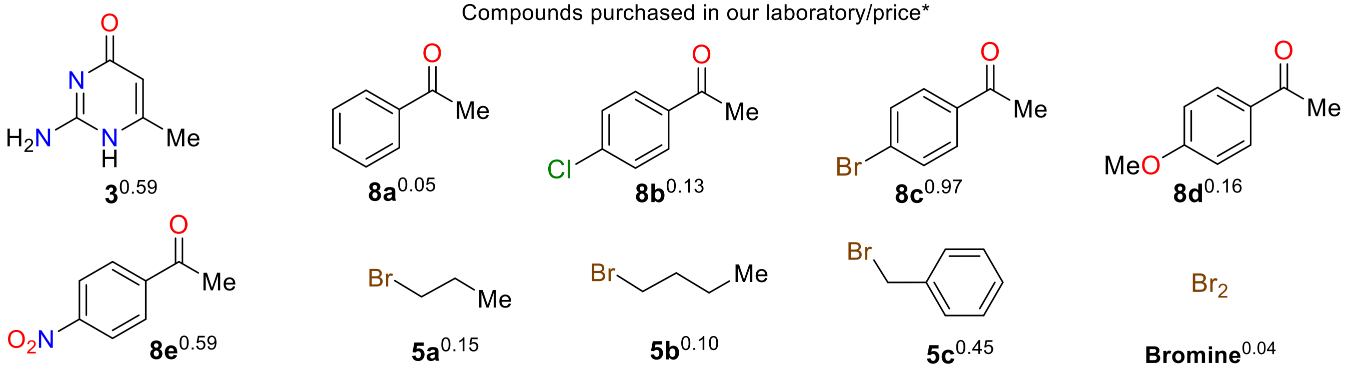

Compound synthesized in our laboratory*

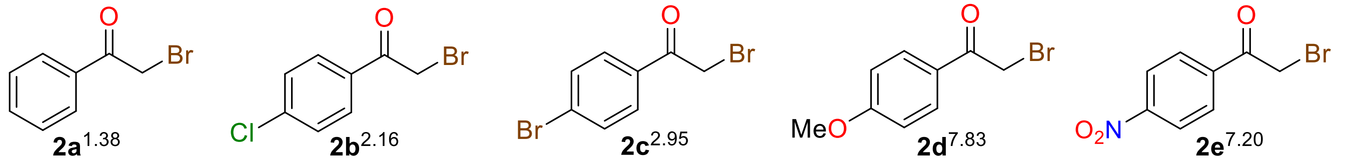

*Approximate prices in USD per gram or mililitre were consulted via MERCK or Fisher Scientific, on May 9 2024. From presentations for 0.1 to 1 Kg or 25g for **2a-e**.

(b) 2-Aryl-7-methylimidazo[1,2-*a*]pyrimidin-5(8*H*)-ones 4a-e

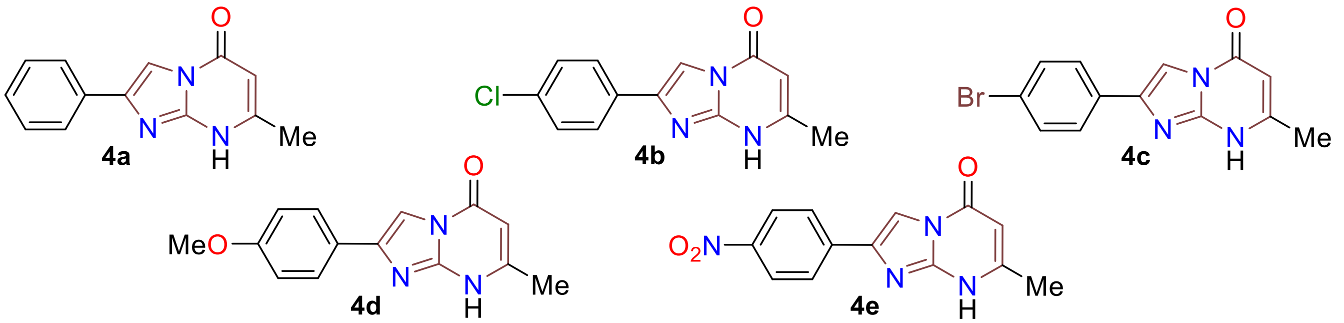

(c) 2-Aryl-7-methylimidazo[1,2-*a*]pyrimidin-5(8*H*)-ones 4a-e

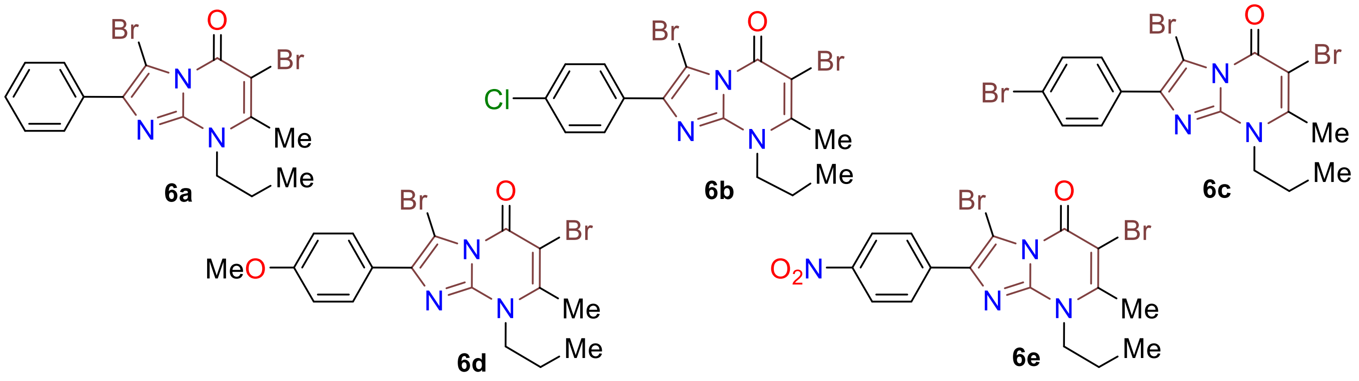

Fig. S1 Structures of (a) commercial (top) and synthetics (bottom) substrate/reagents, (b) 2-aryl-7-methylimidazo[1,5-*a*]pyrimidones **4a-e**, and (c) 2-aryl-3,6-dibromo-7-methyl-8-propylimidazo[1,5-*a*]pyrimidones **4a-e**

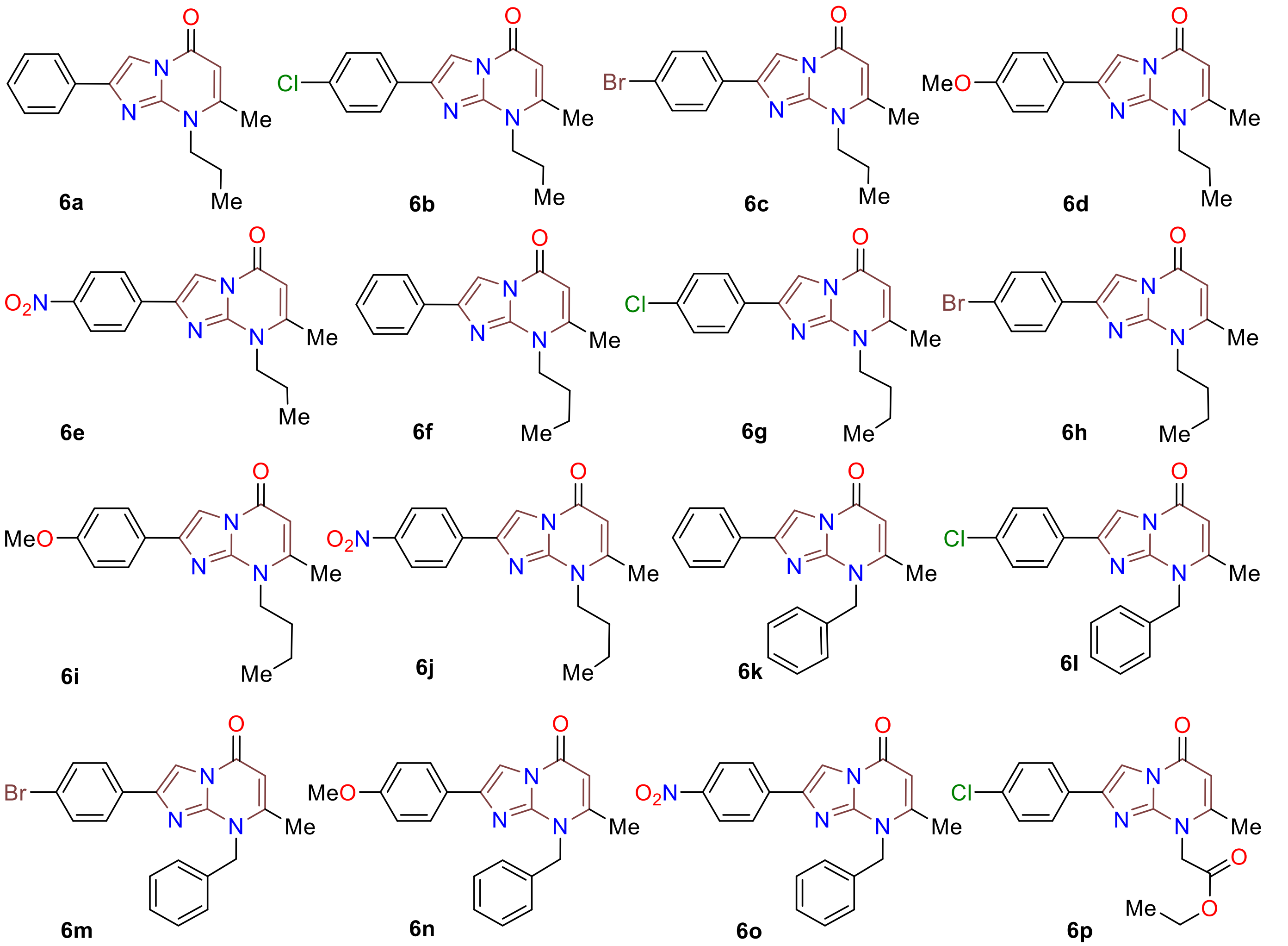

Fig. S2 Structure of N-alkylated 2-aryl-7-methylimidazo[1,5-*a*]pyrimidones **6a-p**

2. Experimental details

2.1. Reagents and materials

Reagents were acquired from commercial sources, used without further purification, and weighed and handled in air at room temperature (r.t.). Reactions were monitored by thin-layer chromatography (TLC) and visualized by a UV lamp (254/365 nm). Silica gel (230-400 mesh) for flash chromatography was used. Reactions under MW irradiation were carried out in a sealed reaction vessel (10.0 mL, max pressure = 300 psi) containing a Teflon-coated stir bar (obtained from CEM) and were performed in a CEM Discover SP-focused MW (ν = 2.45 GHz) reactor equipped with a built-in pressure measurement sensor and a vertically focused IR temperature sensor. Controlled temperature, power, and time settings were used.

NMR spectra were recorded at 400 MHz (^{1}H) and 101 MHz (^{13}C) at 298 K, and data were recorded in $CDCl_3$ (7.26/77.0 ppm) or DMSO (2.50/39.5 ppm) using the residual nondeuterated signal for ^{1}H and the deuterated solvent signal for ^{13}C NMR as internal standards. Chemical shifts (δ) are given in parts per million (ppm) and coupling constants (*J*) in Hertz (Hz). The multiplicity abbreviations involve s = singlet, d = doublet, t = triplet, q = quartet, and m = multiplet. Melting points were measured by a capillary melting point device, and data were uncorrected. High-resolution mass spectra (HRMS) were recorded by a Q-TOF spectrometer using electrospray ionization (ESI). Crystallographic data were recorded on a diffractometer using graphite-monochromated Mo Kα radiation (λ = 0.71073 Å). Structures were solved by direct methods in SHELXS-97.[1]

2.2. General procedure and characterization data

2.2.1. Synthesis of α-bromoacetophenones 2a-e.

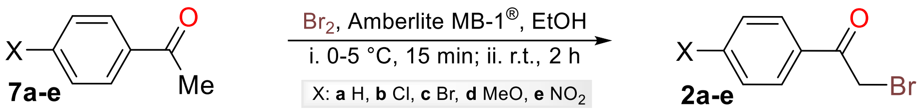

To a mixture of acetophenone **7a-e** (5.00 mmol) and Amberlite MB-1® (500 mg) in ethanol (10 mL) cooled by an ice bath, bromine (5.5 mmol to 99.5%, 883 mg) was added slowly, and it stirred first for 15 min and then more time at room temperature. After the completion of the reaction in around 2 h (monitored by TLC), it was filtered off and washed with ethanol (2 × 2.5 mL). Then, water was added (5 mL) to the filtered solution, and the mixture was extracted with DCM (3 × 30 mL). The organic phase was dried over anhydrous $MgSO_4$ and concentrated under vacuum giving **2a-d** as white solids, **2e** is a yellow solid. NMR data for **2a-e** matched the reported data in the literature.[2,3]

2-Bromoacetophenone (2a): 965 mg (97%), mp: 49–50 °C (Lit.[2] 43–45 °C). ^{1}H NMR (400 MHz, $CDCl_3$): δ = 4.46 (s, 2H), 7.50 (t, *J* = 7.6 Hz, 2H), 7.61 (t, *J* = 7.6 Hz, 1H), 7.99 (d, J = 8.4 Hz, 2H) ppm.

2-Bromo-4'-chloroacetophenone (2b): 1167 mg (quantitative), mp: 96–97 °C (Lit.[2] 96–98 °C). ^{1}H NMR (400 MHz, $CDCl_3$): δ = 4.41 (s, 2H), 7.49 (d, *J* = 8.6 Hz, 2H), 7.94 (d, *J* = 8.6 Hz, 2H) ppm.

2,4'-Dibromoacetophenone (2c): 1.39 g (quantitative), Mp: 107–108 °C (Lit.[4] 106–108 °C). ^{1}H NMR (400 MHz, $CDCl_3$): δ = 4.46 (s, 2H), 7.64 (d, *J* = 8.7 Hz, 2H), 7.86 (d, *J* = 8.7 Hz, 2H) ppm.

2-Bromo-4'-methoxyacetophenone (2d): 955 mg (96%), Mp: 70–71 °C (Lit.[2] 70–72 °C). ^{1}H NMR (400 MHz, $CDCl_3$): δ = 4.40 (s, 2H), 6.65 (d, *J* = 8.8 Hz, 2H), 7.86 (d, *J* = 8.8 Hz, 2H) ppm.

2-Bromo-4'-nitroacetophenone (2e): 1.22 g (quantitative), Mp: 90–91 °C (Lit.[4] 91–93 °C). ^{1}H NMR (400 MHz, DMSO): δ = 4.50 (s, 2H), 7.91 (t, *J* = 8.2 Hz, 2H), 8.21 (t, *J* = 8.1 Hz, 2H) ppm.

2.2.2. Synthesis of 2-aryl-7-methylimidazo[1,2-a]pyrimidin-5(8H)-ones 4a-e.

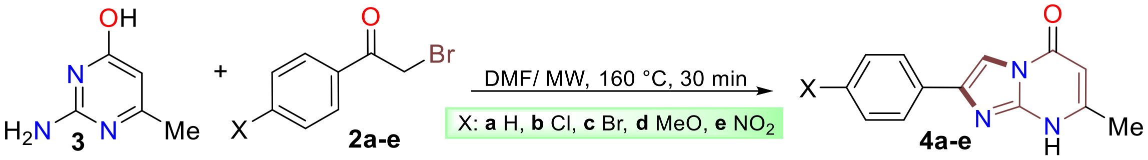

An equimolar mixture of 2-amino-4-hydroxy-6-methylpyrimidine (**3**, 1.0 mmol, 125 mg) and the appropriate α-bromoketone **2a-e** in DMF (2.0 mL) was subjected to microwave irradiation at 160 °C (170 W programmed but ~20% of this was necessary and constant. Temperature monitored by an IR sensor) for 30 min in a sealed tube containing a Teflon-coated magnetic stirring bar. The resulting reaction mixture was cooled to 55 °C by airflow, and the precipitated product formed upon the addition of cold water (3.0 mL) was filtered, washed with cold ethanol (2 × 2 mL), and dried under a high vacuum for one hour at 60 °C to give the pure products **4a-e** as yellowish-white solids. Compounds **4a-e** were characterized by comparing their data with information available in the literature, *e.g.*, NMR data for **4a-e** matched previously reported data.[5–7]

7-Methyl-2-phenylimidazo[1,2-*a*]pyrimidin-5(8*H*)-one (4a) was obtained in 83% yield (187 mg) from 2-bromoacetophenone (**2a**, 198 mg, 1.00 mmol). Mp > 300 °C (amorphous, Lit.[5] 315–317 °C). ^{1}H NMR (400 MHz, DMSO-d_6): δ = 2.29 (s, 3H), 5.64 (s, 1H), 7.31 (t, J = 7.6 Hz, 1H), 7.42 (t, J = 7.7 Hz, 2H), 7.90 (d, J = 7.7 Hz, 2H), 8.05 (s, 1H), 12.85 (br s, 1H) ppm. ^{13}C{^{1}H} NMR (101 MHz, DMSO-d_6): δ = 19.8 (CH_3), 95.2 (CH), 103.5 (CH), 125.2 (CH), 127.8 (CH), 128.6 (CH), 132.1 (C), 137.9 (C), 144.1 (C), 153.5 (C), 157.0 (C) ppm.

2-(4-Chlorophenyl)-7-methylimidazo[1,2-*a*]pyrimidin-5(8*H*)-one (4b) was obtained in 80% yield (208 mg) from 2-bromo-4'-chloroacetophenone (**2b**, 236 mg, 1.01 mmol). Mp > 300 °C (amorphous, Lit.[6] > 300 °C) [2]. ^{1}H NMR (400 MHz, DMSO-d_6): δ = 2.29 (s, 3H), 5.63 (s, 1H), 7.45 (d, J = 8.6 Hz, 2H), 7.91 (d, J = 8.6 Hz, 2H), 8.10 (s, 1H), 12.82 (br s, 1H) ppm. ^{13}C{^{1}H} NMR (101 MHz, DMSO-d_6): δ = 19.3 (CH_3), 95.0 (CH), 104.2 (CH), 126.8 (CH), 128.6 (CH), 131.6 (C), 132.1 (C), 137.5 (C), 143.8 (C), 151.7 (C), 157.0 (C) ppm.

2-(4-Bromophenyl)-7-methylimidazo[1,2-*a*]pyrimidin-5(8*H*)-one (4c) was obtained in 80% yield (243 mg) from 2-bromo-4'-bromoacetophenone (**2c**, 278 mg, 1.00 mmol). Mp > 300 °C (amorphous, Lit.[7] > 300 °C) [3]. ^{1}H NMR (400 MHz, DMSO-d_6): δ = 2.29 (s, 3H), 5.63 (s, 1H), 7.59 (d, J = 7.7 Hz, 2H), 7.85 (d, J = 7.7 Hz, 2H), 8.11 (s, 1H), 12.82 (br s, 1H) ppm. ^{13}C{1H} NMR (101 MHz, DMSO-d_6): δ = 19.3 (CH_3), 95.0 (CH), 104.2 (CH), 120.6 (C), 127.1 (CH), 131.5 (CH), 131.7 (C), 137.3 (C), 143.7 (C), 152.2 (C), 157.0 (C) ppm.

2-(4-Methoxyphenyl)-7-methylimidazo[1,2-*a*]pyrimidin-5(8*H*)-one (4d) was obtained in 85% yield (217 mg) from 2-bromo-4'-methoxyacetophenone (**2d**, 234 mg, 1.02 mmol). Mp > 300 °C (amorphous, Lit.[7] > 300 °C). ^{1}H NMR (400 MHz, DMSO-d_6): δ = 2.29 (s, 3H), 3.79 (s, 3H), 5.64 (s, 1H), 6.99 (d, J = 8.7 Hz, 2H), 7.83 (d, J = 8.7 Hz, 2H), 7.93 (s, 1H), 12.74 (br s, 1H) ppm. ^{13}C{^{1}H} NMR (101 MHz, DMSO-d_6): δ = 20.1 (CH_3), 55.1 (CH_3), 95.4 (CH), 102.1 (CH), 114.2 (CH), 124.2 (C), 126.6 (CH), 137.0 (C), 144.2 (C), 153.9 (C), 157.0 (C), 159.1 (C) ppm.

2-(4-Nitrophenyl)-7-methylimidazo[1,2-*a*]pyrimidin-5(8*H*)-one (4e) was obtained in 75% yield (217 mg) from 2-bromo-4'-nitroacetophenone (**2e**, 249 mg, 1.02 mmol). Mp 281–282 °C (amorphous, Lit.[7] > 300 °C). ^{1}H NMR (400 MHz, DMSO-d_6): δ = 2.30 (s, 3H), 5.63 (s, 1H), 8.12 (d, J = 8.8 Hz, 2H), 8.21 (d, J = 8.8 Hz, 2H), 8.30 (s, 1H), 12.89 (br s, 1H) ppm. ^{13}C{^{1}H} NMR (101 MHz, DMSO-d_6): δ = 19.0 (CH3), 94.9 (CH), 106.9 (CH), 123.9 (CH), 125.8 (CH), 137.6 (C), 139.6 (C), 143.8 (C), 146.2 (C), 152.2 (C), 157.0 (C) ppm.

2.2.3. Synthesis of 8-alkyk-2-aryl-7-methylimidazo[1,2-a]pyrimidin-5(8H)-ones 6a-p.

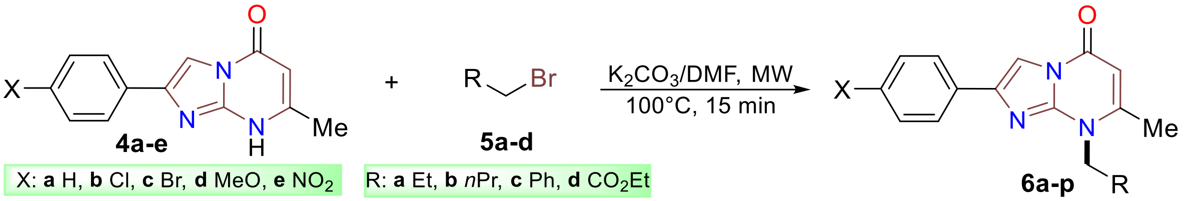

A mixture of 2-aryl-7-methylimidazo[1,2-*a*]pyrimidin-5(8*H*)-one **4a-e** (0.50 mmol), K_2CO_3 (104 mg, 0.75 mmol), and alkyl bromide **5a-d** (0.75 mmol) in DMF (1.0 mL) was subjected to microwave irradiation at 100 °C (110 W programmed but ~15% of this was necessary and constant. Temperature monitored by an IR sensor) for 15 min in a sealed tube containing a Teflon-coated magnetic stirring bar. The resulting reaction mixture was cooled to 55 °C by airflow, and the precipitated product formed after adding cold water (2.0 mL) was subjected to extraction with dichloromethane (3 × 10 mL). The organic phase was dried over anhydrous $MgSO_4$, filtered, and concentrated under vacuum giving **6a-p** as white solids. 2-(4-Chlorophenyl) derivatives were characterized by comparing their data with information available in the literature, that is, NMR data for **6b** (*n*Pr), **6g** (*n*Bu), **6l** (Bn), and **6p** (ester) matched previously reported data.[7]

7-Methyl-2-phenyl-8-propylimidazo[1,2-*a*]pyrimidin-5(8*H*)-one (6a) was obtained in 85% yield (116 mg) from 7-methyl-2-phenylimidazo[1,2-*a*]pyrimidin-5(8*H*)-one (**4a**, 115 mg, 0.51 mmol) and 1-bromopropane (**5a**, 70 µL, 0.77 mmol). Mp 145–146 °C (amorphous). ^{1}H NMR (400 MHz, $CDCl_3$): δ = 1.05 (t, J = 7.2 Hz, 3H), 1.84-1.94 (m, 2H), 2.41 (s, 3H), 4.25 (t, J = 7.7 Hz, 2H), 5.67 (s, 1H), 7.30 (t, J = 7.5 Hz, 1H), 7.40 (t, J = 7.5 Hz, 2H), 7.86 (s, 1H), 7.88 (d, J = 8.1 Hz, 2H) ppm. ^{13}C{^{1}H} NMR (101 MHz, $CDCl_3$): δ = 11.1 (CH_3), 19.2 (CH_3), 21.9 (CH_2), 48.3 (CH_2), 97.7 (CH), 104.5 (CH), 125.6 (CH), 127.8 (CH), 128.6 (CH), 133.1 (C), 141.2 (C), 143.7 (C), 150.0 (C), 157.1 (C) ppm. HRMS (ESI+): calcd for $C_{16}H_{18}N_3O^+$, 268.1444 [M + H]$^+$; found, 268.1450.

2-(4-Chlorophenyl)-7-methyl-8-propylimidazo[1,2-*a*]pyrimidin-5(8*H*)-one (6b) was obtained in 92% yield (139 mg) from 2-(4-chlorophenyl)-7-methylimidazo[1,2-*a*]pyrimidin-5(8*H*)-one (**4b**, 130 mg, 0.50 mmol) and 1-bromopropane (**5a**, 70 µL, 0.77 mmol). Mp 159–160 °C (amorphous). ^{1}H NMR (400 MHz, $CDCl_3$): δ = 1.03 (t, J = 7.5 Hz, 3H), 1.84-1.91 (m, 2H), 2.40 (s, 3H), 4.22 (t, J = 7.8 Hz, 2H), 5.65 (s, 1H), 7.34 (d, J = 8.6 Hz, 2H), 7.78 (d, J = 8.6 Hz, 2H), 7.80 (s, 1H) ppm. ^{13}C{^{1}H} NMR (101 MHz, $CDCl_3$): δ = 11.1 (CH_3), 19.1 (CH_3), 21.9 (CH_2), 48.3 (CH_2), 97.7 (CH), 104.6 (CH), 126.8 (CH), 128.7 (CH), 131.7 (C), 133.4 (C), 140.2 (C), 143.8 (C), 150.1 (C), 156.9 (C) ppm. HRMS (ESI+): calcd for $C_{16}H_{17}{}^{35}ClN_3O^+$, 302.1055 [M + H]$^+$; found, 302.1045.[7]

2-(4-Bromophenyl)-7-methyl-8-propylimidazo[1,2-*a*]pyrimidin-5(8*H*)-one (6c) was obtained in 95% yield (164 mg) from 2-(4-bromophenyl)-7-methylimidazo[1,2-*a*]pyrimidin-5(8*H*)-one (**4c**, 152 mg, 0.50 mmol) and 1-bromopropane (**5a**, 70 µL, 0.77 mmol). Mp 154–155 °C (amorphous). ^{1}H NMR (400 MHz, $CDCl_3$): δ = 1.05 (t, J = 7.4 Hz, 3H), 1.86-1.96 (m, 2H), 2.43 (s, 3H), 4.25 (t, J = 7.5 Hz, 2H), 5.68 (s, 1H), 7.52 (d, J = 8.4 Hz, 2H), 7.74 (d, J = 8.4 Hz, 2H), 7.84 (br s, 1H) ppm. ^{13}C{^{1}H} NMR (101 MHz, $CDCl_3$): δ = 11.1 (CH_3), 19.2 (CH_3), 22.0 (CH_2), 48.4 (CH_2), 97.8 (CH), 104.8 (CH), 121.6 (C), 127.2 (CH), 131.7 (CH), 132.2 (C), 140.3 (C), 143.9 (C), 150.1 (C), 157.0 (C) ppm. HRMS (ESI+): calcd for $C_{16}H_{17}{}^{79}BrN_3O^+$, 346.0550 [M + H]$^+$; found, 346.0538.

2-(4-Methoxyphenyl)-7-methyl-8-propylimidazo[1,2-*a*]pyrimidin-5(8*H*)-one (6d) was obtained in 80% yield (119 mg) from 2-(4-methoxyphenyl)-7-methylimidazo[1,2-*a*]pyrimidin-5(8*H*)-one (**4d**, 128 mg, 0.50 mmol) and 1-bromopropane (**5a**, 70 µL, 0.77 mmol). Mp 114–115 °C (amorphous). ^{1}H NMR (400 MHz, $CDCl_3$): δ = 1.06 (t, J = 7.3 Hz, 3H), 1.87-1.95 (m, 2H), 2.43 (s, 3H), 3.84 (s, 3H), 4.28 (t, J = 7.5 Hz, 2H), 5.69 (s, 1H), 6.95 (d, J = 8.8 Hz, 2H), 7.76 (s, 1H), 7.82 (d, J = 8.8 Hz, 2H) ppm. ^{13}C{^{1}H} NMR (101 MHz, $CDCl_3$): δ = 11.1 (CH_3), 19.2 (CH_3), 22.0 (CH_2), 48.4 (CH_2), 55.3 (CH_3), 97.8 (CH), 103.5 (CH), 114.1 (CH), 125.9 (C), 127.0 (CH), 141.2 (C), 143.7 (C), 149.7 (C), 157.1 (C), 159.5 (C) ppm. HRMS (ESI+): calcd for $C_{17}H_{20}N_3O_2^+$, 298.1550 [M + H]$^+$; found, 298.1552.

7-Methyl-2-(4-nitrophenyl)-8-propylimidazo[1,2-*a*]pyrimidin-5(8*H*)-one (6e) was obtained in 75% yield (117 mg) from 7-methyl-2-(4-nitrophenyl)imidazo[1,2-*a*]pyrimidin-5(8*H*)-one (**4e**, 135 mg, 0.50 mmol) and 1-bromopropane (**5a**, 70 µL, 0.77 mmol). Mp 224–225 °C (amorphous). ^{1}H NMR (400 MHz, $CDCl_3$): δ = 1.06 (t, J = 7.5 Hz, 3H), 1.87-1.94 (m, 2H), 2.45 (s, 3H), 4.26 (t, J = 7.7 Hz, 2H), 5.70 (s, 1H), 7.97 (s, 1H), 8.00 (d, J = 9.0 Hz, 2H), 8.23 (d, J = 9.0 Hz, 2H) ppm. ^{13}C{^{1}H} NMR (101 MHz, $CDCl_3$): δ = 11.1 (CH_3), 19.3 (CH_3), 22.0 (CH_2), 48.4 (CH_2), 98.0 (CH), 106.9 (CH), 124.0 (CH), 126.1 (CH), 139.1 (C), 139.6 (C), 144.2 (C), 147.0 (C), 150.8 (C), 156.8 (C) ppm. HRMS (ESI+): calcd for $C_{16}H_{17}N_4O_3^+$, 313.1295 [M + H]$^+$; found, 313.1300.

8-Butyl-7-methyl-2-phenylimidazo[1,2-*a*]pyrimidin-5(8*H*)-one (6f) was obtained in 80% yield (87 mg) from 7-methyl-2-phenylimidazo[1,2-*a*]pyrimidin-5(8*H*)-one (**4a**, 115 mg, 0.51 mmol) and 1-bromobutane (**5b**, 80 µL, 0.74 mmol). Mp 145–146 °C (amorphous). ^{1}H NMR (400 MHz, $CDCl_3$): δ = 1.01 (t, J = 7.4 Hz, 3H), 1.42–1.53 (m, 2H), 1.81-1.86 (m, 2H), 2.40 (s, 3H), 4.29 (t, J = 7.7 Hz, 2H), 5.66 (s, 1H), 7.30 (t, J = 7.4 Hz, 1H), 7.41 (t, J = 7.5 Hz, 2H), 7.86 (s, 1H), 7.88 (d, J = 7.6 Hz, 2H) ppm. ^{13}C{^{1}H} NMR (101 MHz, $CDCl_3$): δ = 13.7 (CH_3), 19.2 (CH_2), 20.0 (CH_3), 30.7 (CH_2), 46.7 (CH_2), 97.8 (CH), 104.6 (CH), 125.7 (CH), 127.9 (CH), 128.6 (CH), 133.2 (C), 141.3 (C), 143.8 (C), 150.0 (C), 157.1 (C) ppm. HRMS (ESI+): calcd for $C_{17}H_{20}N_3O^+$, 282.1601 [M + H]$^+$; found, 282.1598.

8-Butyl-2-(4-chlorophenyl)-7-methylimidazo[1,2-*a*]pyrimidin-5(8*H*)-one (6g) was obtained in 82% yield (129 mg) from 2-(4-chlorophenyl)-7-methylimidazo[1,2-*a*]pyrimidin-5(8*H*)-one (**4b**, 130 mg, 0.50 mmol) and 1-bromobutane (**5b**, 80 µL, 0.74 mmol). Mp 116–117 °C (amorphous). ^{1}H NMR (400 MHz, $CDCl_3$): δ = 1.02 (t, J = 7.4 Hz, 3H), 1.45-1.52 (m, 2H), 1.81-1.86 (m, 2H), 2.43 (s, 3H), 4.29 (t, J = 7.8 Hz, 2H), 5.68 (s, 1H), 7.37 (d, J = 8.5 Hz, 2H), 7.80 (d, J = 8.5 Hz, 2H), 7.83 (s, 1H) ppm. ^{13}C{^{1}H} NMR (101 MHz, $CDCl_3$): δ = 13.7 (CH_3), 19.2 (CH_3), 20.0 (CH_2), 30.7 (CH_2), 46.7 (CH_2), 97.9 (CH), 104.7 (CH), 126.9 (CH), 128.8 (CH), 131.7 (C),

133.5 (C), 140.2 (C), 143.8 (C), 150.1 (C), 157.0 (C) ppm. HRMS (ESI+): calcd for $C_{17}H_{19}{}^{35}ClN_3O^+$, 316.1211 [M + H]$^+$; found, 316.1215.[7]

2-(4-Bromophenyl)-8-butyl-7-methylimidazo[1,2-*a*]pyrimidin-5(8*H*)-one (6h) was obtained in 91% yield (164 mg) from 2-(4-bromophenyl)-7-methylimidazo[1,2-*a*]pyrimidin-5(8*H*)-one (**4c**, 152 mg, 0.50 mmol) and 1-bromobutane (**5b**, 80 µL, 0.74 mmol). Mp 130–131 °C (amorphous). ^{1}H NMR (400 MHz, $CDCl_3$): δ = 1.02 (t, *J* = 7.3 Hz, 3H), 1.45-1.51 (m, 2H), 1.81–1.86 (m, 2H), 2.43 (s, 3H), 4.28 (t, *J* = 7.7 Hz, 2H), 5.68 (s, 1H), 7.52 (d, *J* = 8.6 Hz, 2H), 7.74 (d, *J* = 8.6 Hz, 2H), 7.84 (s, 1H) ppm. ^{13}C{^{1}H} NMR (101 MHz, $CDCl_3$): δ = 13.7 (CH_3), 19.2 (CH_3), 20.0 (CH_2), 30.7 (CH_2), 46.7 (CH_2), 97.8 (CH), 104.8 (CH), 121.6 (C), 127.2 (CH), 131.7 (CH), 132.2 (C), 140.2 (C), 143.8 (C), 150.1 (C), 157.0 (C) ppm. HRMS (ESI+): calcd for $C_{17}H_{19}{}^{79}BrN_3O^+$, 360.0706 [M + H]$^+$; found, 360.0710.

8-Butyl-2-(4-methoxyphenyl)-7-methylimidazo[1,2-*a*]pyrimidin-5(8*H*)-one (6i) was obtained in 82% yield (128 mg) from 2-(4-methoxyphenyl)-7-methylimidazo[1,2-*a*]pyrimidin-5(8*H*)-one (**4d**, 128 mg, 0.50 mmol) and 1-bromobutane (**5b**, 80 µL, 0.74 mmol). Mp 99–100 °C (amorphous). ^{1}H NMR (400 MHz, $CDCl_3$): δ = 1.02 (t, *J* = 7.3 Hz, 3H), 1.43-1.52 (m, 2H), 1.81-1.88 (m, 2H), 2.43 (s, 3H), 3.85 (s, 3H), 4.32 (t, *J* = 7.7 Hz, 2H), 5.69 (s, 1H), 6.96 (d, *J* = 8.8 Hz, 2H), 7.77 (s, 1H), 7.82 (d, *J* = 8.8 Hz, 2H) ppm. ^{13}C{^{1}H} NMR (101 MHz, $CDCl_3$): δ = 13.7 (CH_3), 19.1 (CH_3), 20.0 (CH_2), 30.8 (CH_2), 46.8 (CH_2), 55.3 (CH_3), 97.9 (CH), 103.5 (CH), 114.2 (CH), 125.9 (C), 127.1 (CH), 141.2 (C), 143.7 (C), 149.7 (C), 157.1 (C), 159.6 (C) ppm. HRMS (ESI+): calcd for $C_{18}H_{22}N_3O_2^+$, 312.1707 [M + H]$^+$; found, 312.1710.

8-Butyl-7-methyl-2-(4-nitrophenyl)imidazo[1,2-*a*]pyrimidin-5(8*H*)-one (6j) was obtained in 73% yield (119 mg) from 7-methyl-2-(4-nitrophenyl)imidazo[1,2-*a*]pyrimidin-5(8*H*)-one (**4e**, 135 mg, 0.50 mmol) and 1-bromobutane (**5b**, 80 µL, 0.74 mmol). Mp 199–200 °C (amorphous). ^{1}H NMR (400 MHz, $CDCl_3$): δ = 1.03 (t, *J* = 7.4 Hz, 3H), 1.46-1.52 (m, 2H), 1.81–1.89 (m, 2H), 2.46 (s, 3H), 4.31 (t, *J* = 7.8 Hz, 2H), 5.71 (s, 1H), 7.99 (s, 1H), 8.01 (d, *J* = 9.0 Hz, 2H), 8.25 (d, *J* = 9.0 Hz, 2H) ppm. ^{13}C{^{1}H} NMR (101 MHz, $CDCl_3$): δ = 13.7 (CH_3), 19.3 (CH_3), 20.0 (CH_2), 30.7 (CH_2), 46.8 (CH_2), 98.0 (CH), 106.9 (CH), 124.1 (CH), 126.10 (CH), 139.1 (C), 139.6 (C), 144.2 (C), 147.0 (C), 150.8 (C), 156.8 (C) ppm. HRMS (ESI+): calcd for $C_{17}H_{19}N_4O_3^+$, 327.1452 [M + H]$^+$; found, 327.1450.

8-Benzyl-7-methyl-2-phenylimidazo[1,2-*a*]pyrimidin-5(8*H*)-one (6k) was obtained in 90% yield (142 mg) from 7-methyl-2-phenylimidazo[1,2-*a*]pyrimidin-5(8*H*)-one (**4a**, 115 mg, 0.51 mmol) and benzyl bromide (**5c**, 90 µL, 0.76 mmol). Mp 175–176 °C (amorphous). ^{1}H NMR (400 MHz, $CDCl_3$): δ = 2.31 (s, 3H), 5.60 (s, 2H), 5.70 (s, 1H), 7.24-7.36 (m, 6H), 7.39 (t, *J* = 8.0 Hz, 2H), 7.88 (d, *J* = 8.0 Hz, 2H), 7.91 (s, 1H) ppm. ^{13}C{^{1}H} NMR (101 MHz, $CDCl_3$): δ = 19.4 (CH_3), 49.8 (CH_2), 98.1 (CH), 104.9 (CH), 125.7 (CH), 126.7 (CH), 127.9 (CH), 128.1 (CH), 128.6 (CH), 129.0 (CH), 133.1 (C), 135.5 (C), 141.4 (C), 144.6 (C), 150.6 (C), 157.1 (C) ppm. HRMS (ESI+): calcd for $C_{20}H_{18}N_3O^+$, 316.1444 [M + H]$^+$; found, 316.1450.

8-Benzyl-2-(4-chlorophenyl)-7-methylimidazo[1,2-*a*]pyrimidin-5(8*H*)-one (6l) was obtained in 94% yield (164 mg) from 2-(4-chlorophenyl)-7-methylimidazo[1,2-*a*]pyrimidin-5(8*H*)-one (**4b**, 130 mg, 0.50 mmol)

and benzyl bromide (**5c**, 90 µL, 0.76 mmol). Mp 227–228 °C (amorphous). ^{1}H NMR (400 MHz, $CDCl_3$): δ = 2.34 (s, 3H), 5.60 (s, 2H), 5.72 (s, 1H), 7.23-7.36 (m, 7H), 7.80 (d, J = 8.3 Hz, 2H), 7.89 (s, 1H) ppm. $^{13}C\{^{1}H\}$ NMR (101 MHz, $CDCl_3$): δ = 19.4 (CH_3), 49.9 (CH_2), 98.3 (CH), 105.1 (CH), 126.6 (CH), 126.9 (CH), 128.2 (CH), 128.8 (CH), 129.1 (CH), 131.7 (C), 133.6 (C), 135.5 (C), 140.5 (C), 144.7 (C), 150.7 (C), 157.0 (C) ppm. HRMS (ESI+): calcd for $C_{16}H_{17}{}^{35}ClN_3O^+$, 350.1055 [M + H]$^+$; found, 350.1058.[7]

8-Benzyl-2-(4-bromophenyl)-7-methylimidazo[1,2-*a*]pyrimidin-5(8*H*)-one (6m) was obtained in 96% yield (189 mg) from 2-(4-bromophenyl)-7-methylimidazo[1,2-*a*]pyrimidin-5(8*H*)-one (**4c**, 152 mg, 0.50 mmol) and benzyl bromide (**5c**, 90 µL, 0.76 mmol). Mp 232–233 °C (amorphous). ^{1}H NMR (400 MHz, $CDCl_3$): δ = 2.34 (s, 3H), 5.60 (s, 2H), 5.72 (s, 1H), 7.24 (d, J = 7.4 Hz, 2H), 7.29-7.37 (m, 3H), 7.51 (d, J = 8.5 Hz, 2H), 7.74 (d, J = 8.5 Hz, 2H), 7.90 (s, 1H) ppm. $^{13}C\{^{1}H\}$ NMR (101 MHz, $CDCl_3$): δ = 19.4 (CH_3), 49.9 (CH_2), 98.3 (CH), 105.2 (CH), 121.8 (C), 126.6 (CH), 127.2 (CH), 128.2 (CH), 129.1 (CH), 131.8 (CH), 132.2 (C), 135.4 (C), 140.5 (C), 144.7 (C), 150.8 (C), 157.0 (C) ppm. HRMS (ESI+): calcd for $C_{20}H_{17}{}^{79}BrN_3O^+$, 394.0550 [M + H]$^+$; found, 394.0554.

8-Benzyl-2-(4-methoxyphenyl)-7-methylimidazo[1,2-*a*]pyrimidin-5(8*H*)-one (6n) was obtained in 91% yield (157 mg) from 2-(4-methoxyphenyl)-7-methylimidazo[1,2-*a*]pyrimidin-5(8*H*)-one (**4d**, 128 mg, 0.50 mmol) and benzyl bromide (**5c**, 90 µL, 0.76 mmol). Mp 207–208 °C (amorphous). ^{1}H NMR (400 MHz, $CDCl_3$): δ = 2.33 (s, 3H), 3.84 (s, 3H), 5.62 (s, 2H), 5.71 (s, 1H), 6.94 (d, J = 8.8 Hz, 2H), 7.25-7.36 (m, 5H), 7.80-7.83 (m, 3H) ppm. $^{13}C\{^{1}H\}$ NMR (101 MHz, $CDCl_3$): δ = 19.4 (CH_3), 49.9 (CH_2), 55.3 (CH_3), 98.2 (CH), 103.8 (CH), 114.1 (CH), 126.0 (C), 126.7 (CH), 127.0 (CH), 128.1 (CH), 129.1 (CH), 135.7 (C), 141.5 (C), 144.6 (C), 150.3 (C), 157.1 (C), 159.6 (C) ppm. HRMS (ESI+): calcd for $C_{21}H_{20}N_3O_2^+$, 346.1550 [M + H]$^+$; found, 346.1552.

8-Benzyl-7-methyl-2-(4-nitrophenyl)imidazo[1,2-*a*]pyrimidin-5(8*H*)-one (6o) was obtained in 84% yield (151 mg) from 7-methyl-2-(4-nitrophenyl)imidazo[1,2-*a*]pyrimidin-5(8*H*)-one (**4d**, 135 mg, 0.50 mmol) and benzyl bromide (**5c**, 90 µL, 0.76 mmol). Mp 230–231 °C (amorphous). ^{1}H NMR (400 MHz, $CDCl_3$): δ = 2.37 (s, 3H), 5.63 (s, 2H), 5.76 (s, 1H), 7.24-7.39 (m, 5H), 8.01 (d, J = 9.0 Hz, 2H), 8.05 (s, 1H), 8.25 (d, J = 9.0 Hz, 2H) ppm. $^{13}C\{^{1}H\}$ NMR (101 MHz, $CDCl_3$): δ = 19.5 (CH_3), 50.0 (CH_2), 98.5 (CH), 107.3 (CH), 124.1 (CH), 126.1 (CH), 126.6 (CH), 128.3 (CH), 129.2 (CH), 135.2 (C), 139.3 (C), 139.6 (C), 145.1 (C), 147.1 (C), 151.4 (C), 156.8 (C) ppm. HRMS (ESI+): calcd for $C_{20}H_{17}N_4O_3^+$, 361.1295 [M + H]$^+$; found, 361.1299.

Ethyl 2-(2-(4-chlorophenyl)-7-methyl-5-oxoimidazo[1,2-*a*]pyrimidin-8(5*H*)-yl)acetate (6p) was obtained in 79% yield (139 mg) from 2-(4-chlorophenyl)-7-methylimidazo[1,2-*a*]pyrimidin-5(8*H*)-one (**4b**, 115 mg, 0.51 mmol) and ethyl bromoacetate (**5d**, 90 µL, 0.81 mmol). Mp 190–191 °C (amorphous). ^{1}H NMR (400 MHz, $CDCl_3$): δ = 1.32 (t, J = 7.2 Hz, 3H) 2.36 (s, 3H), 4.29 (q, J = 7.2 Hz, 2H), 5.08 (s, 2H), 5.76 (s, 1H), 7.36 (d, J = 8.5 Hz, 2H), 7.77 (d, J = 8.4 Hz, 2H), 7.84 (s, 1H) ppm. $^{13}C\{^{1}H\}$ NMR (101 MHz, $CDCl_3$): δ = 14.1 (CH_3), 19.1 (CH_3), 47.1 (CH_2), 62.4 (CH_2), 98.6 (CH), 105.2 (CH), 126.9 (CH), 128.8 (CH), 131.5 (C), 133.5 (C), 140.2 (C), 143.9 (C), 150.0 (C), 156.9 (C), 167.2 (C) ppm. HRMS (ESI+): calcd for $C_{17}H_{17}{}^{35}ClN_3O_3^+$, 346.0953 [M + H]$^+$; found, 346.0952.[7]

2.2.4. Synthesis of 2-aryl-3,6-dibromo-7-methyl-8-propylimidazo[1,2-a]pyrimidin-5(8H)-ones 8a-e.

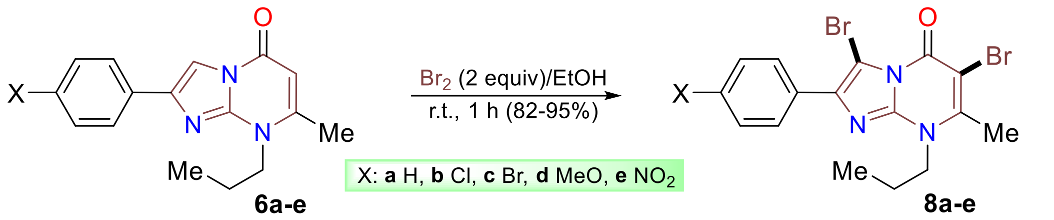

To a solution of 2-aryl-7-methyl-8-propylimidazo[1,2-*a*]pyrimidin-5(8*H*)-one **6a-e** (0.50 mmol) in absolute ethanol (2 mL), bromine (1 mmol, 52 µL) was added drop by dropwise over 5 min. The reaction mixture was then stirred at room temperature for 1 h. Subsequently, distilled water (5.0 mL) was added, and the resulting mixture was extracted with dichloromethane (3 × 10 mL). The organic phase was dried over anhydrous $MgSO_4$, filtered, and the solution was concentrated under vacuum. The crude residue was purified by column flash chromatography on silica gel (eluent: DCM or DCM/*n*-hexane as eluents), resulting in the pure product **8a-e** as withe/yellow solids in high yields.

3,6-Dibromo-7-methyl-2-phenyl-8-propylimidazo[1,2-*a*]pyrimidin-5(8*H*)-one (8a) was obtained in 89% yield (189 mg) from 7-methyl-2-phenyl-8-propylimidazo[1,2-*a*]pyrimidin-5(8*H*)-one (**6a**, 133 mg, 0.5 mmol). Mp 185–186 °C (amorphous). ^{1}H NMR (400 MHz, $CDCl_3$): δ = 1.05 (t, J = 7.4 Hz, 3H), 1.83–1.91 (m, 2H), 2.68 (s, 3H), 4.35 (t, J = 7.4 Hz, 2H), 7.36 (t, J = 7.4 Hz, 1H), 7.44 (t, J = 7.4 Hz, 2H), 8.03 (d, J = 8.1 Hz, 2H) ppm. ^{13}C{^{1}H} NMR (101 MHz, $CDCl_3$): δ = 11.1 (CH_3), 19.8 (CH_3), 22.0 (CH_2), 49.7 (CH_2), 89.7 (C), 95.6 (C), 128.0 (CH), 128.3 (CH x 2), 132.1 (C), 139.9 (C), 143.0 (C), 148.2 (C), 154.3 (C) ppm. HRMS (ESI+): calcd for $C_{16}H_{16}{}^{79}Br_2N_3O^+$, 423.9655 [M + H]$^+$; found, 423.9660.

3,6-Dibromo-2-(4-chlorophenyl)-7-methyl-8-propylimidazo[1,2-*a*]pyrimidin-5(8*H*)-one (8b) was obtained in 93% yield (214 mg) from 2-(4-chlorophenyl)-7-methyl-8-propylimidazo[1,2-*a*]pyrimidin-5(8*H*)-one (**6b**, 150 mg, 0.5 mmol). Mp 229–230 °C (amorphous). ^{1}H NMR (400 MHz, $CDCl_3$): δ = 1.05 (t, J = 7.4 Hz, 3H), 1.84–1.91 (m, 2H), 2.68 (s, 3H), 4.34 (t, J = 7.8 Hz, 2H), 7.40 (t, J = 8.7 Hz, 2H), 7.99 (t, J = 8.7 Hz, 2H) ppm. ^{13}C{^{1}H} NMR (101 MHz, $CDCl_3$): δ = 11.1 (CH_3), 19.8 (CH_3), 22.0 (CH_2), 49.7 (CH_2), 89.8 (C), 95.7 (C), 128.5 (CH), 129.2 (CH), 130.6 (C), 134.2 (C), 138.8 (C), 143.0 (C), 148.4 (C), 154.3 (C) ppm. HRMS (ESI+): calcd for $C_{16}H_{15}{}^{79}Br_2{}^{35}ClN_3O^+$, 457.9265 [M + H]$^+$; found, 457.9283.

3,6-Dibromo-2-(4-bromophenyl)-7-methyl-8-propylimidazo[1,2-*a*]pyrimidin-5(8*H*)-one (8c) was obtained in 95% yield (239 mg) from 2-(4-bromophenyl)-7-methyl-8-propylimidazo[1,2-*a*]pyrimidin-5(8*H*)-one (**6c**, 173 mg, 0.5 mmol). Mp 223–224 °C (amorphous). ^{1}H NMR ($CDCl_3$, $CDCl_3$,): δ = 1.05 (t, J = 7.5 Hz, 3H), 1.82–1.92 (m, 2H), 2.69 (s, 3H), 4.34 (t, J = 7.9 Hz, 2H), 7.56 (d, J = 8.7 Hz, 2H), 7.93 (d, J = 8.7 Hz, 2H) ppm. ^{13}C{^{1}H} NMR (101 MHz, $CDCl_3$): δ = 11.1 (CH_3), 19.8 (CH_3), 22.0 (CH_2), 49.7 (CH_2), 89.9 (C), 95.8 (C), 122.5 (C), 129.4 (CH), 131.0 (C), 131.5 (CH), 138.9 (C), 143.0 (C), 148.4 (C), 154.3 (C) ppm. HRMS (ESI+): calcd for $C_{16}H_{15}{}^{79}Br_3N_3O^+$, 501.8760 [M + H]$^+$; found, 501.8757.

3,6-Dibromo-2-(4-methoxyphenyl)-7-methyl-8-propylimidazo[1,2-*a*]pyrimidin-5(8*H*)-one (8d) was obtained in 90% yield (205 mg) from 2-(4-methoxyphenyl)-7-methyl-8-propylimidazo[1,2-*a*]pyrimidin-5(8*H*)-one (**6d**, 148 mg, 0.5 mmol). Mp 200–201 °C (amorphous). ^{1}H NMR (400 MHz, $CDCl_3$): δ = 1.04 (t, J = 7.4 Hz, 3H), 1.81–1.91 (m, 2H), 2.66 (s, 3H), 3.85 (s, 3H), 4.33 (t, J = 7.8 Hz, 2H), 6.97 (d, J = 8.8 Hz, 2H), 7.98 (d, J = 8.8 Hz, 2H) ppm. ^{13}C{^{1}H} NMR (101 MHz, $CDCl_3$): δ = 11.1 (CH_3), 19.7 (CH_3), 21.9 (CH_2), 49.7 (CH_2), 55.3 (CH_3), 88.6 (C), 95.6 (C), 113.7 (CH), 124.6 (C), 129.2 (CH), 139.7 (C), 142.8 (C), 148.0 (C), 154.3 (C), 159.7 (C) ppm. HRMS (ESI+): calcd for $C_{17}H_{18}{}^{79}Br_2N_3O_2^+$, 453.9760 [M + H]$^+$; found, 453.9764.

3,6-Dibromo-7-methyl-2-(4-nitrophenyl)-8-propylimidazo[1,2-*a*]pyrimidin-5(8*H*)-one (8e) was obtained in 82% yield (193 mg) from 7-methyl-2-(4-nitrophenyl)-8-propylimidazo[1,2-*a*]pyrimidin-5(8*H*)-one (**6e**, 235 mg, 0.5 mmol). Mp 227–228 °C (amorphous). ^{1}H NMR (400 MHz, $CDCl_3$): δ = 1.08 (t, J = 7.5 Hz, 3H), 1.84–1.92 (m, 2H), 2.71 (s, 3H), 4.37 (t, J = 7.9 Hz, 2H), 8.27 (s, 4H) ppm. ^{13}C{^{1}H} NMR (101 MHz, $CDCl_3$): δ = 11.1 (CH_3), 19.9 (CH_3), 22.0 (CH_2), 49.9 (CH_2), 91.9 (C), 95.9 (C), 123.5 (CH), 128.4 (CH), 137.6 (C), 138.5 (C), 143.4 (C), 147.2 (C), 149.0 (C), 154.2 (C) ppm. HRMS (ESI+): calcd for $C_{16}H_{15}{}^{79}Br_2N_4O_3^+$, 468.9505 [M + H]$^+$; found, 468.9510.

3. Copies of NMR spectra

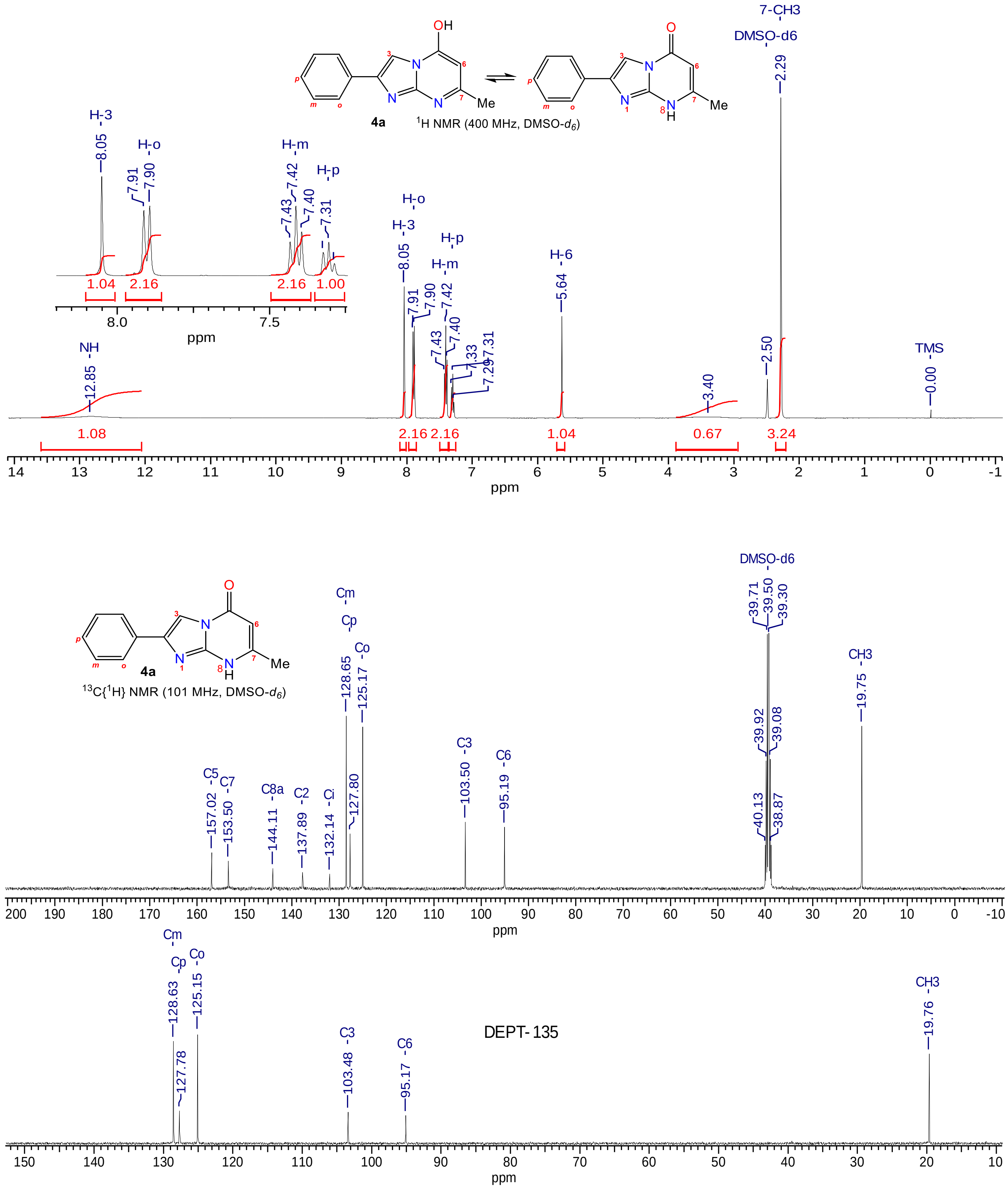

Fig. S3 ^{1}H and ^{13}C{^{1}H} NMR spectra of 7-methyl-2-phenylimidazo[1,2-*a*]pyrimidin-5(8*H*)-one (**4a**)

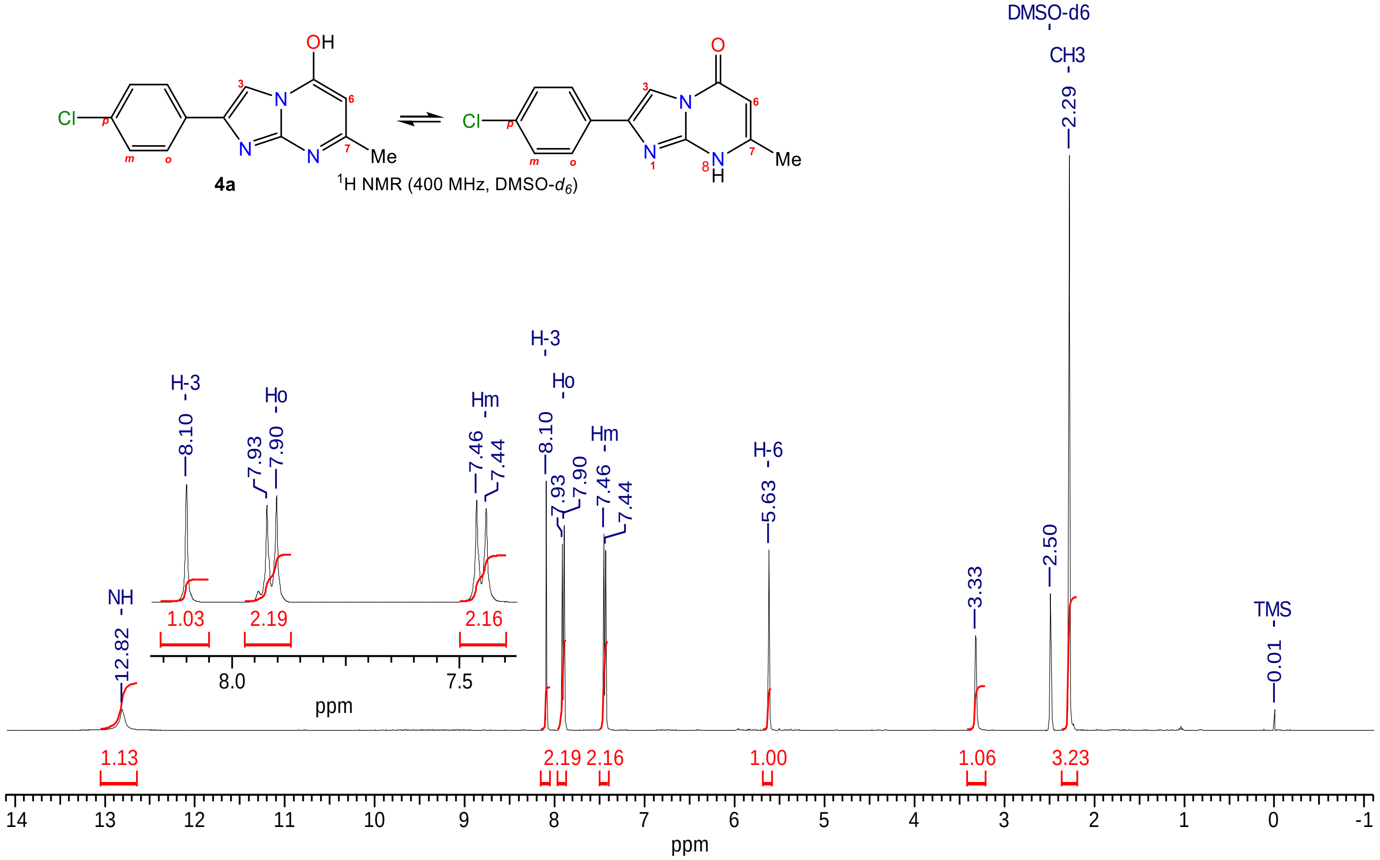

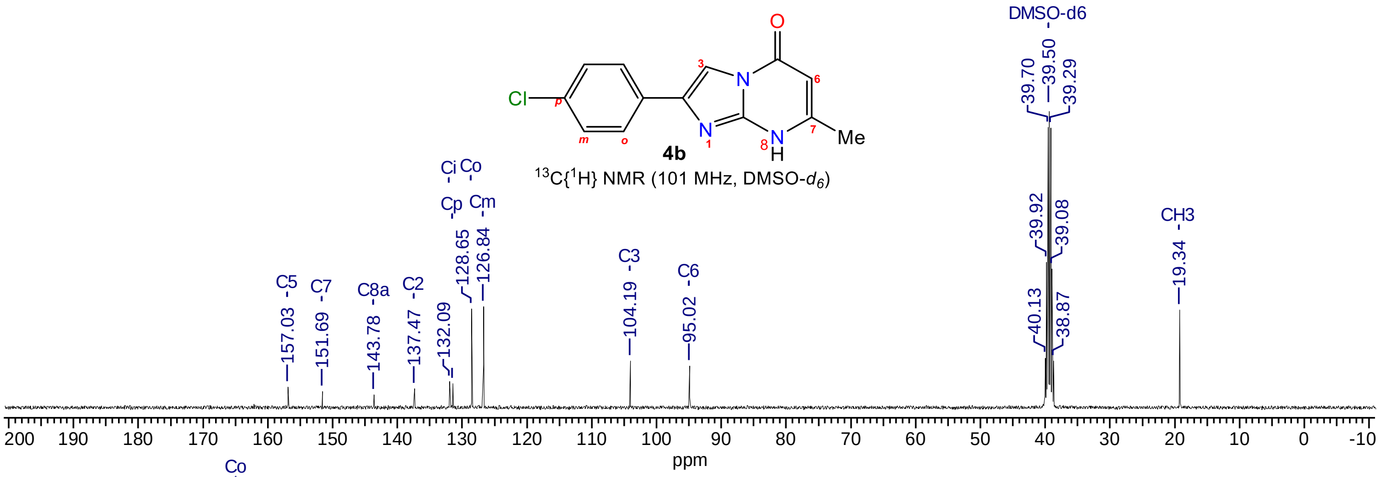

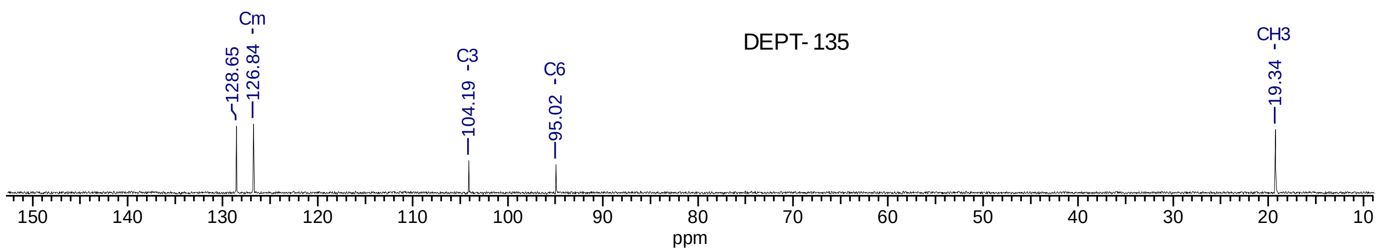

Fig. S4 ^{1}H and ^{13}C{^{1}H} NMR spectra of 2-(4-chlorophenyl)-7-methylimidazo[1,2-*a*]pyrimidin-5(8*H*)-one (**4b**)

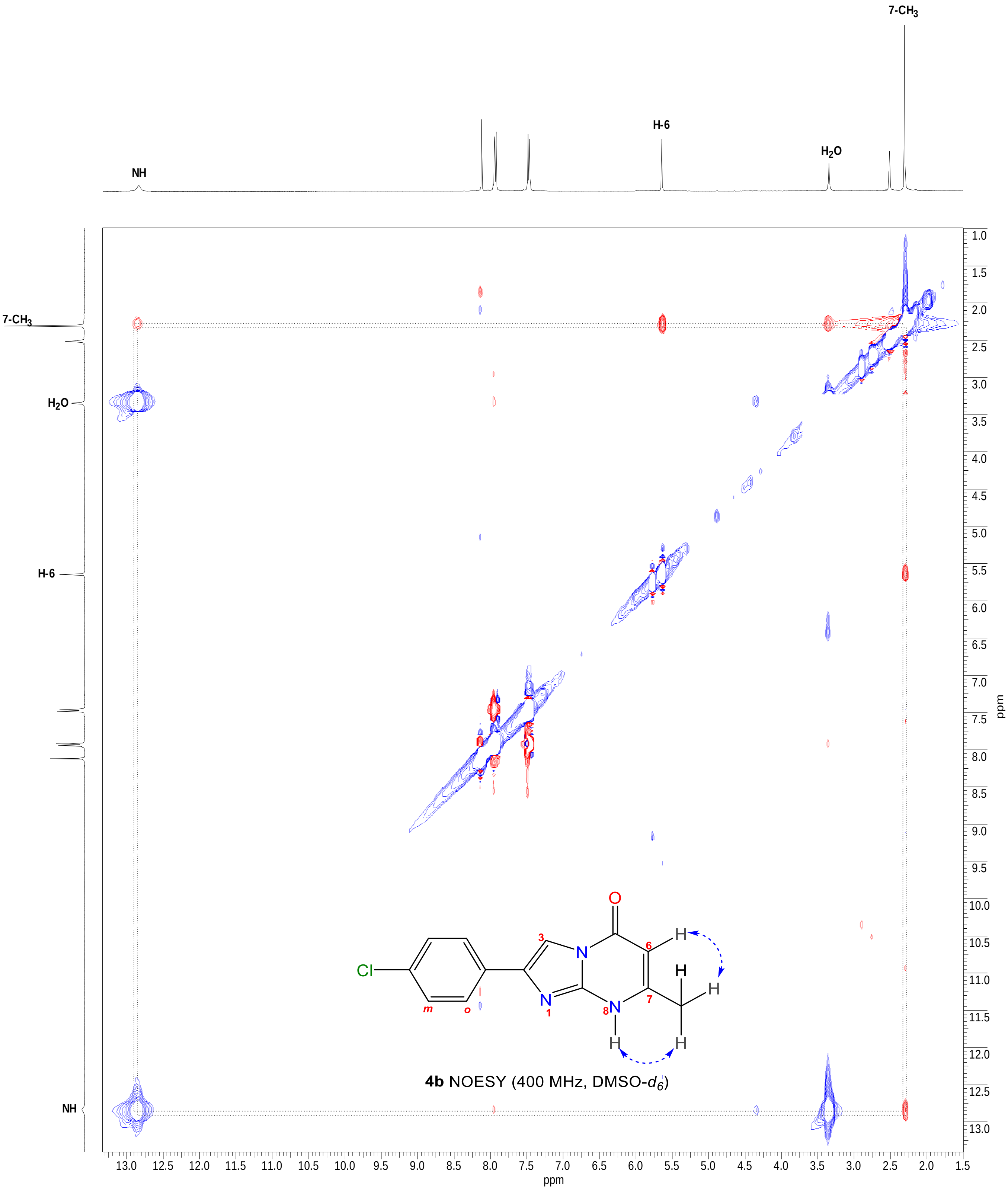

Fig. S5 ^{1}H and ^{13}C{^{1}H} NMR spectra of 2-(4-chlorophenyl)-7-methylimidazo[1,2-*a*]pyrimidin-5(8*H*)-one (**4b**)

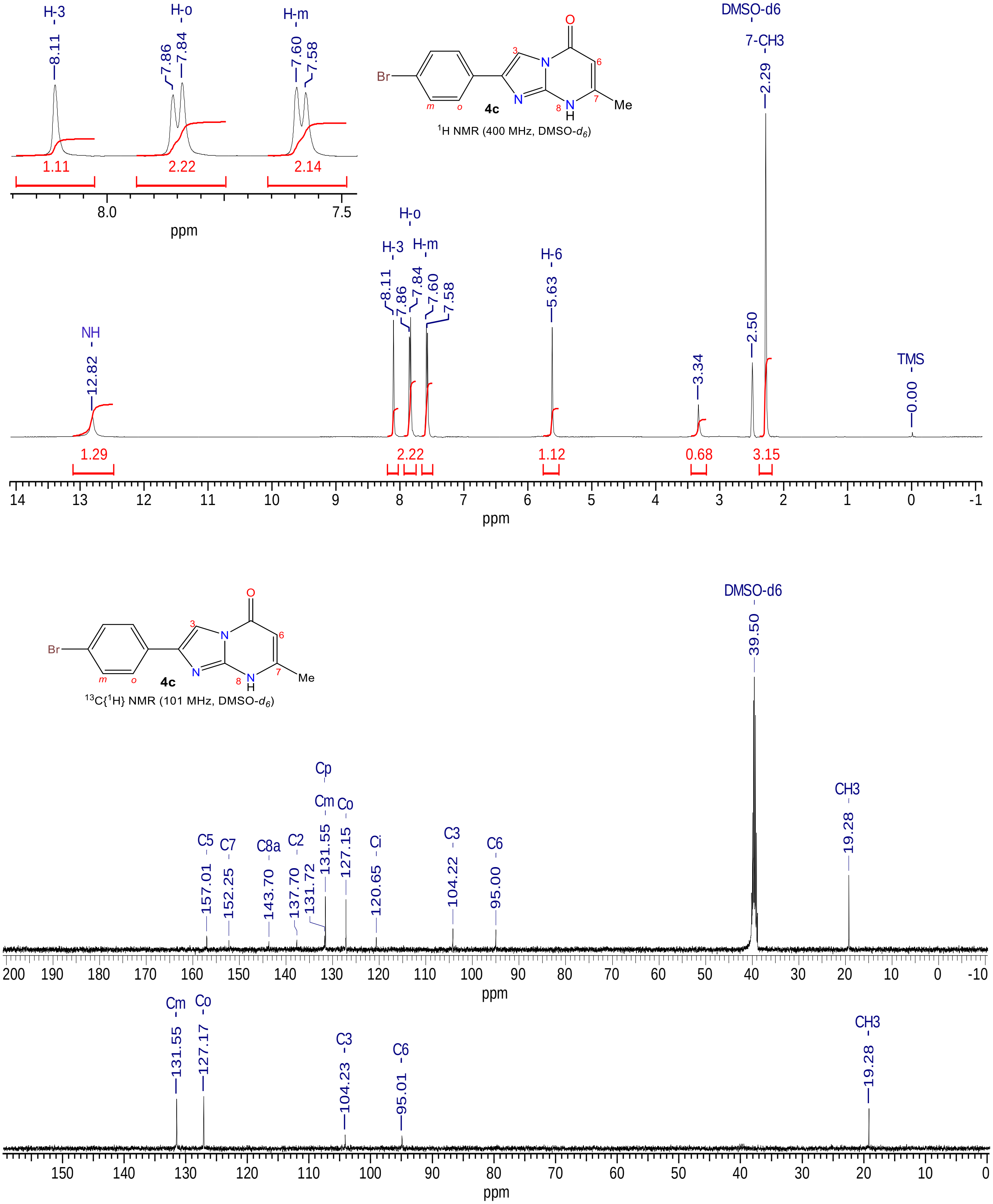

Fig. S6 ^{1}H and ^{13}C{^{1}H} NMR spectra of 2-(4-bromophenyl)-7-methylimidazo[1,2-*a*]pyrimidin-5(8*H*)-one (**4c**)

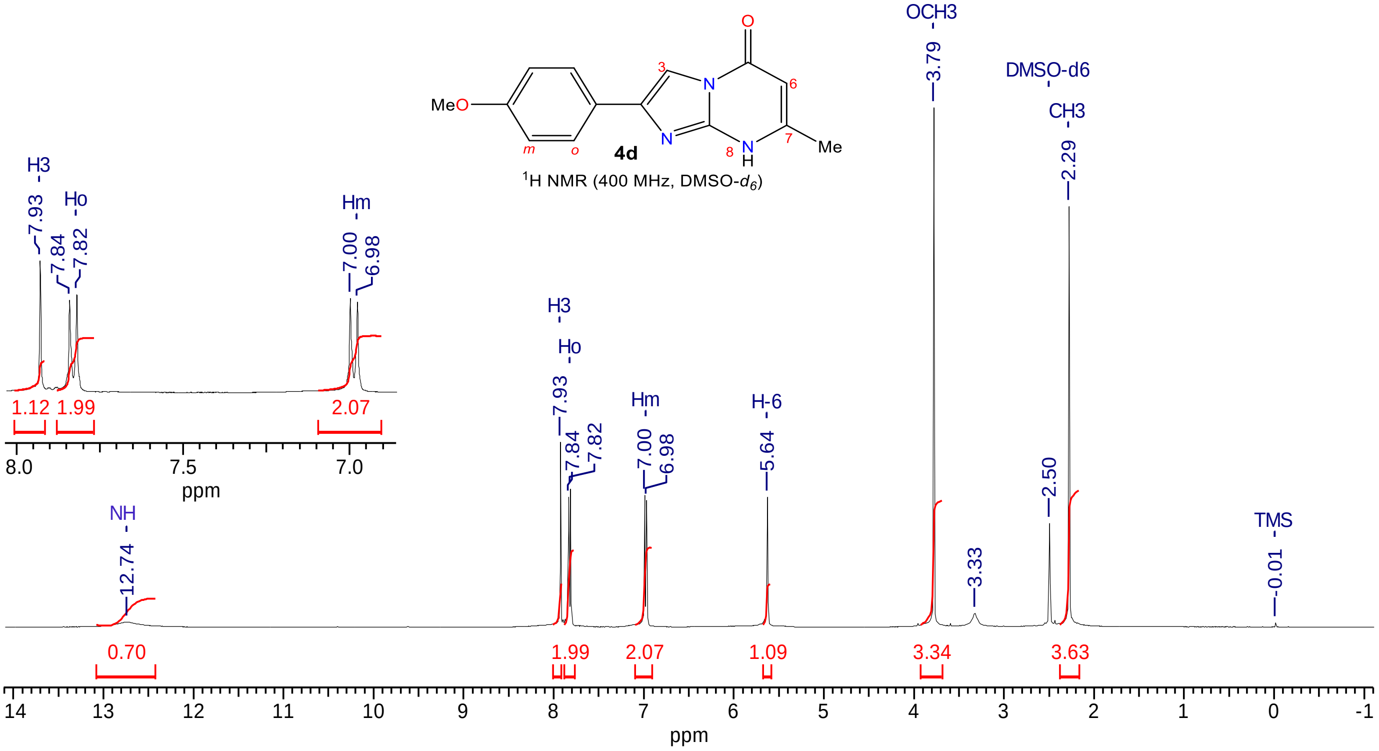

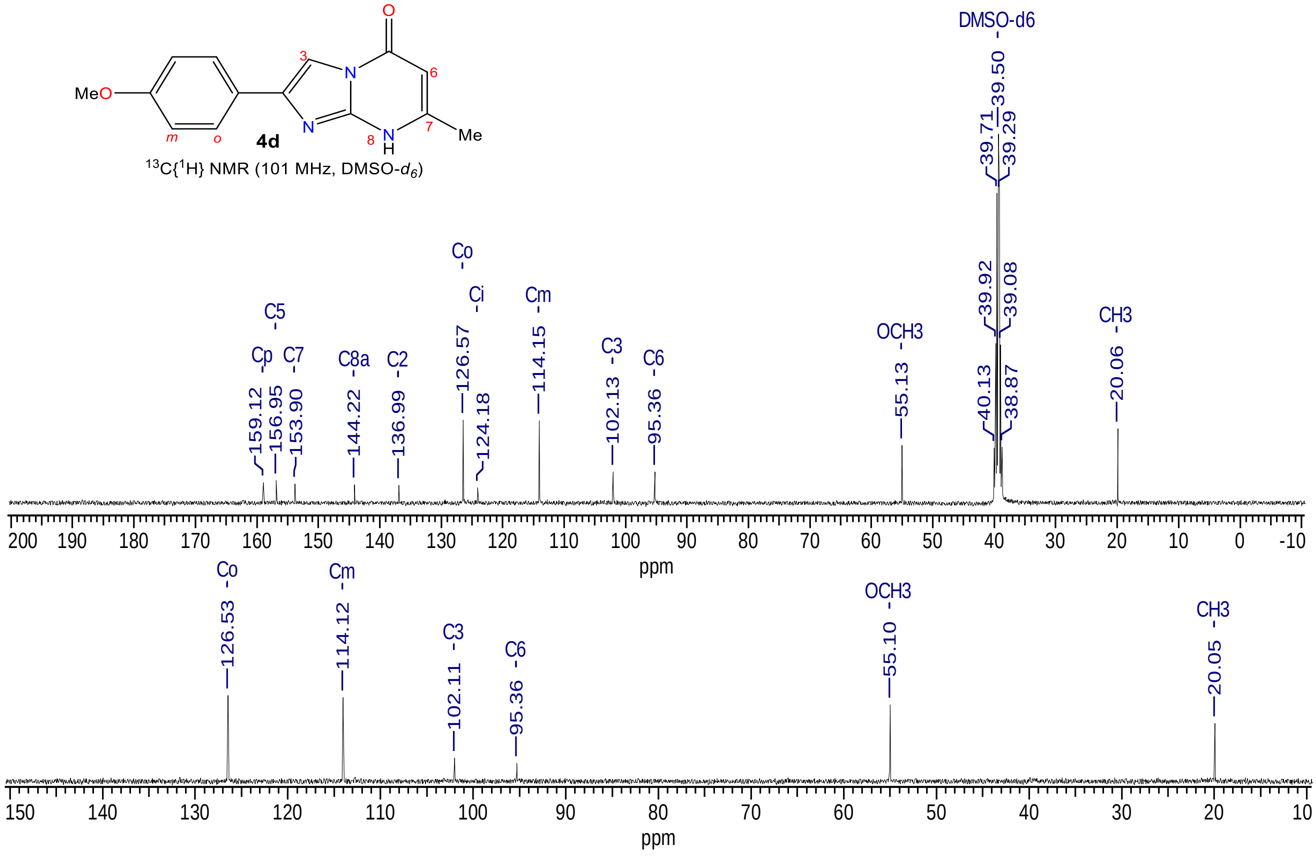

Fig. S7 ^{1}H and $^{13}C\{^{1}H\}$ NMR spectra of 2-(4-methoxyphenyl)-7-methylimidazo[1,2-*a*]pyrimidin-5(8*H*)-one (**4d**)

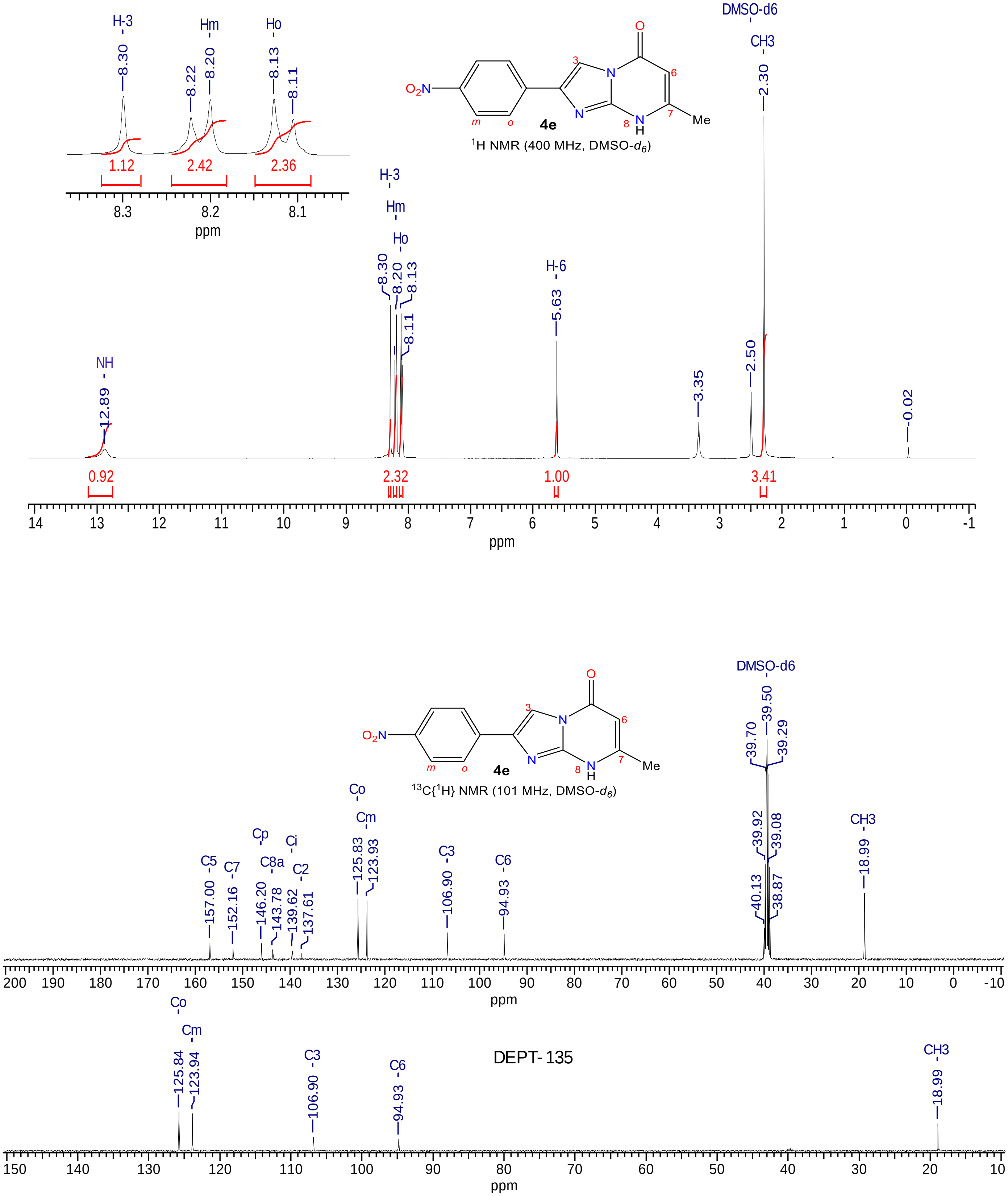

Fig. S8 ^{1}H and $^{13}C\{^{1}H\}$ NMR spectra of 7-methyl-2-(4-nitrophenyl)imidazo[1,2-*a*]pyrimidin-5(8*H*)-one (**4e**)

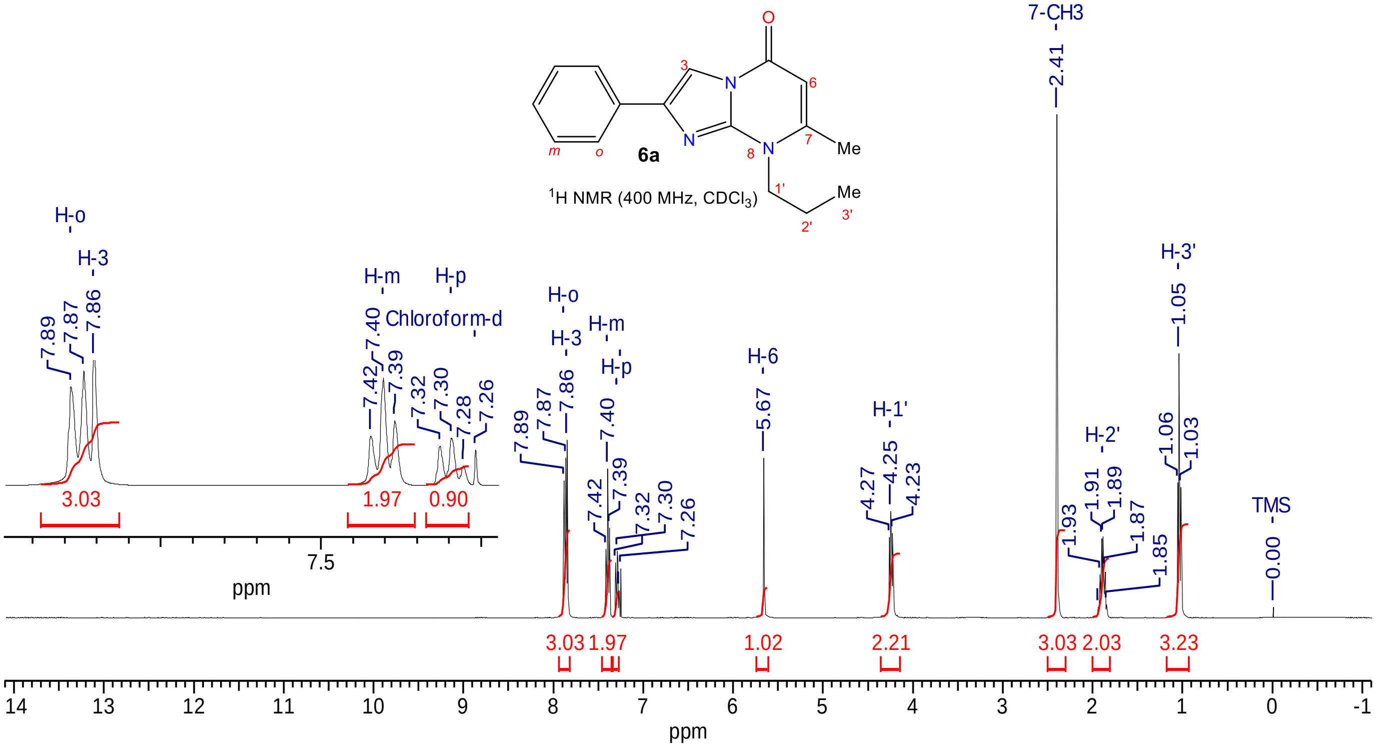

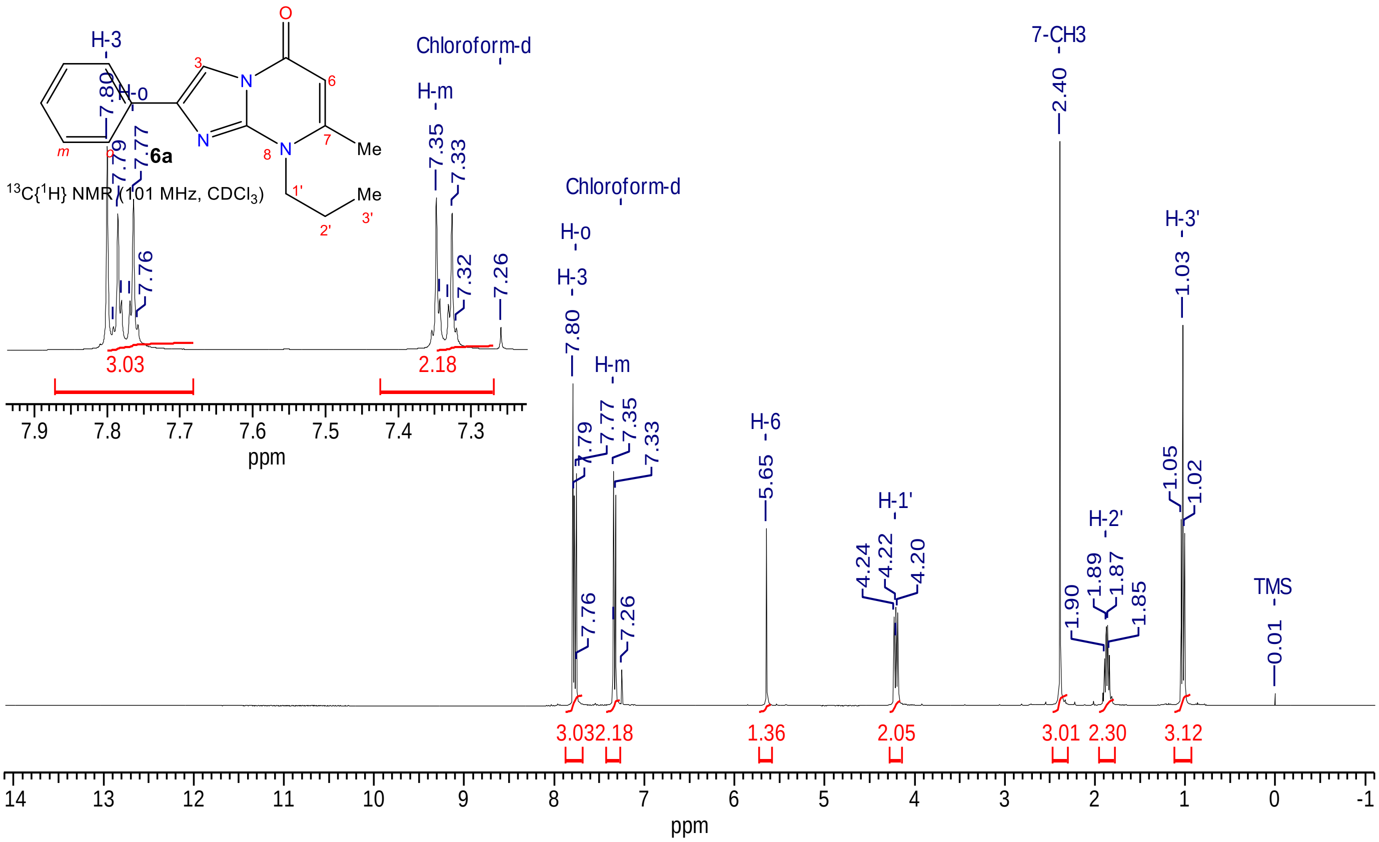

Fig. S9 ^{1}H and $^{13}C\{^{1}H\}$ NMR spectra of 7-methyl-2-phenyl-8-propylimidazo[1,2-*a*]pyrimidin-5(8*H*)-one (**6a**)

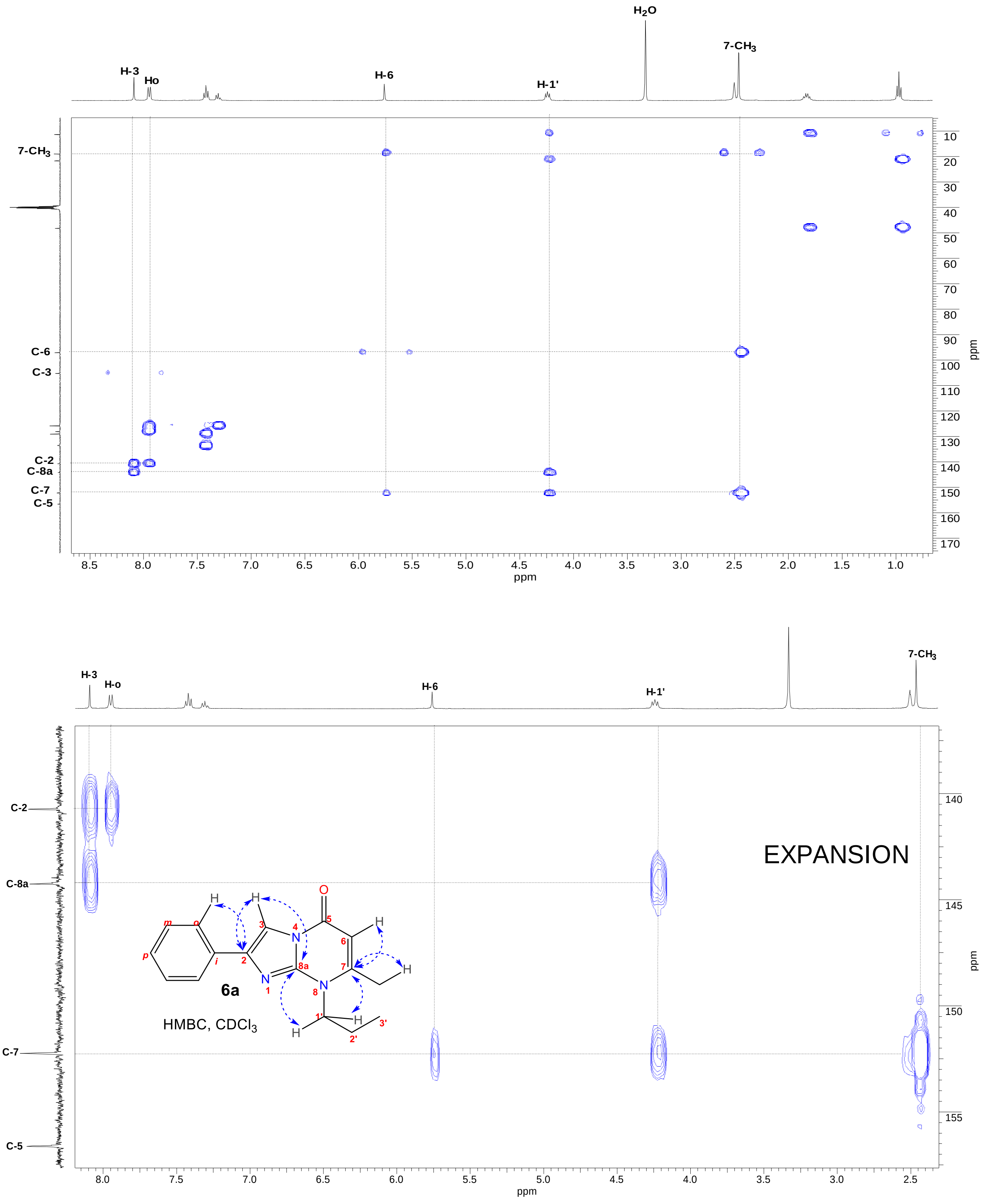

Fig. S10 HMBC spectra of 7-methyl-2-phenyl-8-propylimidazo[1,2-*a*]pyrimidin-5(8*H*)-one (**6a**)

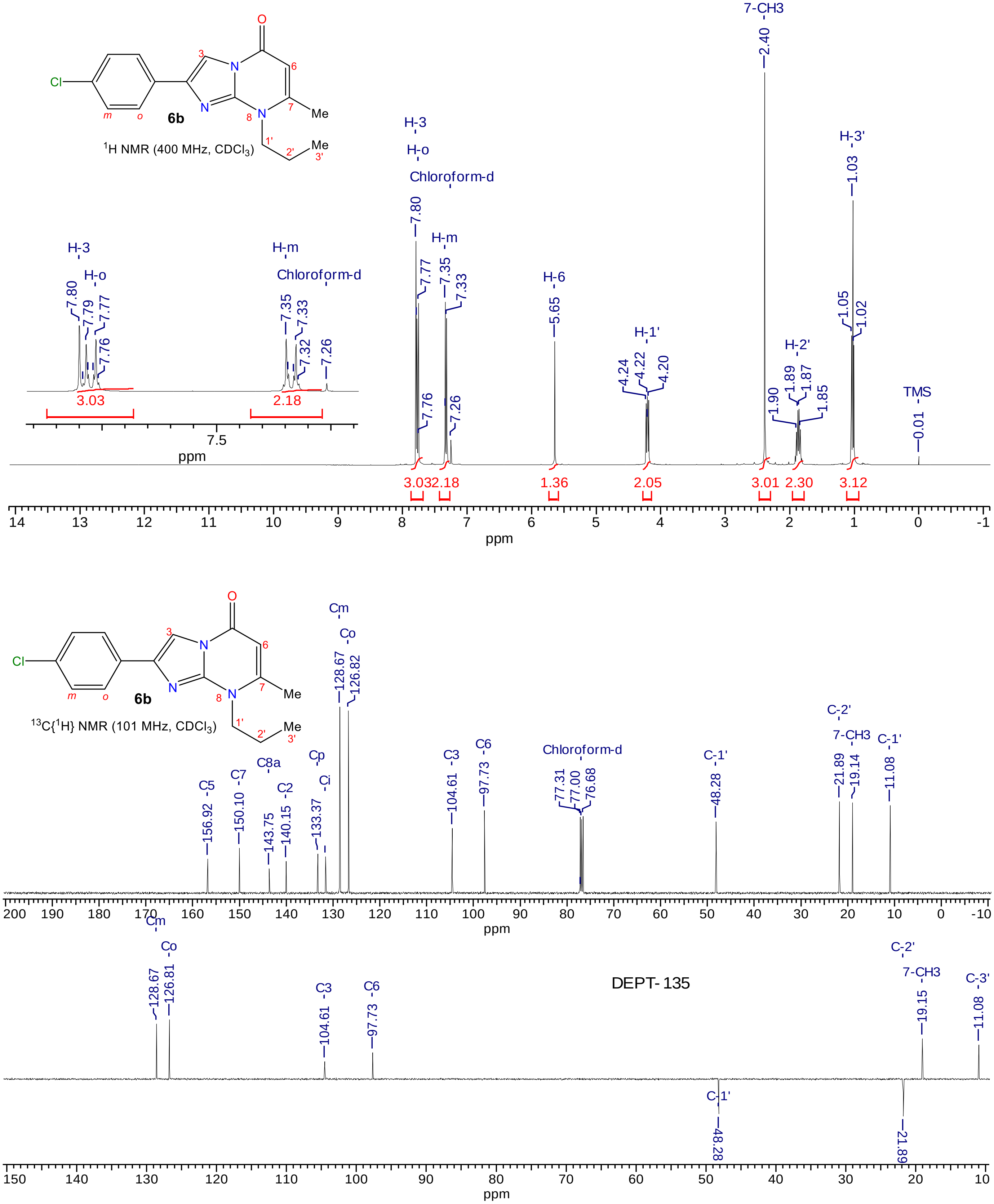

Fig. S11 ^{1}H and ^{13}C{^{1}H} NMR spectra of 2-(4-chlorophenyl)-7-methyl-8-propylimidazo[1,2-*a*]pyrimidin-5-one (**6b**)

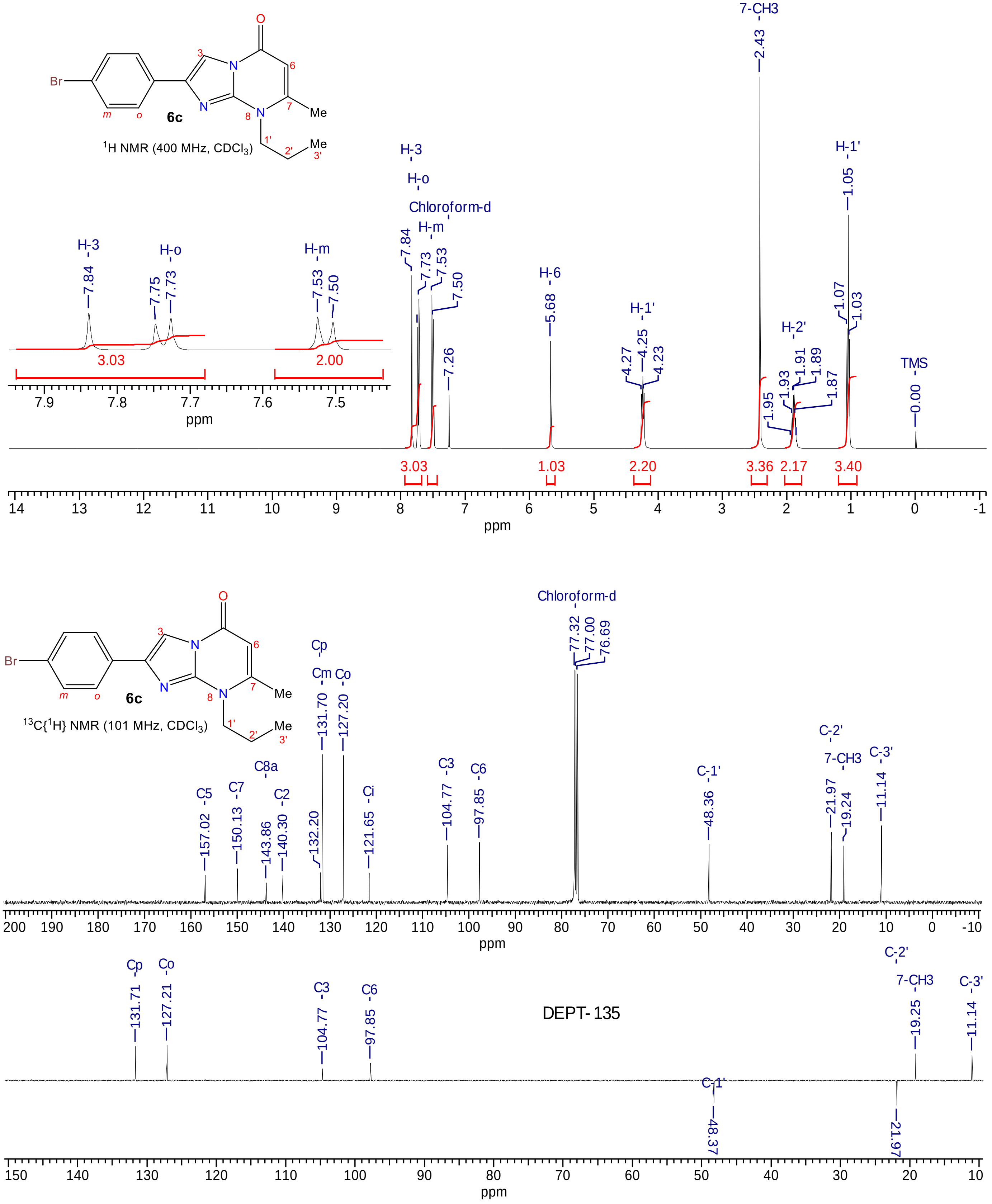

Fig. S12 ¹H and ¹³C{¹H} NMR spectra of 2-(4-bromophenyl)-7-methyl-8-propylimidazo[1,2-*a*]pyrimidin-5-one (**6c**)

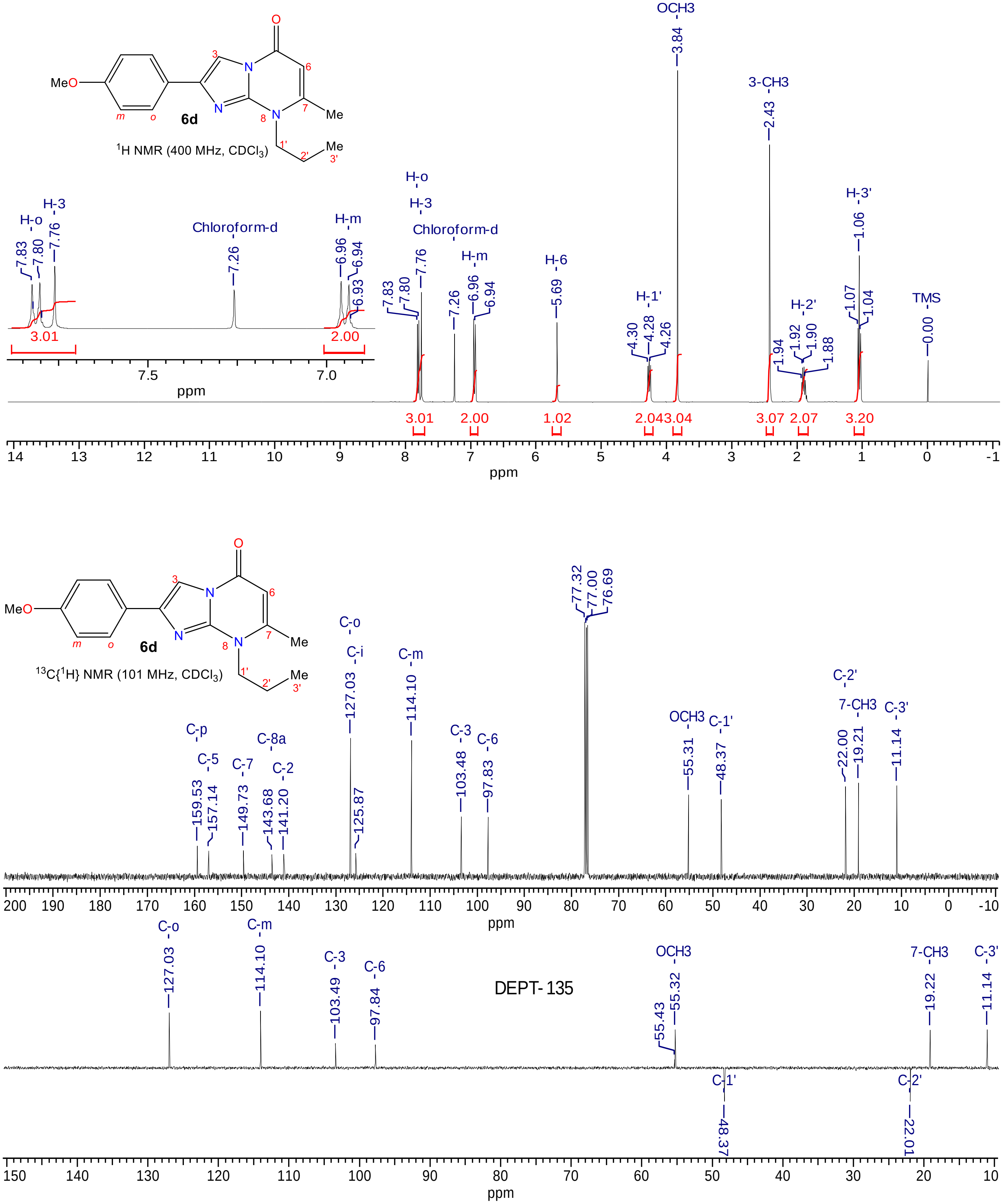

Fig. S13 ^{1}H and $^{13}C\{^{1}H\}$ NMR spectra of 2-(4-methoxyphenyl)-7-methyl-8-propylimidazo[1,2-*a*]pyrimidin-5(8*H*)-one (**6d**)

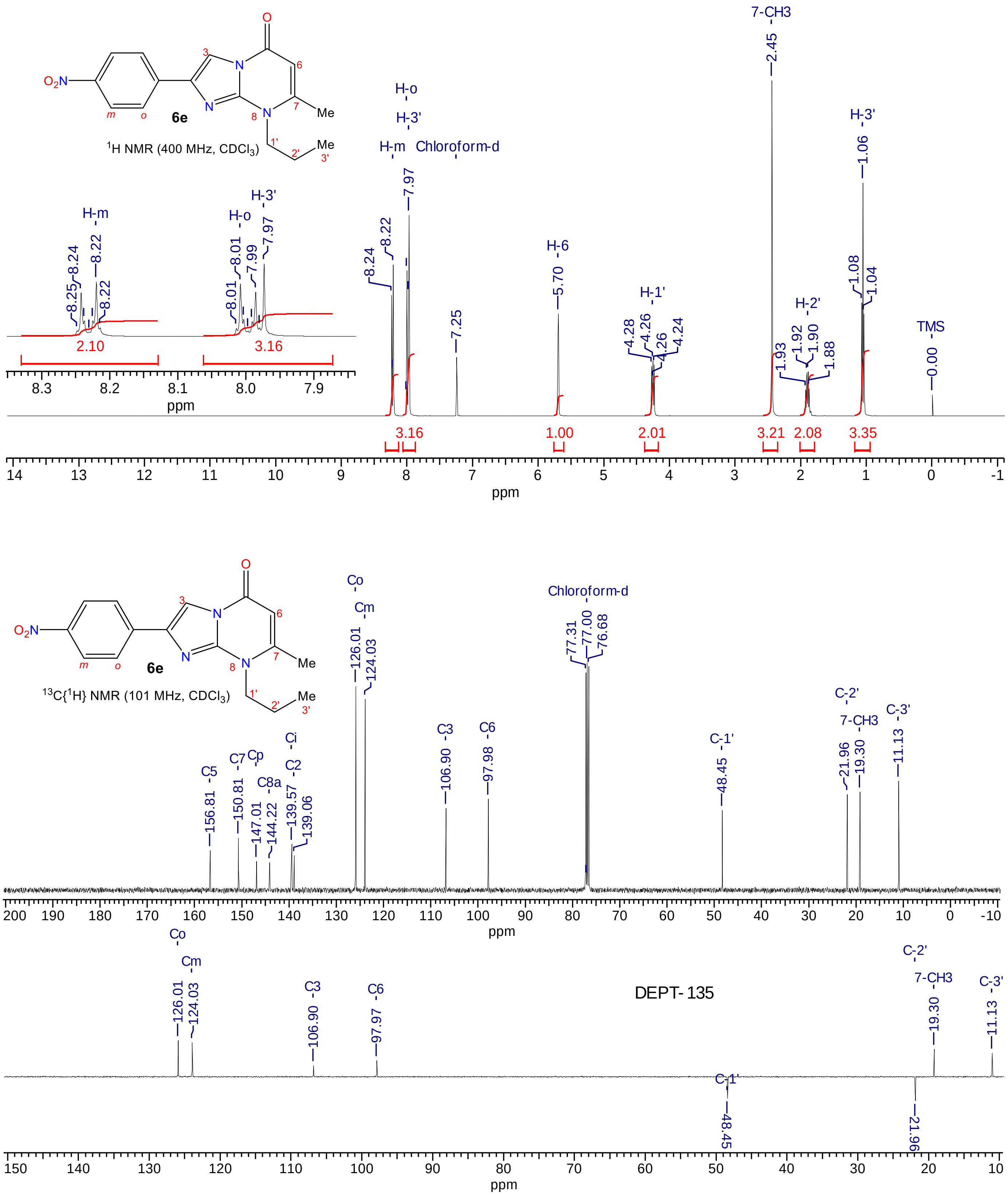

Fig. S14 ^{1}H and ^{13}C{^{1}H} NMR spectra of 7-methyl-2-(4-nitrophenyl)-8-propylimidazo[1,2-*a*]pyrimidin-5(8*H*)-one (**6e**)

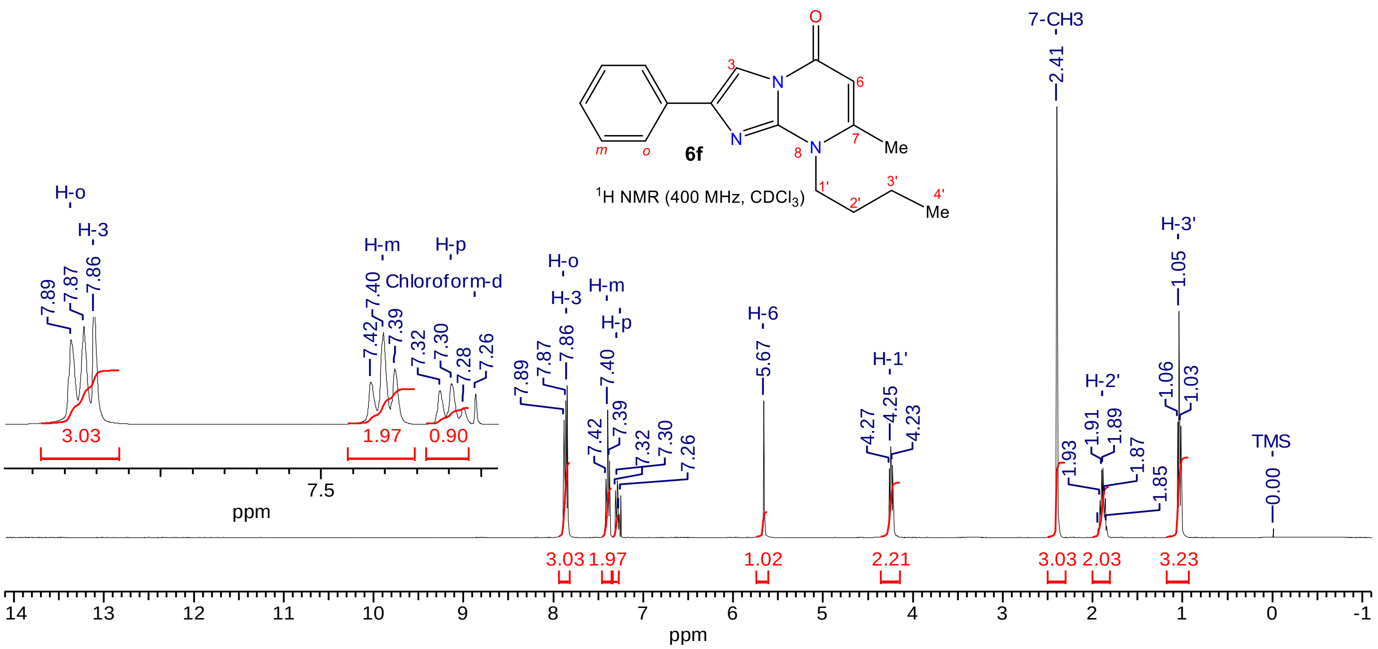

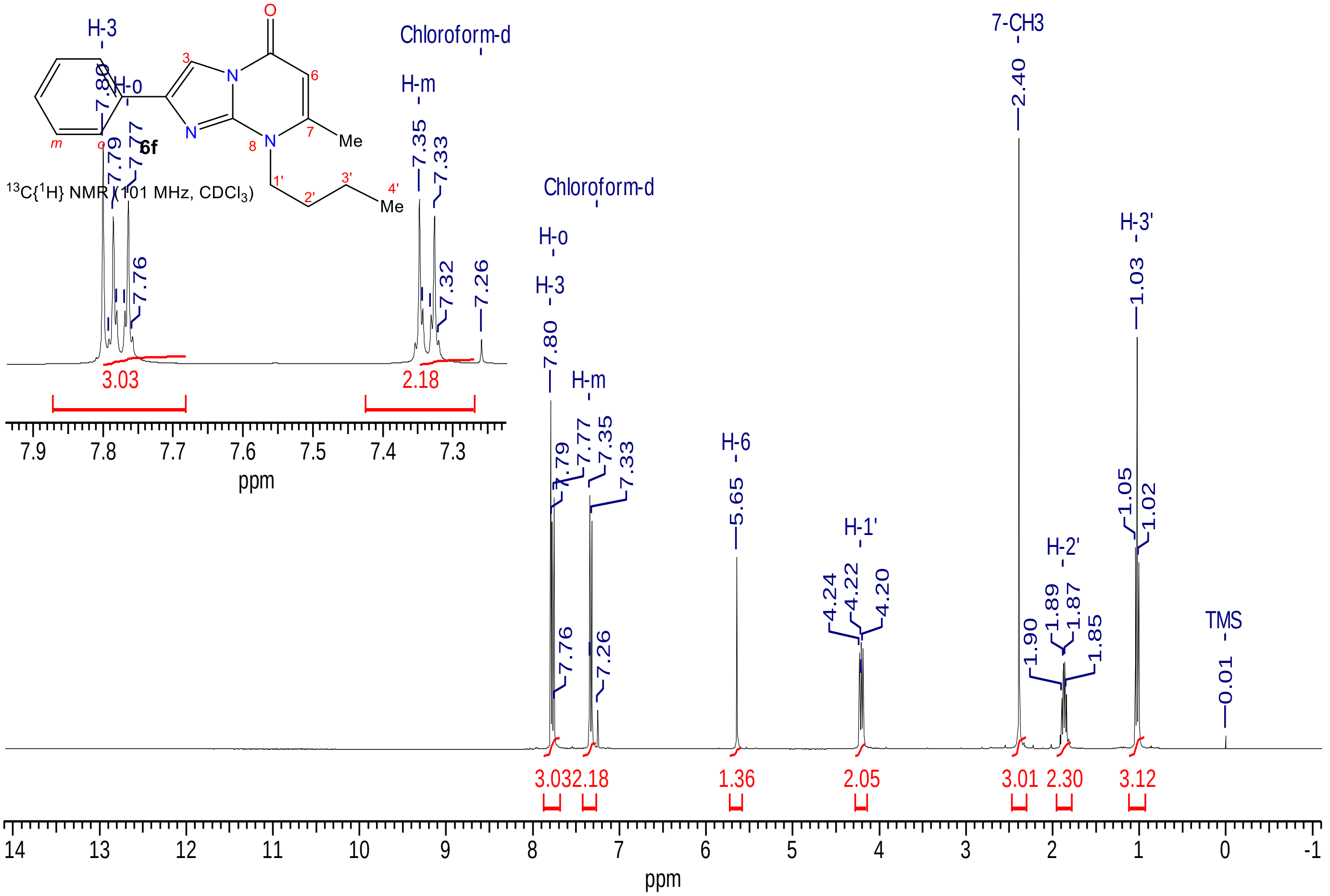

Fig. S15 ^{1}H and ^{13}C{^{1}H} NMR spectra of 8-butyl-7-methyl-2-phenylimidazo[1,2-*a*]pyrimidin-5(8*H*)-one (**6f**)

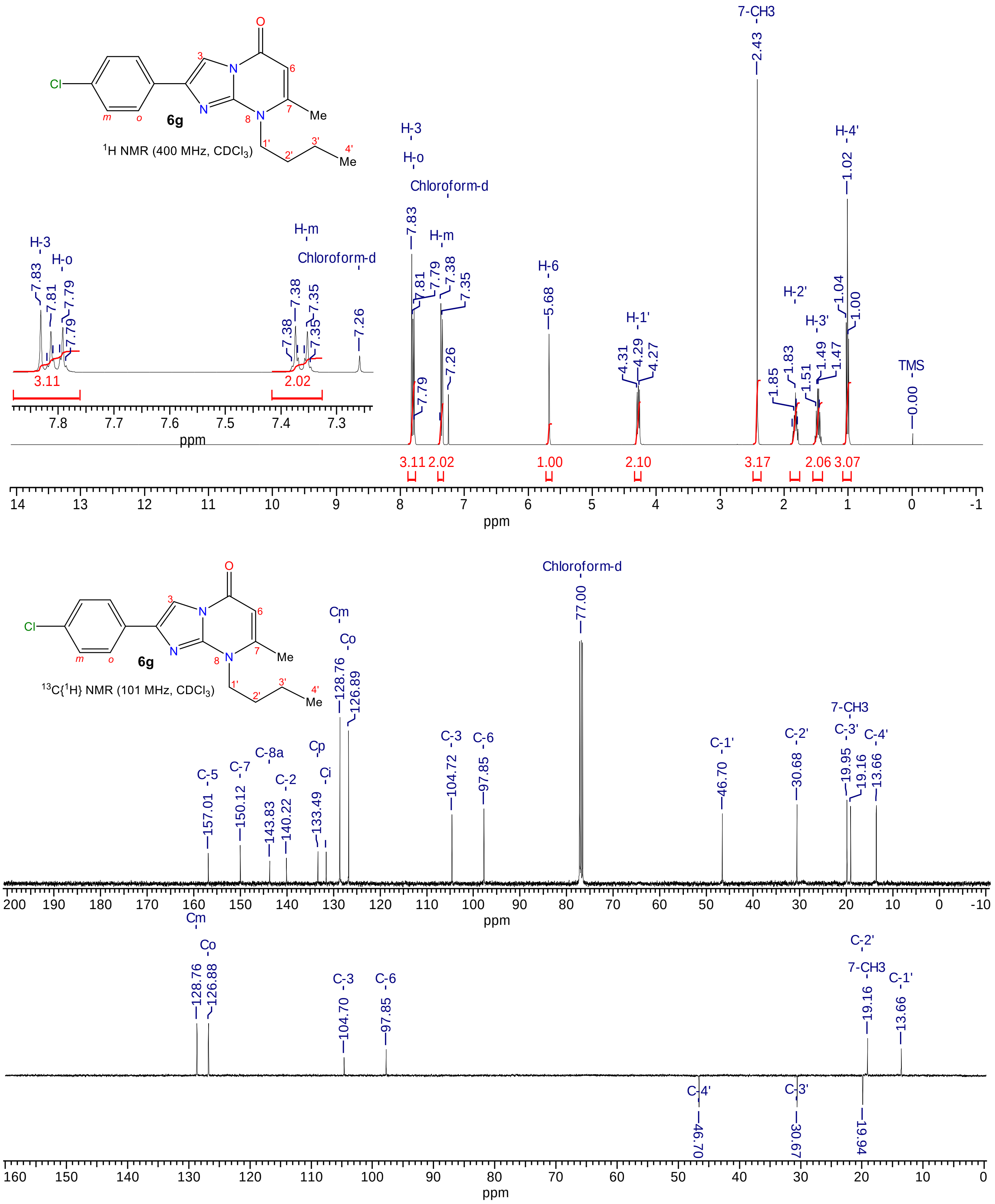

Fig. S16 ¹H and ¹³C{¹H} NMR spectra of 8-butyl-2-(4-chlorophenyl)-7-methylimidazo[1,2-*a*]pyrimidin-5(8*H*)-one (**6g**)

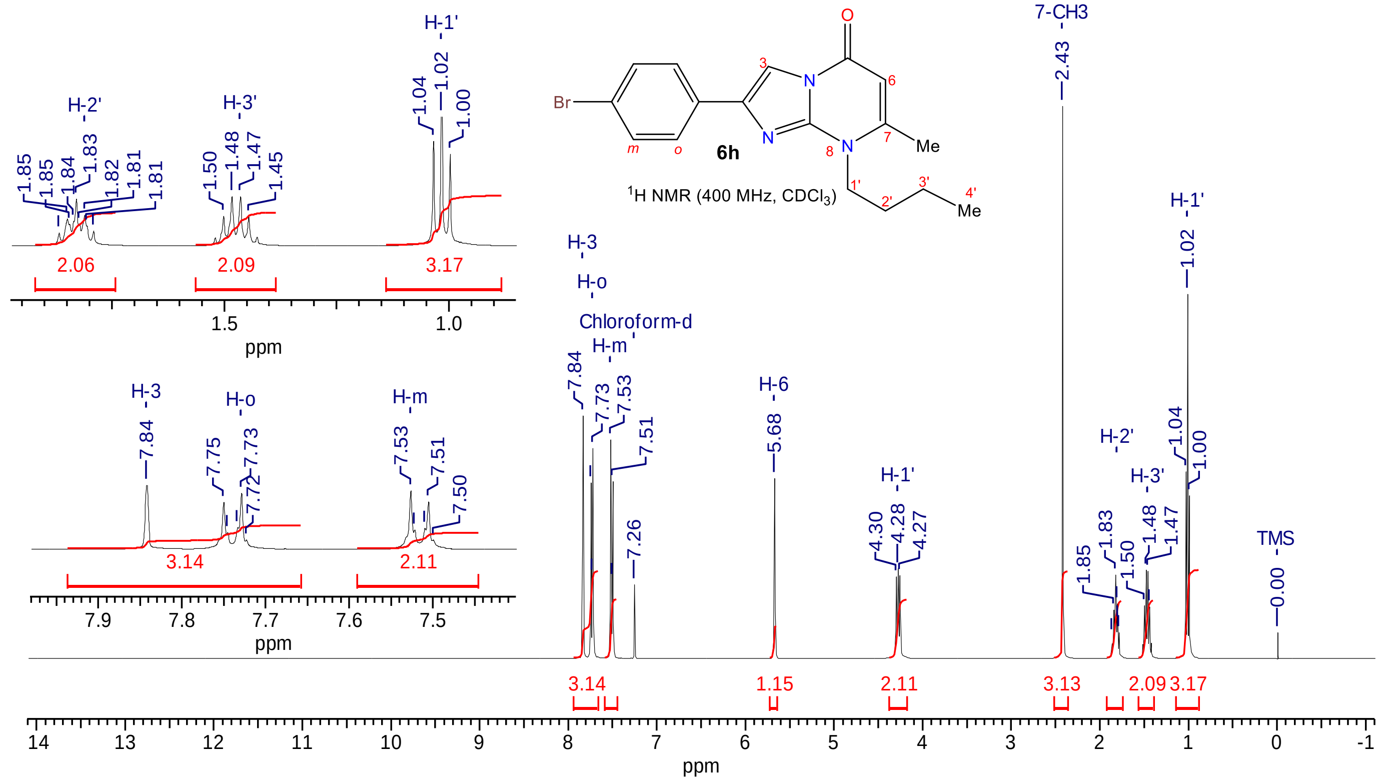

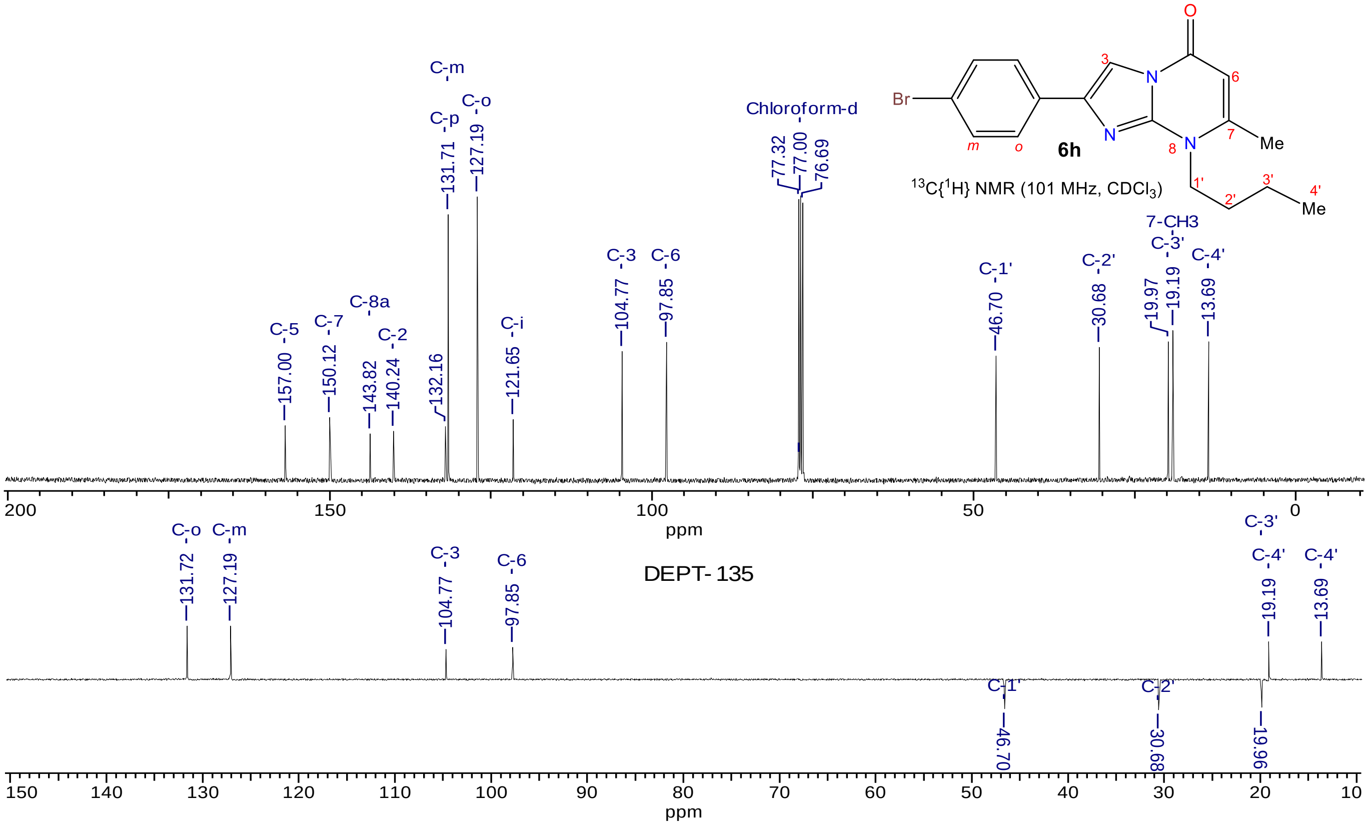

Fig. S17 ^{1}H and ^{13}C{^{1}H} NMR spectra of 8-butyl-(4-bromophenyl)-7-methylimidazo[1,2-*a*]pyrimidin-5(8*H*)-one (**6h**)

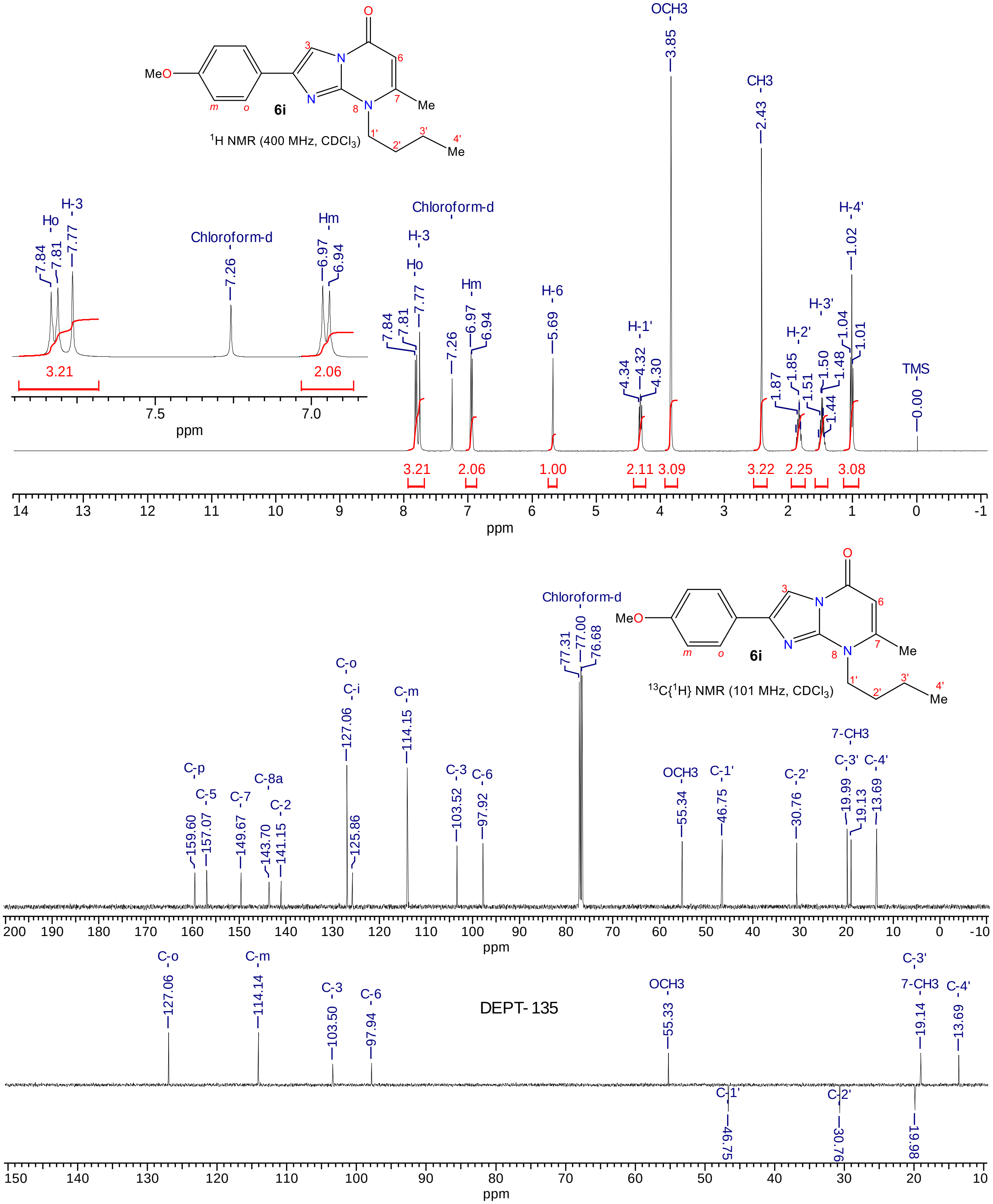

Fig S18 ^{1}H and $^{13}C\{^{1}H\}$ NMR spectra of 8-butyl-(4-methoxyphenyl)-7-methylimidazo[1,2-*a*]pyrimidin-5(8*H*)-one (**6i**)

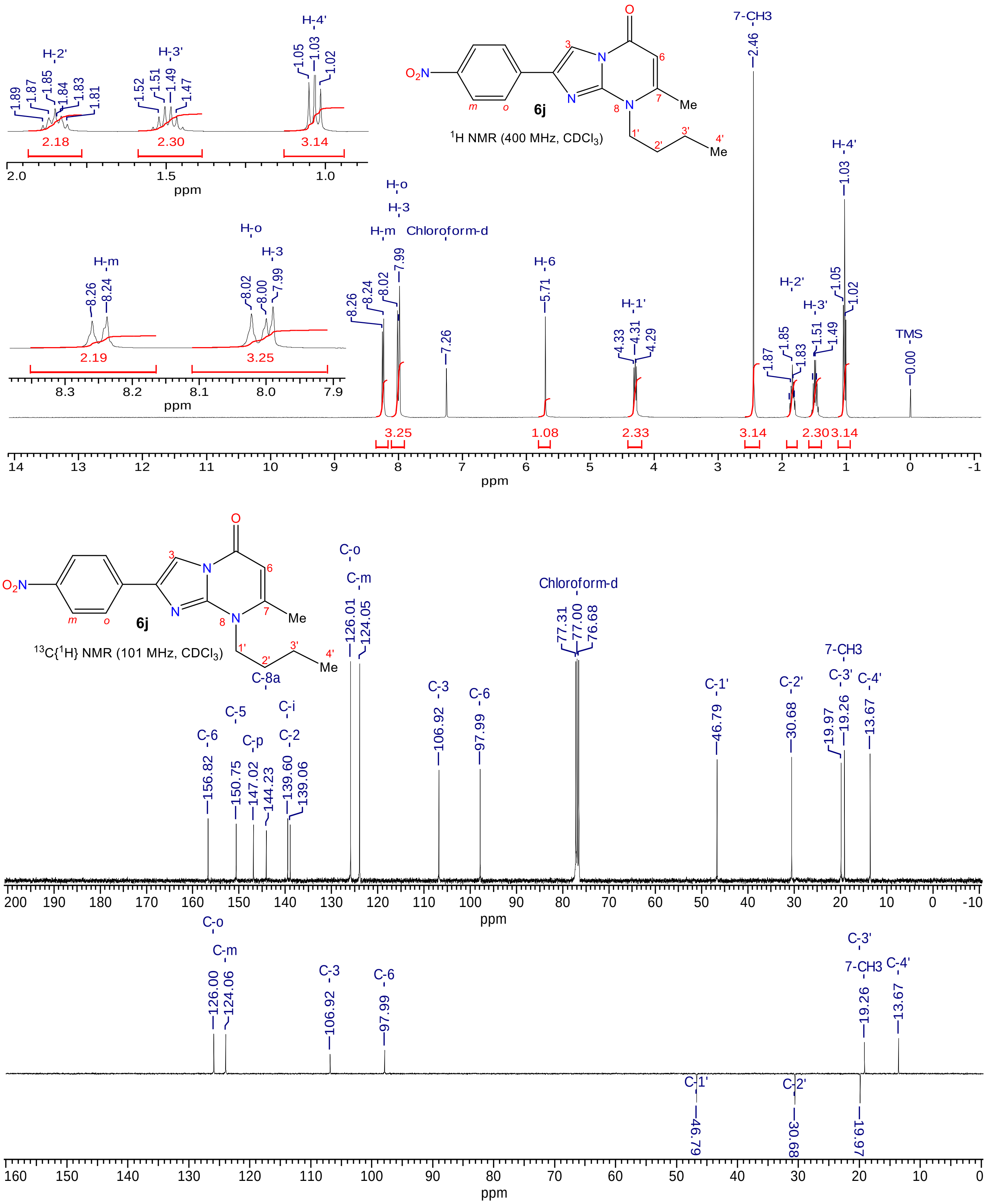

Fig. S19 ^{1}H and ^{13}C{^{1}H} NMR spectra of 8-butyl-7-methyl-2-(4-nitrophenyl)imidazo[1,2-*a*]pyrimidin-5(8*H*)-one (**6j**)

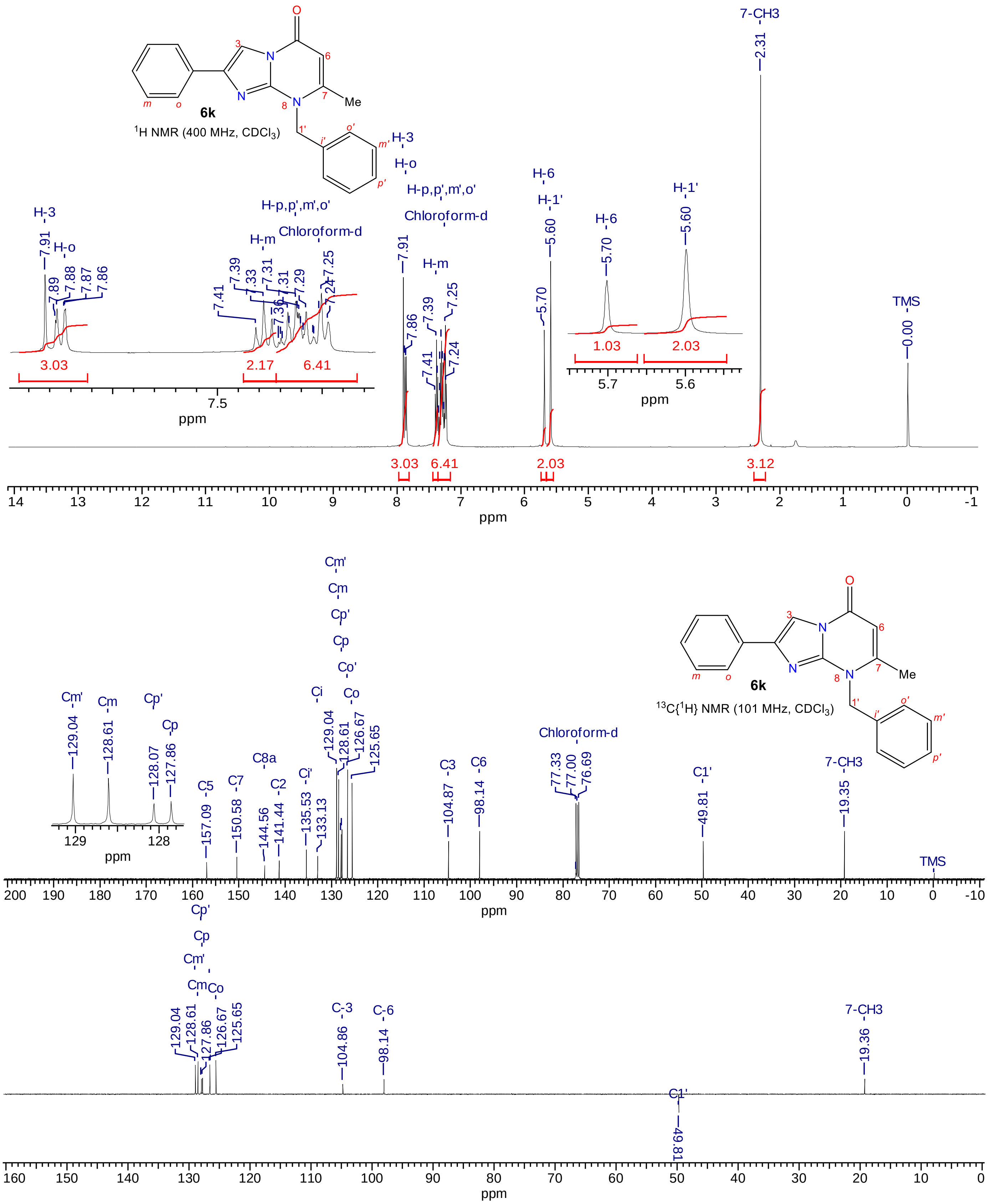

Fig. S20 ^{1}H and $^{13}C\{^{1}H\}$ NMR spectra of 8-benzyl-7-methyl-2-phenylimidazo[1,2-*a*]pyrimidin-5(8*H*)-one (**6k**)

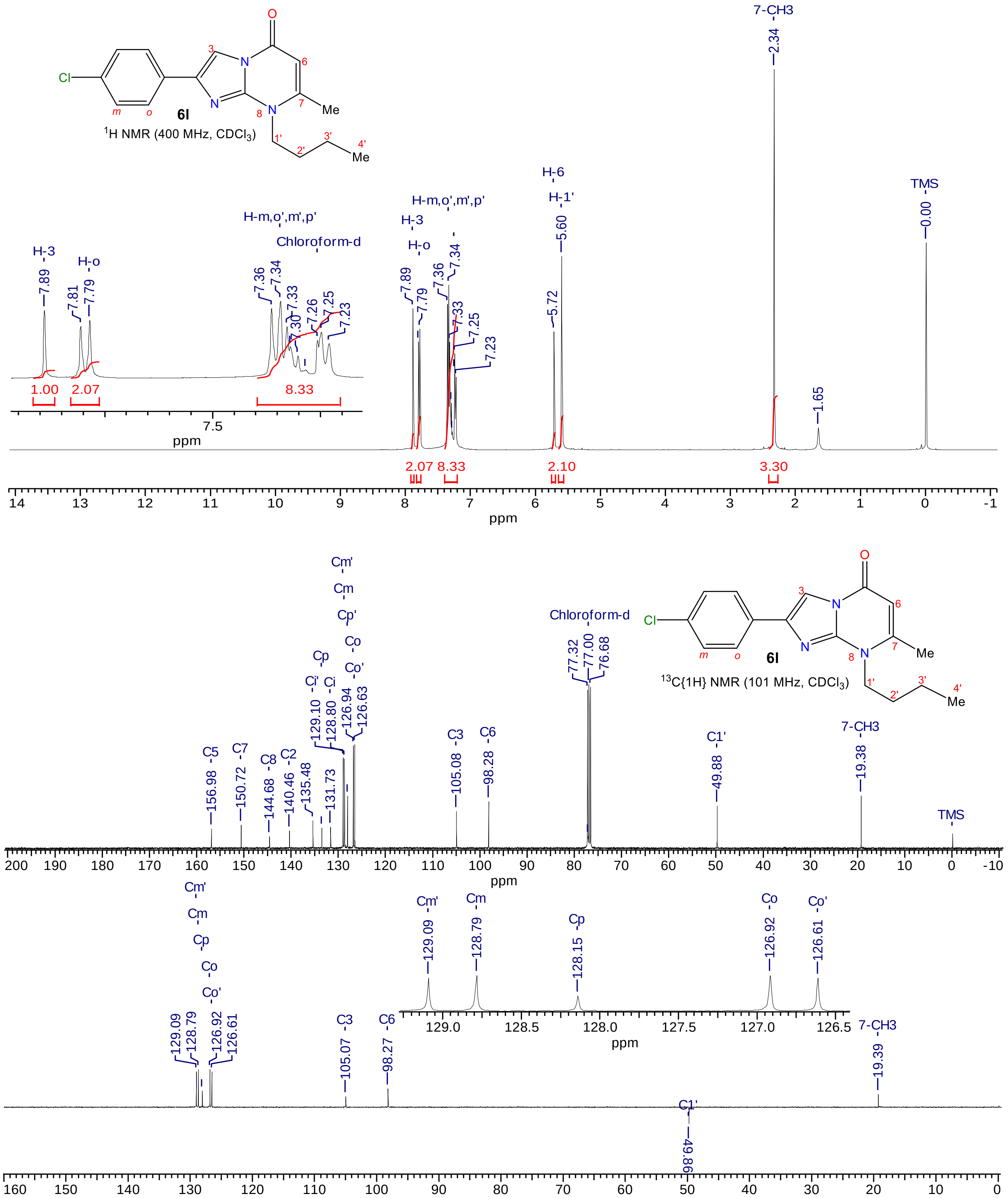

Fig. S21 ^{1}H and ^{13}C{^{1}H} NMR spectra of 8-benzyl-2-(4-chlorophenyl)-7-methylimidazo[1,2-*a*]pyrimidin-5(8*H*)-one (**4l**)

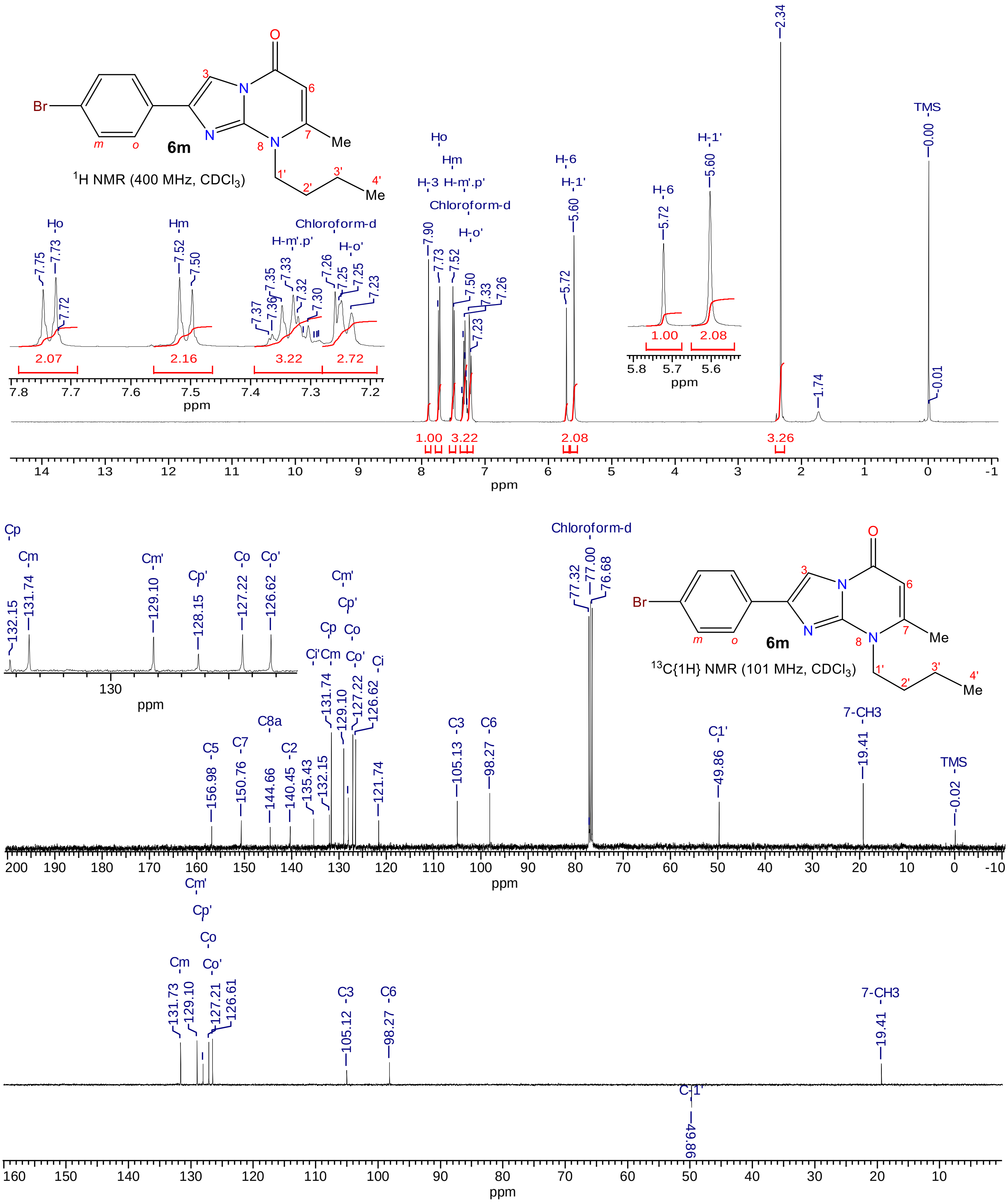

Fig. S22 ^{1}H and ^{13}C{^{1}H} NMR spectra of 8-benzyl-2-(4-bromophenyl)-7-methylimidazo[1,2-*a*]pyrimidin-5(8*H*)-one (**6m**)

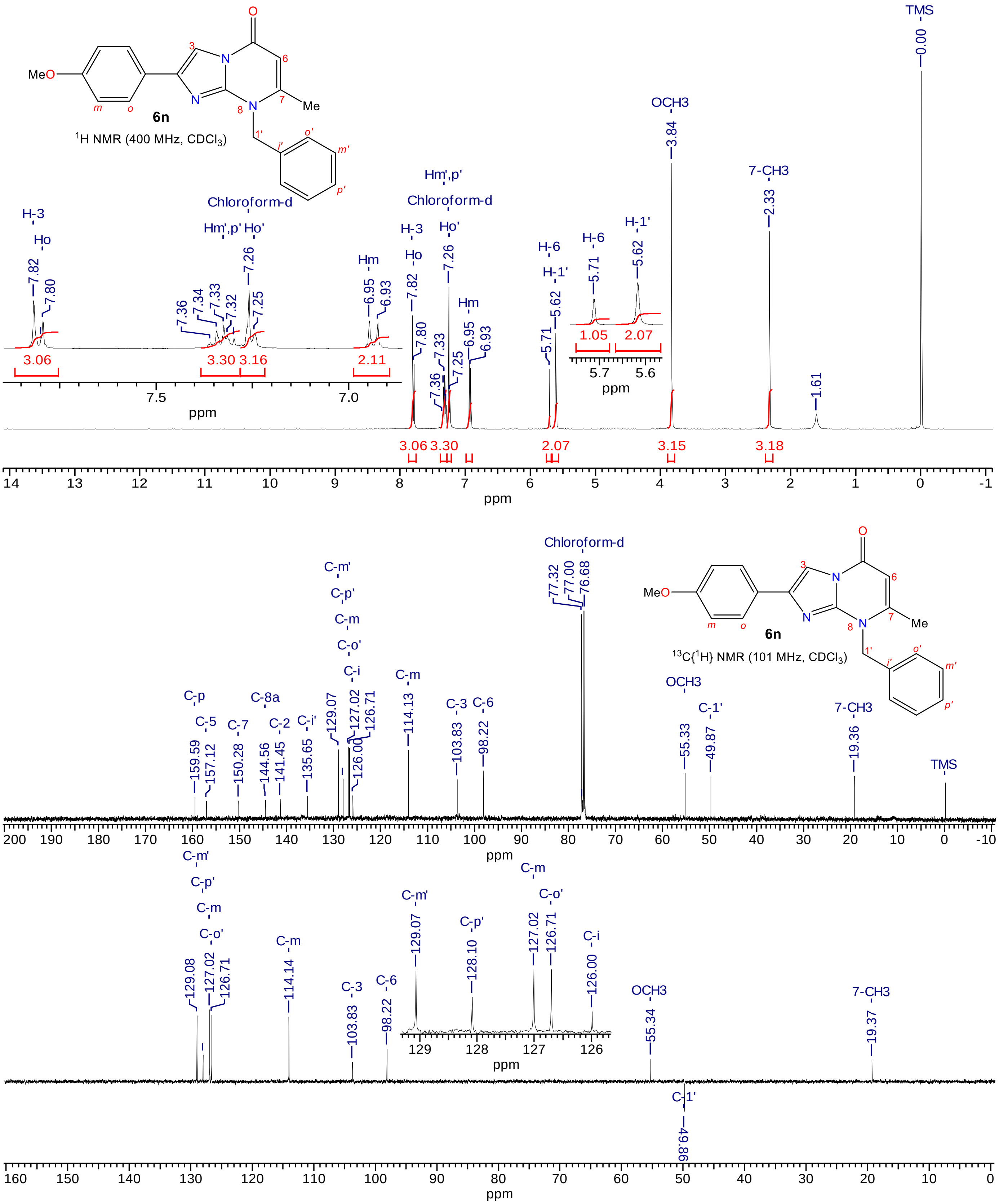

Fig. S23 ^{1}H and $^{13}C\{^{1}H\}$ NMR spectra of 8-benzyl-2-(4-methoxyphenyl)-7-methylimidazo[1,2-*a*]pyrimidin-5(8*H*)-one (**4n**)

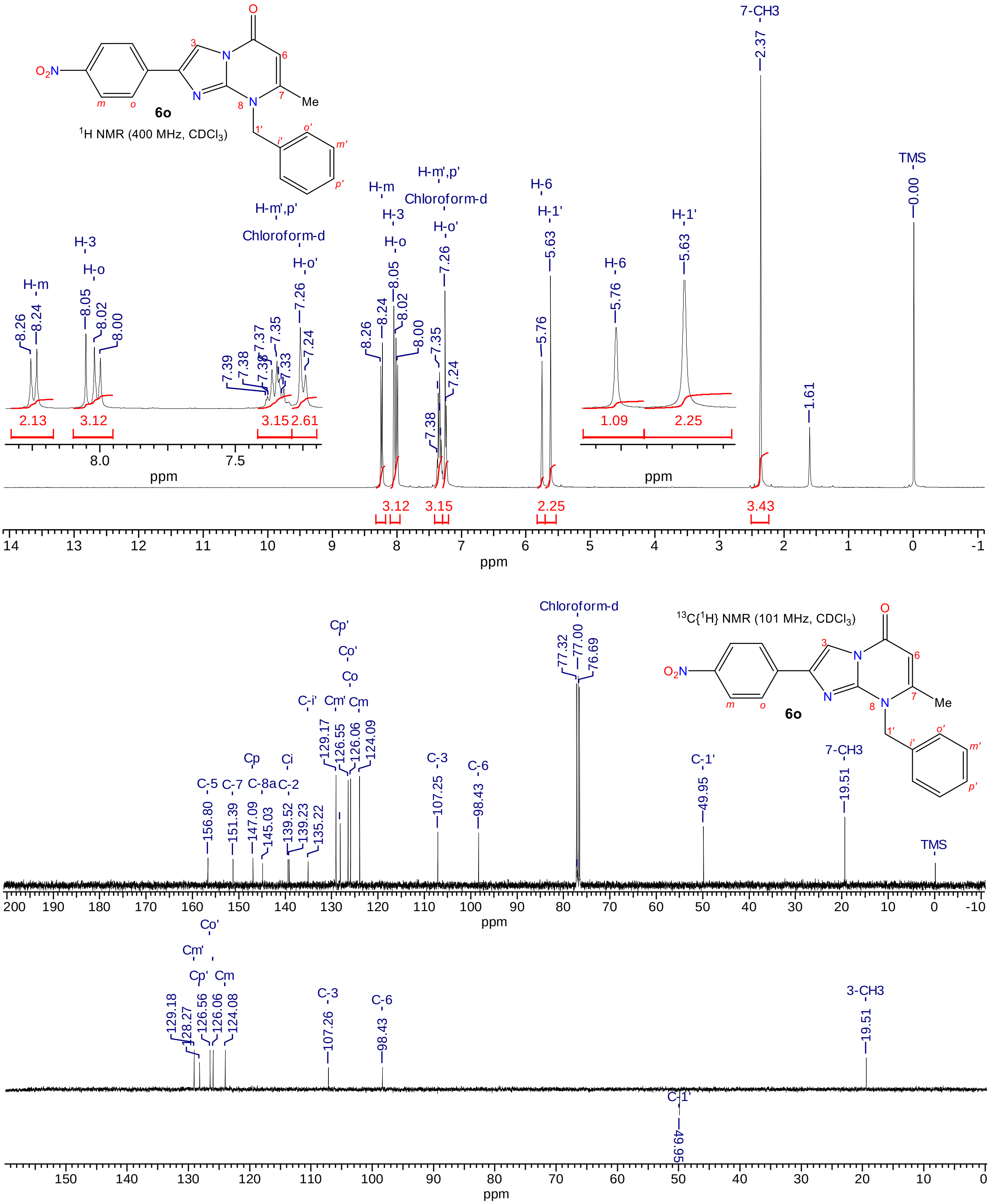

Fig. S24 ^1H and $^{13}C\{^1H\}$ NMR spectra of 8-benzyl-7-methyl-2-(4-nitrophenyl)imidazo[1,2-*a*]pyrimidin-5(8*H*)-one (**6o**)

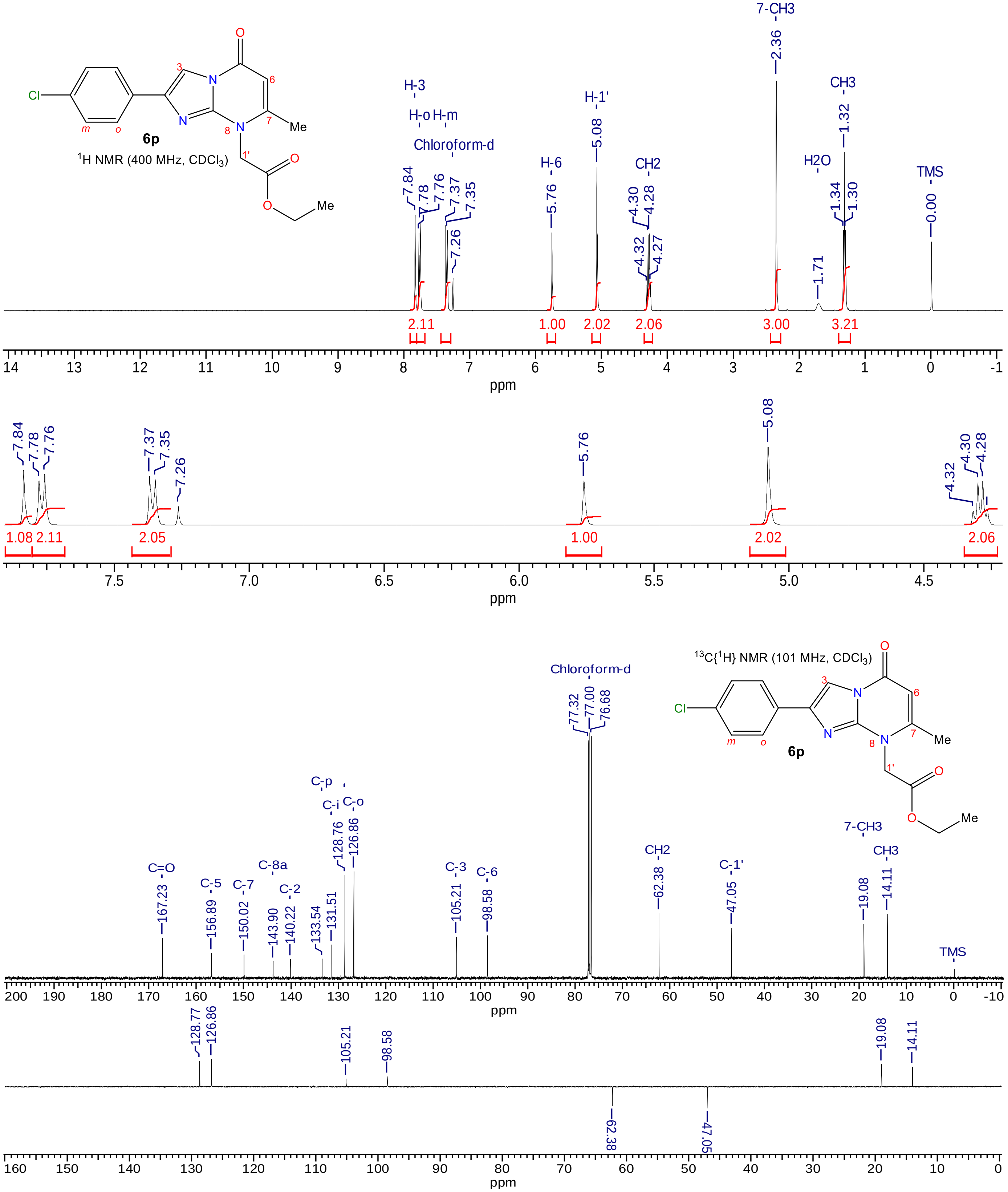

Fig. S25 ^{1}H and ^{13}C{^{1}H} NMR spectra of ethyl 2-(4-chlorophenyl)-7-methyl-5-oxoimidazo[1,2-*a*]pyrimidin-8(5*H*)-yl)acetate (**6p**)

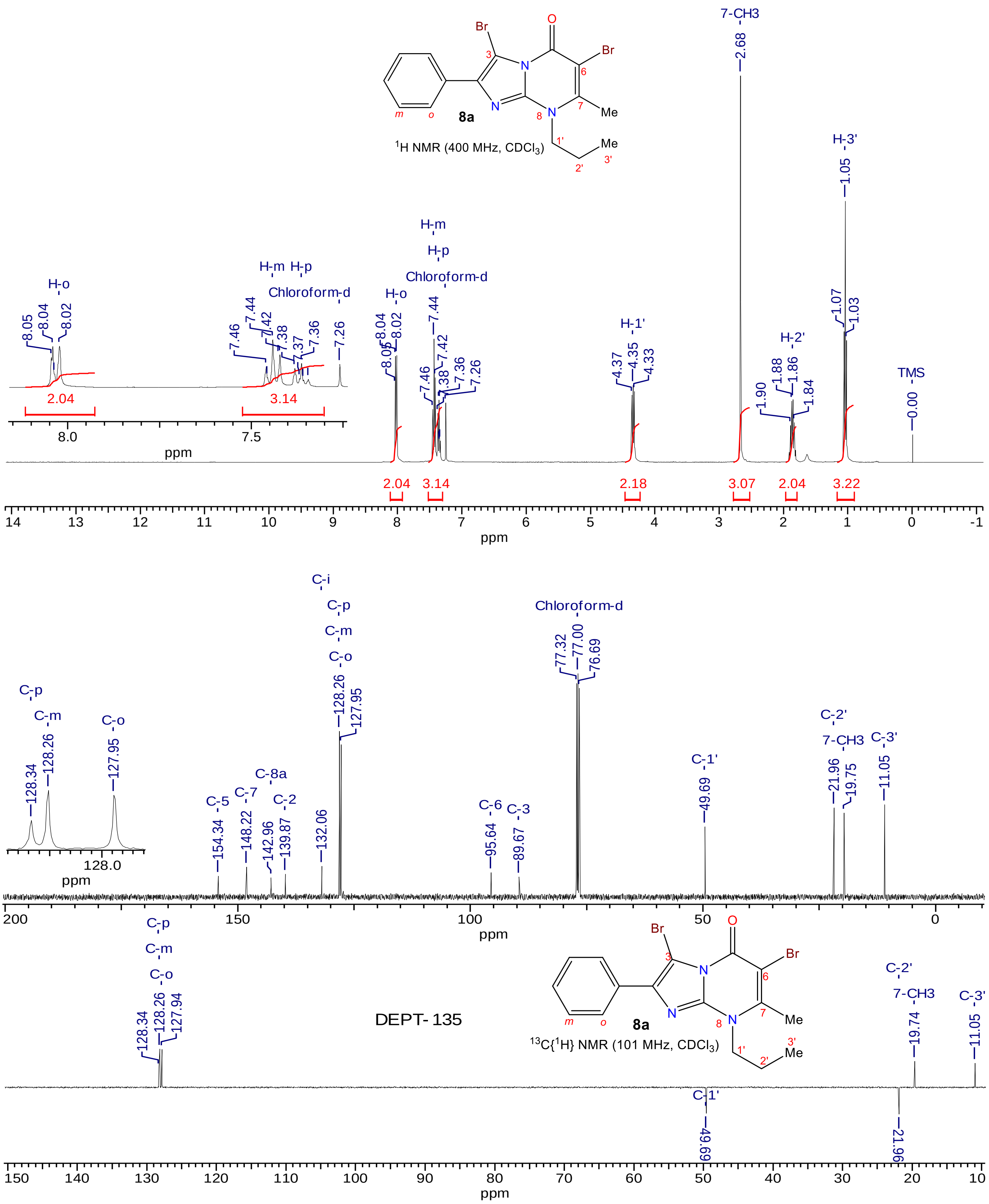

Fig. S26 ^{1}H and ^{13}C{^{1}H} NMR spectra of 3,6-dibromo-7-methyl-2-phenyl-8-propylimidazo[1,2-*a*]pyrimidin-5(8*H*)-one (**8a**)

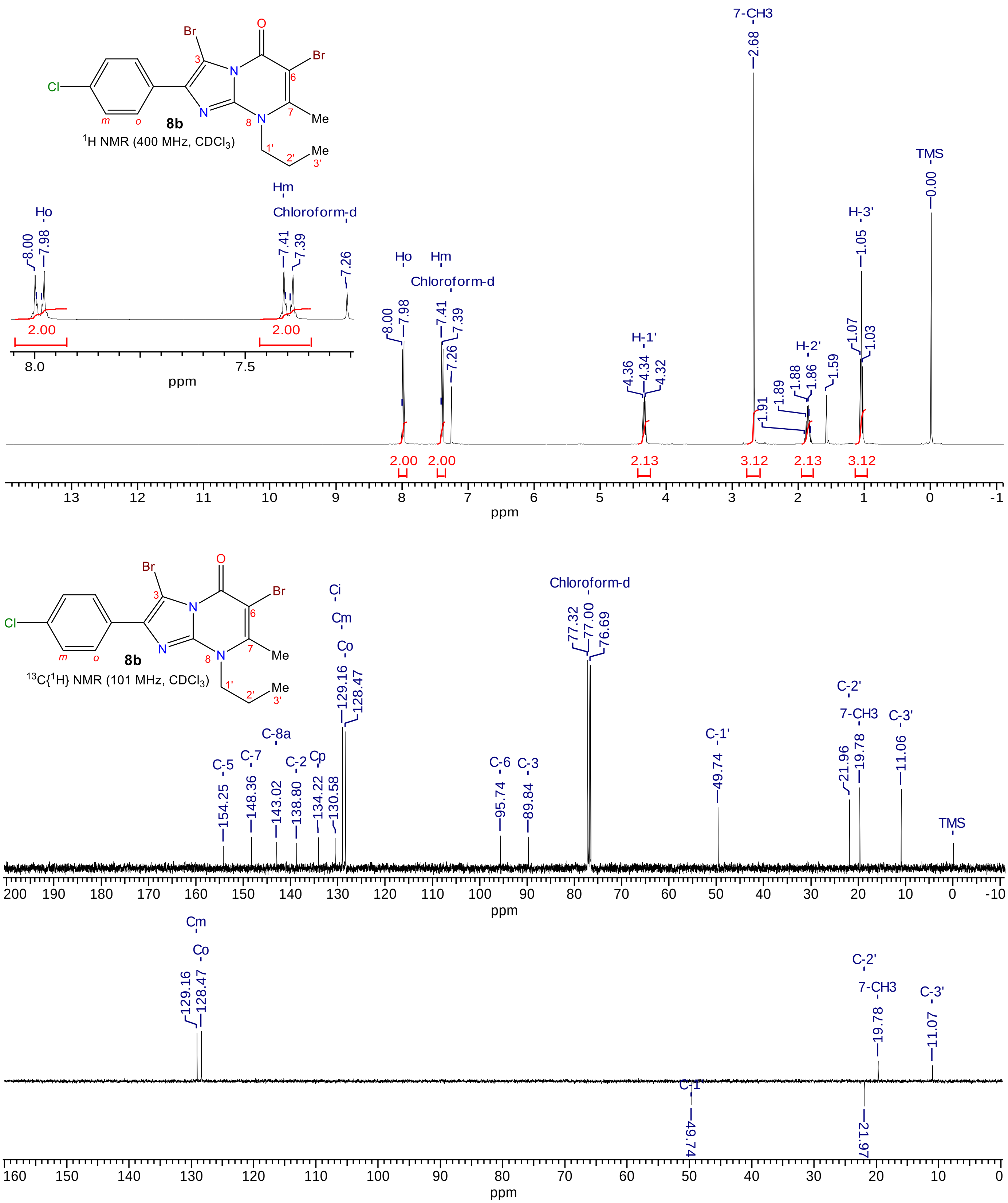

Fig. S27 ^{1}H and ^{13}C{^{1}H} NMR spectra of 3,6-dibromo-2-(4-chlorophenyl)-7-methyl-8-propylimidazo[1,2-*a*]pyrimidin-5(8*H*)-one (**8b**)

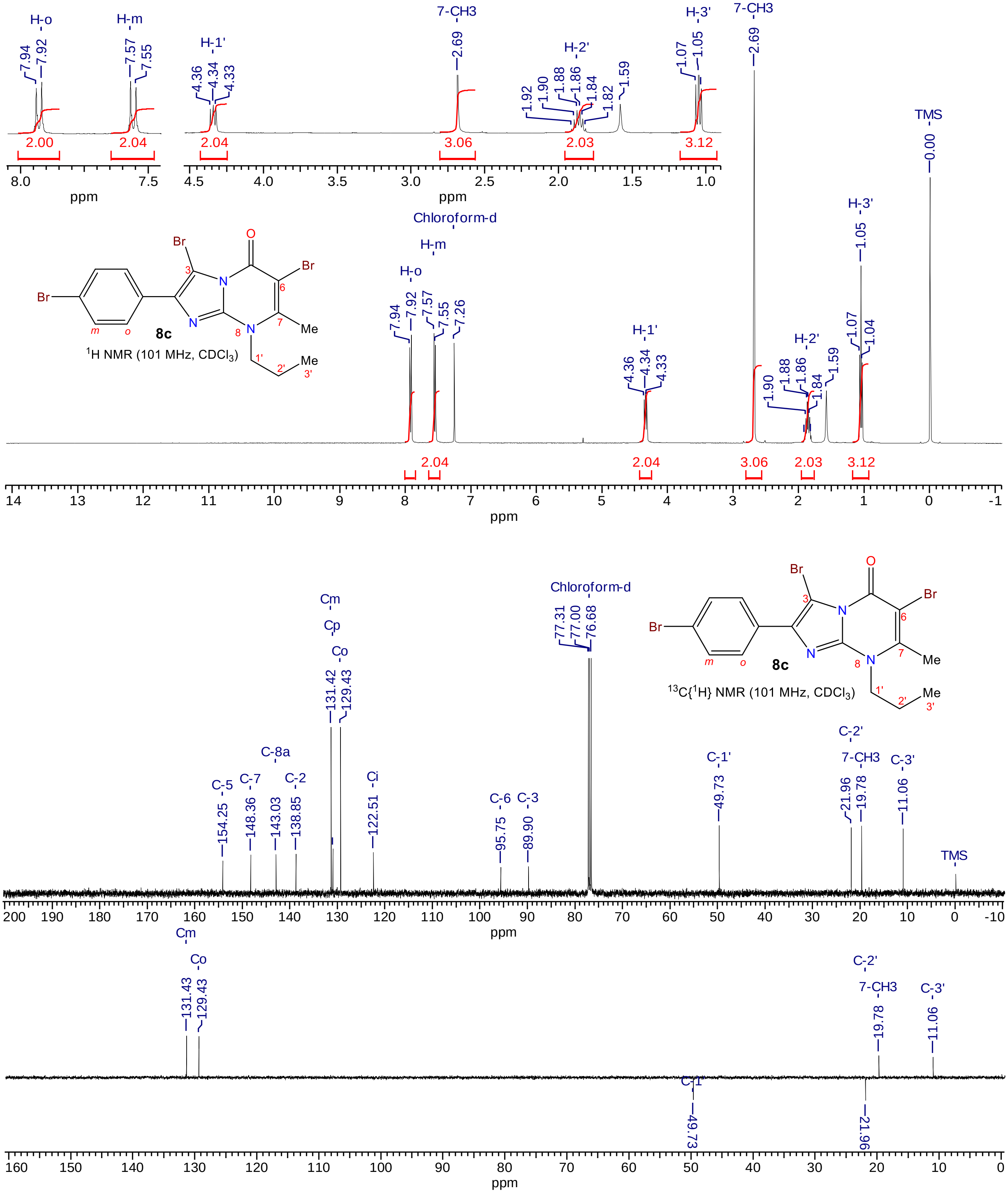

Fig. S28 ^{1}H and ^{13}C{^{1}H} NMR spectra of 3,6-dibromo-2-(4-bromophenyl)-7-methyl-8-propylimidazo[1,2-*a*]pyrimidin-5(8*H*)-one (**8c**)

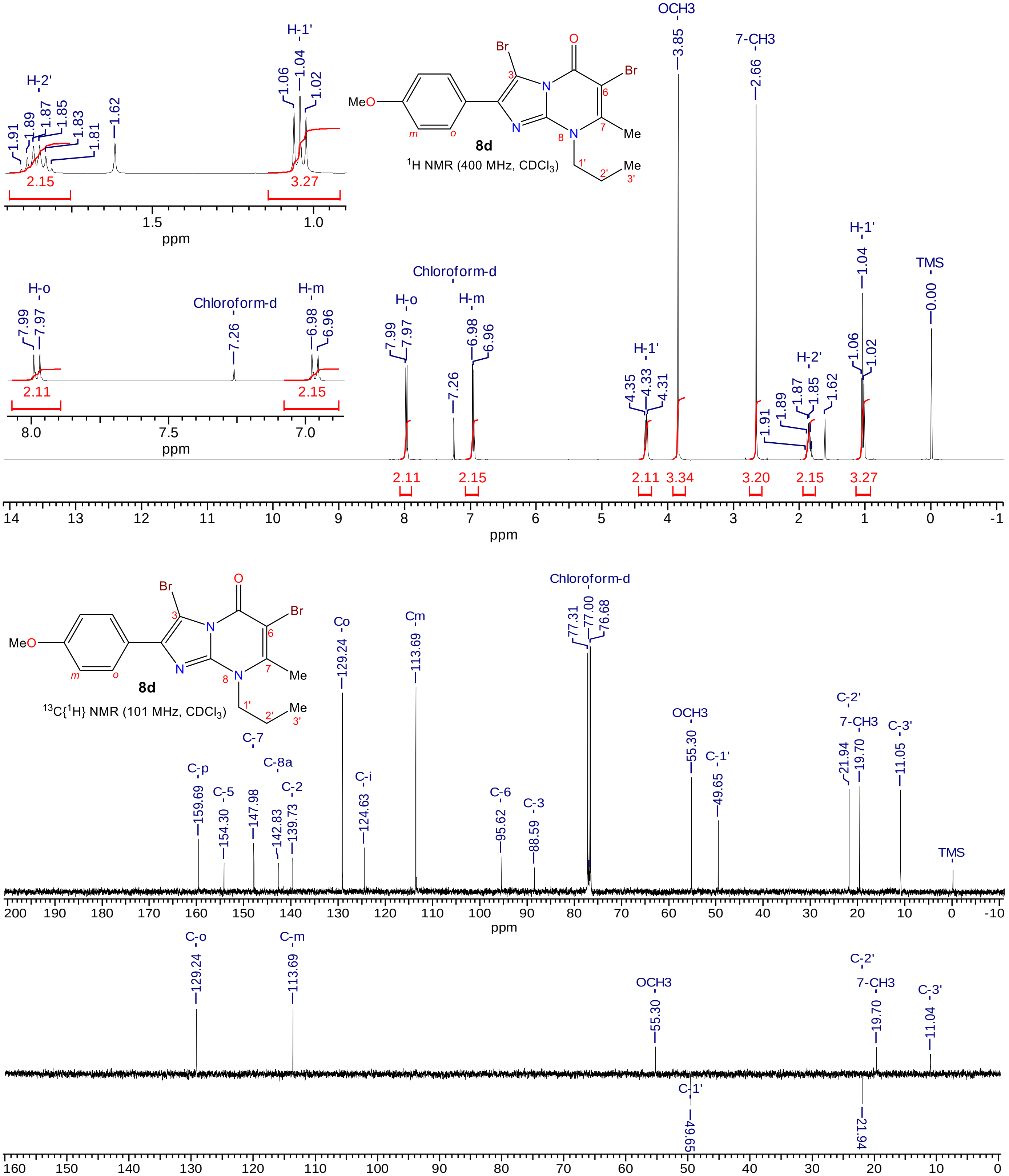

Fig. S29 ^{1}H and ^{13}C{^{1}H} NMR spectra of 3,6-dibromo-2-(4-methoxyphenyl)-7-methyl-8-propylimidazo[1,2-*a*]pyrimidin-5(8*H*)-one (**8d**)

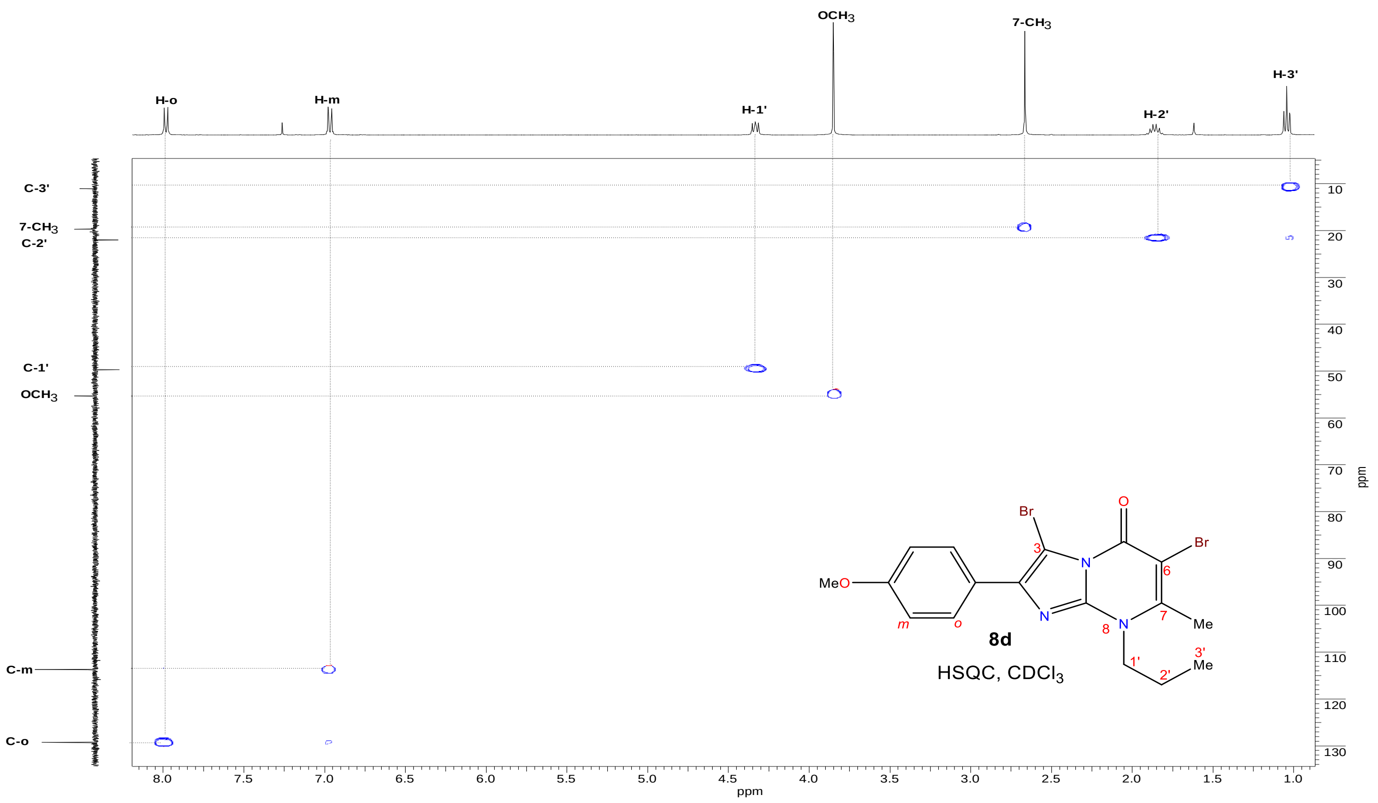

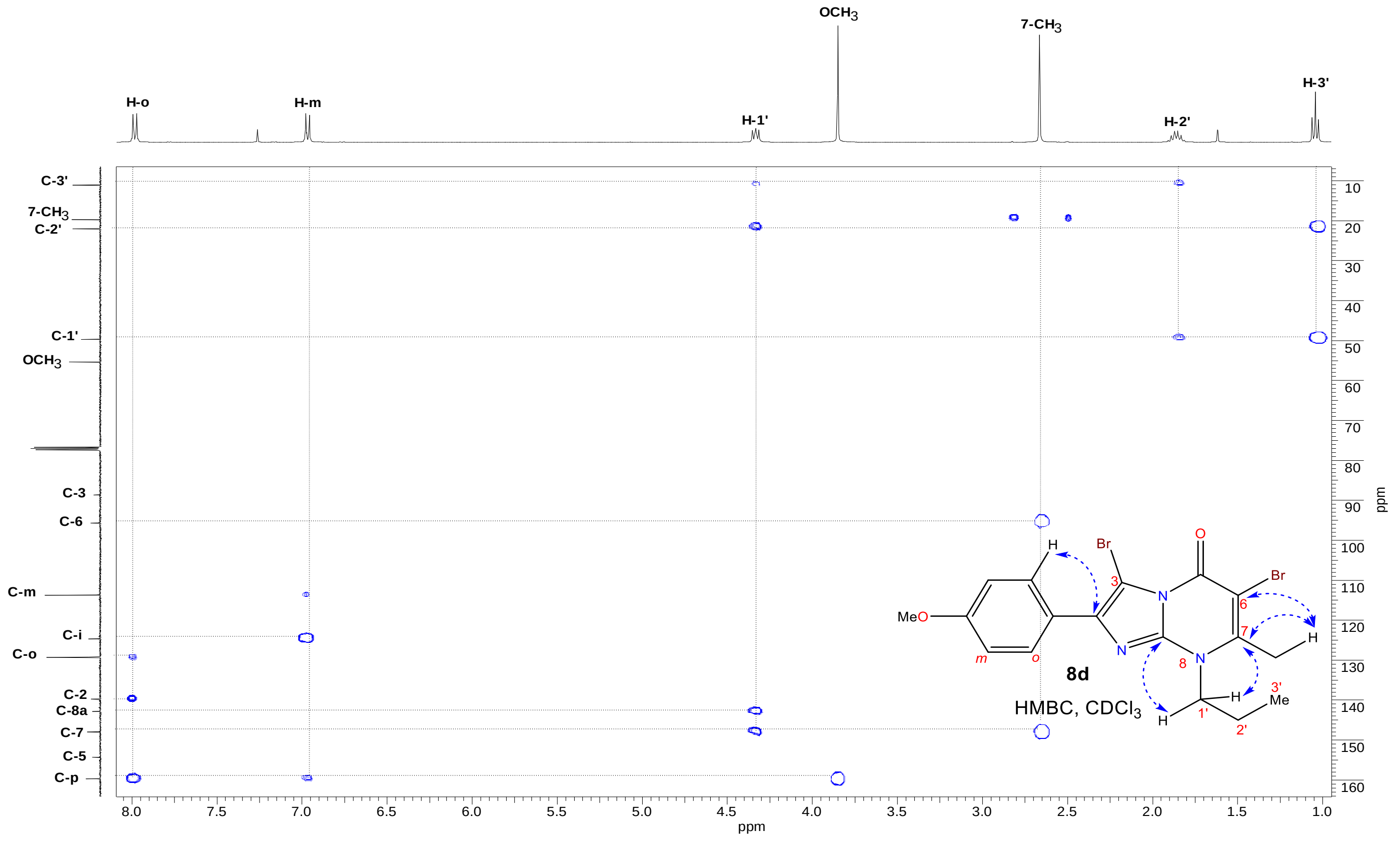

Fig. S30 HSQC and HMBC spectra of 3,6-dibrominated compound **8d**

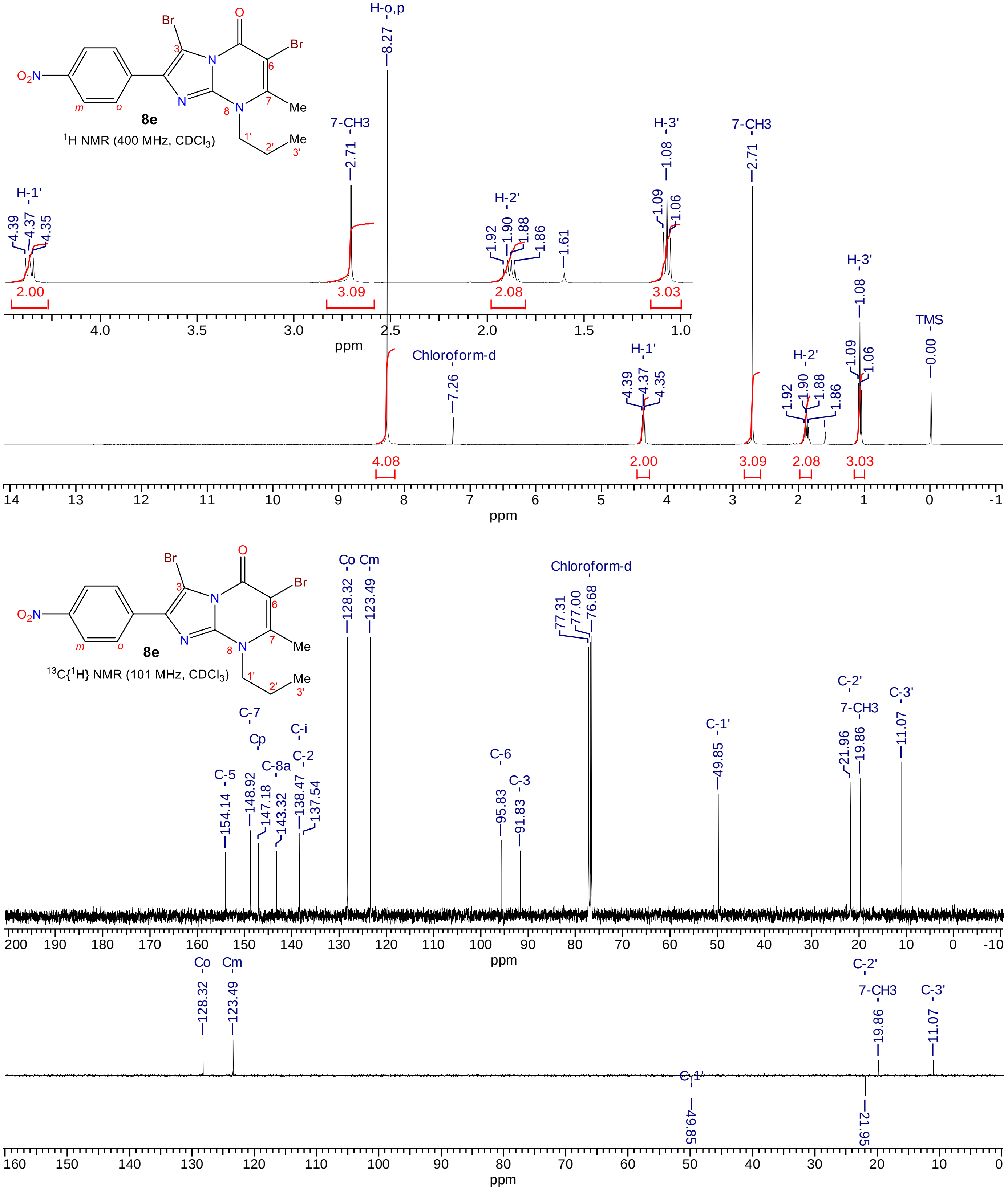

Fig. S31 ^{1}H and ^{13}C{^{1}H} NMR spectra of 3,6-dibromo-7-methyl-2-(4-nitrophenyl)-8-propylimidazo[1,2-*a*]pyrimidin-5(8*H*)-one (**8e**)

4. Photophysical and crystallographic details

UV-vis absorption and fluorescence studies of compound **6i** were carried out at 20 °C using solutions 6.6 µM in cyclohexene (CH), *tert*-butyl methyl ether (TBME), dichloromethane (DCM), ethyl acetate (AcOEt), *N,N*-dimethylformamide (DMF), and acetonitrile (ACN). The relative quantum yields (φ_F) were obtained using Prodan (φf = 0.94 in ACN) as a reference and calculated according to Equation 1

$$\phi_{f,x} = \phi_{f,st} \frac{F_x}{F_{st}} \frac{A_{st}}{A_x} \frac{n_x^2}{n_{st}^2} \qquad \textbf{Equation 1}$$

where *x* and *st* indicate the sample and standard solution, respectively, *F* is the integral photon flux, *A* is the absorption factor, and *n* is the refractive index of the solvent.[8]

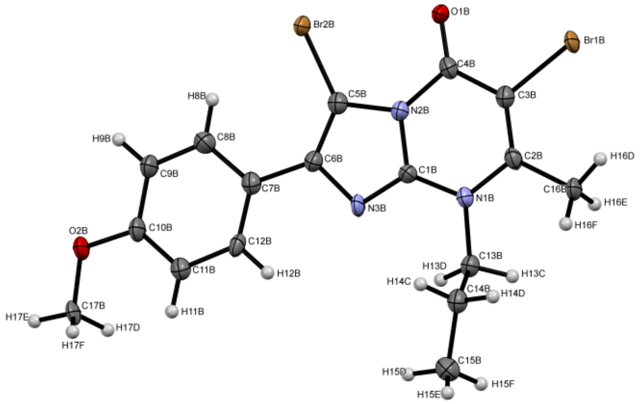

Fig. S32 ORTEP drawing for structure **8d**. Displacement ellipsoids are drawn at the 50% probability level.

5. References

1 G. M. Sheldrick, *Acta Crystallogr C Struct Chem*, 2015, **71**, 3–8.

2 N. R. Elejalde, M. Macías, J. C. Castillo, M. Sortino, L. Svetaz, S. Zacchino and J. Portilla, *ChemistrySelect*, 2018, **3**, 5220–5227.

3 M. Jyothi, H. A. Khamees, S. M. Patil, R. Ramu and S. A. Khanum, *Journal of the Iranian Chemical Society*, 2022, **19**, 3919–3933.

4 A. M. Kamal El-sagheir, I. Abdelmesseh Nekhala, M. K. Abd El-Gaber, A. S. Aboraia, J. Persson, A.-B. Schäfer, M. Wenzel and F. A. Omar, *RSC Med Chem*, 2023, **14**, 2593–2610.

5 S. Laneri, A. Sacchi and E. Abignente, *J Heterocycl Chem*, 2000, **37**, 1265–1267.

6 H. T. Abdel-Mohsen, A. Abood, K. J. Flanagan, A. Meindl, M. O. Senge and H. I. El Diwani, *Arch Pharm (Weinheim)*, 2020, **353**, e1900271.

7 M. Kawaguchi, T. Okabe, S. Okudaira, K. Hama, K. Kano, H. Nishimasu, H. Nakagawa, R. Ishitani, H. Kojima, O. Nureki, J. Aoki and T. Nagano, *J Med Chem*, 2020, **63**, 3188–3204.

8 C. Würth, M. Grabolle, J. Pauli, M. Spieles and U. Resch-Genger, *Nat. Protoc.*, 2013, **8**, 1535–1550.
